# Practices in sedation, analgesia, mobilization, delirium, and sleep deprivation in adult intensive care units (SAMDS-ICU): an international survey before and during the COVID-19 pandemic

**DOI:** 10.1186/s13613-022-00985-y

**Published:** 2022-02-04

**Authors:** Mariana Luz, Bruna Brandão Barreto, Roberta Esteves Vieira de Castro, Jorge Salluh, Felipe Dal-Pizzol, Caio Araujo, Audrey De Jong, Gérald Chanques, Sheila Nainan Myatra, Eduardo Tobar, Carolina Gimenez-Esparza Vich, Federico Carini, Eugene Wesley Ely, Joanna L. Stollings, Kelly Drumright, John Kress, Pedro Povoa, Yahya Shehabi, Wilson Mphandi, Dimitri Gusmao-Flores

**Affiliations:** 1 Intensive Care Unit of the Hospital da Mulher, Rua Barão de Cotegipe, 1153, Roma, Salvador, BA CEP: 40411-900 Brazil; 2 Programa de Pós-Graduação em Medicina e Saúde, Faculdade de Medicina da Bahia, Universidade Federal da Bahia, Salvador, Bahia Brazil; 3 Intensive Care Unit, Hospital Universitário Professor Edgard Santos, Salvador, Brazil; 4 Departamento de Pediatria, Hospital Universitário Pedro Ernesto, Universidade Do Estado Do Rio de Janeiro, Rio de Janeiro, Brazil; 5 Department of Critical Care and Postgraduate Program in Translational Medicine, D’Or Institute for Research and Education (IDOR), Rio de Janeiro, Brazil; 6 Programa de Pós-Graduação em Clínica Médica, Universidade Federal do Rio de Janeiro, Rio de Janeiro, Brazil; 7 Laboratório de Fisiopatologia Experimental, Programa de Pós-Graduação em Ciências da Saúde, Universidade do Extremo Sul Catarinense, Criciúma, Santa Catarina Brazil; 8 Faculdade de Medicina da Bahia, Universidade Federal da Bahia, Salvador, BA Brazil; 9 Department of Anesthesia and Intensive Care Unit, Regional University Hospital of Montpellier, St-Eloi Hospital, University of Montpellier, PhyMedExp, INSERM U1046, CNRS UMR, 9214 Montpellier, CEDEX 5, France; 10 Department of Anaesthesiology, Critical Care and Pain, Tata Memorial Hospital, Homi Bhabha National Institute, Mumbai, India; 11 Internal Medicine Department, Critical Care Unit, Hospital Clínico Universidad de Chile, Santiago, Chile; 12 Critical Care Department, Vega Baja Orihuela Hospital, Alicante, Spain; 13 Intensive Care Unit, Hospital Italiano de Buenos Aires, Buenos Aires, Argentina; 14 Division of Allergy, Pulmonary, and Critical Care Medicine, Vanderbilt University Medical Center, Nashville, TN USA; 15 Center for Health Services Research, Vanderbilt University Medical Center, Nashville, TN USA; 16 Center for Quality Aging, Vanderbilt University Medical Center, Nashville, TN USA; 17 Geriatric Research Education and Clinical Center (GRECC) Service at the Department of Veterans Affairs Medical Center, Tennessee Valley Healthcare System, Nashville, TN USA; 18 Critical Illness, Brain Dysfunction, and Survivorship (CIBS) Center, Vanderbilt University Medical Center, Nashville, TN USA; 19 Department of Pharmaceutical Services, Vanderbilt University Medical Center, Nashville, TN USA; 20 Tennessee Valley Healthcare System VA Medical Center, Nashville, TN USA; 21 Division of Pulmonary and Critical Care Medicine, University of Chicago, Chicago, IL USA; 22 Polyvalent Intensive Care Unit, Hospital de São Francisco Xavier, CHLO, Lisbon, Portugal; 23 CHRC, CEDOC, NOVA Medical School, New University of Lisbon, Lisbon, Portugal; 24 Center for Clinical Epidemiology and Research Unit of Clinical Epidemiology, OUH Odense University Hospital, Odense, Denmark; 25 Department of Critical Care and Perioperative Medicine, School of Clinical Sciences, Monash University, Melbourne, VIC Australia; 26 Intensive Care Unit, Hospital Américo Boavida, Luanda, Angola

**Keywords:** Sedation, Analgesia, Mobilization, Delirium, Sleep deprivation, COVID-19

## Abstract

**Background:**

Since the publication of the 2018 Clinical Guidelines about sedation, analgesia, delirium, mobilization, and sleep deprivation in critically ill patients, no evaluation and adequacy assessment of these recommendations were studied in an international context. This survey aimed to investigate these current practices and if the COVID-19 pandemic has changed them.

**Methods:**

This study was an open multinational electronic survey directed to physicians working in adult intensive care units (ICUs), which was performed in two steps: before and during the COVID-19 pandemic.

**Results:**

We analyzed 1768 questionnaires and 1539 (87%) were complete. Before the COVID-19 pandemic, we received 1476 questionnaires and 292 were submitted later. The following practices were observed before the pandemic: the Visual Analog Scale (VAS) (61.5%), the Behavioral Pain Scale (BPS) (48.2%), the Richmond Agitation Sedation Scale (RASS) (76.6%), and the Confusion Assessment Method for the Intensive Care Unit (CAM-ICU) (66.6%) were the most frequently tools used to assess pain, sedation level, and delirium, respectively; midazolam and fentanyl were the most frequently used drugs for inducing sedation and analgesia (84.8% and 78.3%, respectively), whereas haloperidol (68.8%) and atypical antipsychotics (69.4%) were the most prescribed drugs for delirium treatment; some physicians regularly prescribed drugs to induce sleep (19.1%) or ordered mechanical restraints as part of their routine (6.2%) for patients on mechanical ventilation; non-pharmacological strategies were frequently applied for pain, delirium, and sleep deprivation management. During the COVID-19 pandemic, the intensive care specialty was independently associated with best practices. Moreover, the mechanical ventilation rate was higher, patients received sedation more often (94% versus 86.1%, *p* < 0.001) and sedation goals were discussed more frequently in daily rounds. Morphine was the main drug used for analgesia (77.2%), and some sedative drugs, such as midazolam, propofol, ketamine and quetiapine, were used more frequently.

**Conclusions:**

Most sedation, analgesia and delirium practices were comparable before and during the COVID-19 pandemic. During the pandemic, the intensive care specialty was a variable that was independently associated with the best practices. Although many findings are in accordance with evidence-based recommendations, some practices still need improvement.

**Supplementary Information:**

The online version contains supplementary material available at 10.1186/s13613-022-00985-y.

## Background

Critically ill patients frequently report heavy symptom burden during intensive care unit (ICU) stay [1, 2]. Pain is reported by 38–51% of patients who are at risk of dying, and it is considered one of the most distressful symptoms, along with shortness of breath and confusion [3]. Delirium remains highly prevalent among critically ill patients, and it is associated with both worse short- and long-term outcomes, such as in-hospital mortality, longer duration of mechanical ventilation (MV) and longer ICU and hospital stay, in addition to cognitive impairment [4–6]. Sedatives and analgesics (including opiates) are the most prescribed drugs in the ICU, with more than 90% of patients receiving them at some point during their stay [7, 8]. However, despite the need to control symptoms, relieve anxiety, and improve ventilator synchrony through the use of these drugs, these medications are also associated with increased risk of adverse events such as delirium, hospital-acquired infections, and increased mortality [9–13].

Therefore, judicious use of sedatives, analgesics, and psychoactive drugs is promoted by the 2018 Clinical Practice Guidelines for the Prevention and Management of Pain, Agitation/Sedation, Delirium, Immobility, and Sleep Disruption in Adult Patients in the ICU (PADIS) [14]. This guideline was expanded to include rehabilitation/mobilization and sleep improvement, due to their relation to delirium and sedative use. The aim was to promote evidence-based and patient-centered interventions for the prevention and management of pain, agitation/sedation, delirium, immobility, and sleep disruption in adult patients in the ICU [14].

Before the publication of the 2018 guidelines, the worldwide survey of Morandi et al*.* [15] aiming to assess the knowledge and use of all aspects of the ‘ABCDEF’ bundle (**A**ssess, Prevent, and Manage Pain [**A**], **B**oth Spontaneous Awakening Trials and Spontaneous Breathing Trials [**B**], **C**hoice of Analgesia and Sedation [**C**], **D**elirium: Assess, Prevent, and Manage [**D**], **E**arly Mobility and Exercise [**E**], and **F**amily Engagement and Empowerment [**F**]) showed improved delirium knowledge and practices for sedation and analgesia. Adherence to recommendations remained suboptimal and high variability among countries and regions was found.

Due to the surge of severely ill patients during the coronavirus 2019 (COVID-19) pandemic, drug shortages and disruption of ICU organization (such as lower staffing levels), the implementation of protocols, including those related to sedation, analgesia and delirium practices, might have been impacted by the coronavirus 2019 disease (COVID-19) pandemic [16–18].

The main aim of the present study was to evaluate the practices of sedation, analgesia, delirium investigation and treatment, mobilization and sleep improvement among physicians who work in adult ICUs worldwide, before and during the COVID-19 pandemic. The secondary aims were to assess whether there was any difference in practices by period (before and during the pandemic), by continent and by intensive care specialist or not in the studied population.

## Methods

This study was performed in two steps: (1) the first step involved survey development and administration and data analyses before the COVID-19 pandemic and (2) the second one, designed a posteriori, was the administration of this survey during the pandemic to physicians who work in COVID-19 ICUs.

### Survey development

A group of intensive care specialists (steering committee members) considered all potential items for inclusion in the questionnaire after reviewing the literature and participating in focus-group sessions. Item reduction was performed during sessions, resulting in a self-applied electronic questionnaire with 54 questions regarding the ICU profiles, professional expertise, and practices in sedation-, analgesia-, delirium screening-, and treatment-related, early mobilization and sleep improvement practices. Different types of questions were used, including demographic, Likert scale, and multiple-choice, and were distributed over 27 pages. They were not randomized or alternated. It was possible to choose more than one option in some multiple-choice questions. The number of items per page was variable, and adaptative questioning was used. It was not possible to skip questions concerning practices nor was it possible to review them after moving on to the next page.

Pre-testing was carried out using the “thinking aloud” technique (in which respondents are asked to verbalize thoughts while answering a question) to ensure adequate understanding of it. Pilot testing was performed to assure validity and, in this phase, intensivists with experience in clinical research were asked to answer all questions using an Internet survey format. Questions considered irrelevant or difficult to understand were reformulated or deleted. The time taken to answer each question was registered, and questions requiring more than 1 min to answer were reassessed. In the second phase, members of the steering committee, whose first language was English, French, and/or Spanish, translated the questionnaire. English, French, Spanish, and Portuguese versions are available in Additional files 1, 2, 3, 4. Pilot test responses were not included in the main results of the research.

In the second step of the survey, during the COVID-19 pandemic, the same questionnaire was administered to physicians who worked in COVID-19 ICUs. However, questions about mobilization and sleep improvement were excluded, in order to focus on sedation, analgesia and delirium management practices (Additional files 5, 6, 7, 8).

### Survey administration

The web link of the survey and a cover letter were distributed via the mailing list of the cooperative network for research of the *Associação de Medicina Intensiva Brasileira* (AMIB-Net) and the Brazilian Research in Intensive Care Network (BricNet) to physicians who worked in adult ICUs. The researchers from different centers in Latin America, the United States of America (US), Europe, Asia, Africa, and Oceania also distributed the survey to personal mailings and social media of their colleagues, and respondents were encouraged to share the survey link with their peers. The *Société Française d’Anesthésie et de Réanimation* (SFAR), *Sociedad Española de Medicina Intensiva, Crítica y Unidades Coronarias* (SEMICYUC), *Sociedad* Argentina *de Terapia Intensiva* (SATI), and *Sociedad Chilena de Medicina Intensiva* (SOCHIMI) also supported this survey by promoting this study on their websites.

This survey was an open, voluntary and anonymous for which participants and researchers did not receive any incentives to participate. It was hosted on the SurveyMonkey website (www.surveymonkey.com) (San Mateo, CA). The first distribution (before pandemic) occurred between September 7th, 2019 and January 7th, 2020 and the second one (during pandemic) took place between January 13th, 2021 and February 15th, 2021. The questionnaires in which only the first part was answered, concerning the ICU profiles and professional expertise, were excluded. All remaining questionnaires regardless of completeness were included in the final analysis as the number of responses for each question was not constant. The computer Internet Protocol (IP) address of respondents was recorded, and answers from the same IP address were carefully checked before statistical analyses were done. If duplicate entries were noted, these entries were excluded. Cookies and other log file analyses were not used to identify a respondent. A Checklist for Reporting Results of Internet E-Surveys (CHERRIES) [19] was used to report the data (Additional file 9).

The survey did not contain data that could identify respondents. The Institutional Review Board of the *Universidade do Extremo Sul Catarinense*, Santa Catarina, Brazil (the main institution for the study) approved the study (ID 3.542.658). Written informed consents containing the purpose of the study, survey duration, and researchers’ identification were obtained from all the participants on the first page of the questionaries. All respondents who completed the questionnaire agreed to participate. All study steps were conducted in compliance with the Declaration of Helsinki [20].

### Statistical analyses

Survey results were exported to a Microsoft Excel template (Microsoft, Redmond, WA) and were analyzed using R software (R Foundation, Vienna, Austria), version 3.6.1 through the following packages: ‘ggplot2’, ‘questionr’, ‘dplyr’, ‘likert’, ‘gridExtra’, ‘pacman’, ‘psych’, ‘car’, ‘MASS’, ‘DescTools’, ‘QuantPsyc’ and ‘scales’. A map showing the worldwide distribution of respondents was produced on Microsoft Excel version 16.42 (Microsoft). Aggregated responses were reported as frequencies and percentages. Continuous data were reported as means and standard deviations or medians and interquartile ranges, according to normality. Stratified analysis by continent was defined a priori. However, continents with < 30 respondents were excluded from this analysis. Univariate and logistic regression were performed and aimed to identify characteristics of respondents and ICUs, independently associated with certain practices, such as monitoring pain and delirium, the use of non-pharmacological treatments for pain, the non-routine use of mechanical restraints for patients under MV, and the use of structured tools to investigate pain, sedation levels and delirium. The variables in each explored practice were included in the logistic regression model if they presented a *p* < 0.2 in respective univariate analysis. A Chi-squared test was used to compare categorical variables. For continuous variables, the Student’s t-test or the Mann–Whitney test were used when appropriate. A two-sided *p*-value < 0.05 was considered significant. No statistical adjustments, such as weighing items or propensity scores, were used.

## Results

A total of 2122 questionnaires was received with 1762 before and 360 during the COVID-19 pandemic. We excluded 354 questionnaires (in 331, only the first part was answered, and in 23, respondents were pediatricians). Most questionnaires were complete (1539 out of 1768, 87%). Respondents were from Europe (*n* = 554, 31.3%), Latin America (*n* = 968, 54.8%), North America (*n* = 29, 1.6%), Asia (*n* = 180, 10.2%), Africa (*n* = 27, 1.5%) and Oceania (*n* = 10, 0.6%). The most represented countries were Brazil (*n* = 643, 36.4%), France (*n* = 353, 20%), and India (*n* = 174, 9.8%) (Additional file 10: Fig. S1). Respondent’s characteristics are described in Table 1.

**Table 1:** Survey respondent demographics and their main practical setting characteristics

Variables	Measures before the COVID-19 pandemic	Measures during the COVID-19 pandemic
Age	41.86 (22.2)^a^	41.9 (10)^a^
Years of practice	12.5 (5–19)^b^	9 (5–20)^b^
Critical care medicine specialist—*n* (%)	1126 (76.3)	216 (74.0)
Years of specialty in critical care medicine	8 (3–17)^b^	9 (4–11)^b^

	*n* (%)	*n* (%)
Main practical setting—type of hospital		
Public hospital	563 (38.2)	115 (39.6)
Private hospital	462 (31.3)	77 (26.6)
University hospital/teaching hospital	449 (30.5)	98 (33.8)
Total	1474 (100.0)	290 (100.0)
Main practical setting—type of ICU^c^		**–**
Mixed ICU	982 (66.7)	**–**
Medical	228 (15.5)	**–**
Surgical	148 (10.0)	**–**
Other	115 (7.8)	
Total	1473 (100.0)	
Number of ICU^c^ beds		**–**
Up to 10	520 (35.3)	**–**
11–20	593 (40.2)	**–**
> 20	361 (24.5)	
Total	1474 (100.0)	
Estimated % of patients under mechanical ventilation^d^		
< 20%	183 (12.4)	24 (8.2)
20–40%	534 (36.2)	59 (20.3)
41–70%	535 (36.3)	136 (46.7)
> 70%	223 (15.1)	72 (24.7)
Total	1475 (100.0)	291 (100.0)

Nursing patient ratio^d^	Daytime—*n* (%)/Nighttime—*n* (%)	Daytime—*n* (%)/Nighttime—*n* (%)
1:1	60 (4.1)/44 (3.0)	18 (6.2)/15 (5.2)
1:2	690 (46.8)/588 (39.9)	106 (36.4)/84 (28.9)
1:3	448 (30.4)/497 (33.7)	78 (26.8)/91 (31.3)
1:4	100 (6.8)/128 (8.7)	31 (10.6)/41 (14.1)
1:5	79 (5.4)/73 (4.9)	27 (9.3)/25 (8.6)
> 1:5	91 (6.2)/132 (8.9)	27 (9.3)/32 (11.0)
Non-applicable	7 (0.3)/13 (0.9)	4 (1.4)/3 (0.9)
Total	1475 (100.0)/1475 (100.0)	291 (100.0)/291 (100.0)

Existence of daily multidisciplinary rounds in the ICU	*n* (%)	*n* (%)
	1363 (92.4)	267 (91.7)
Professionals participating in multidisciplinary rounds		
Doctor	1463 (99.1)	287 (98.3)
Nurse	1308 (88,6)	261 (89.4)
Physical therapist	1045 (70.8)	223 (76.4)
Nutritionist	625 (42.3)	109 (37.3)
Pharmacist	463 (31.4)	93 (31.8)

^a^Mean (standard deviation); ^b^median (interquartile ratio); ^c^ICU, intensive care unit; ^d^*p* < 0.001

### Practices before the COVID-19 pandemic

#### Pain

**Fig. 1:**
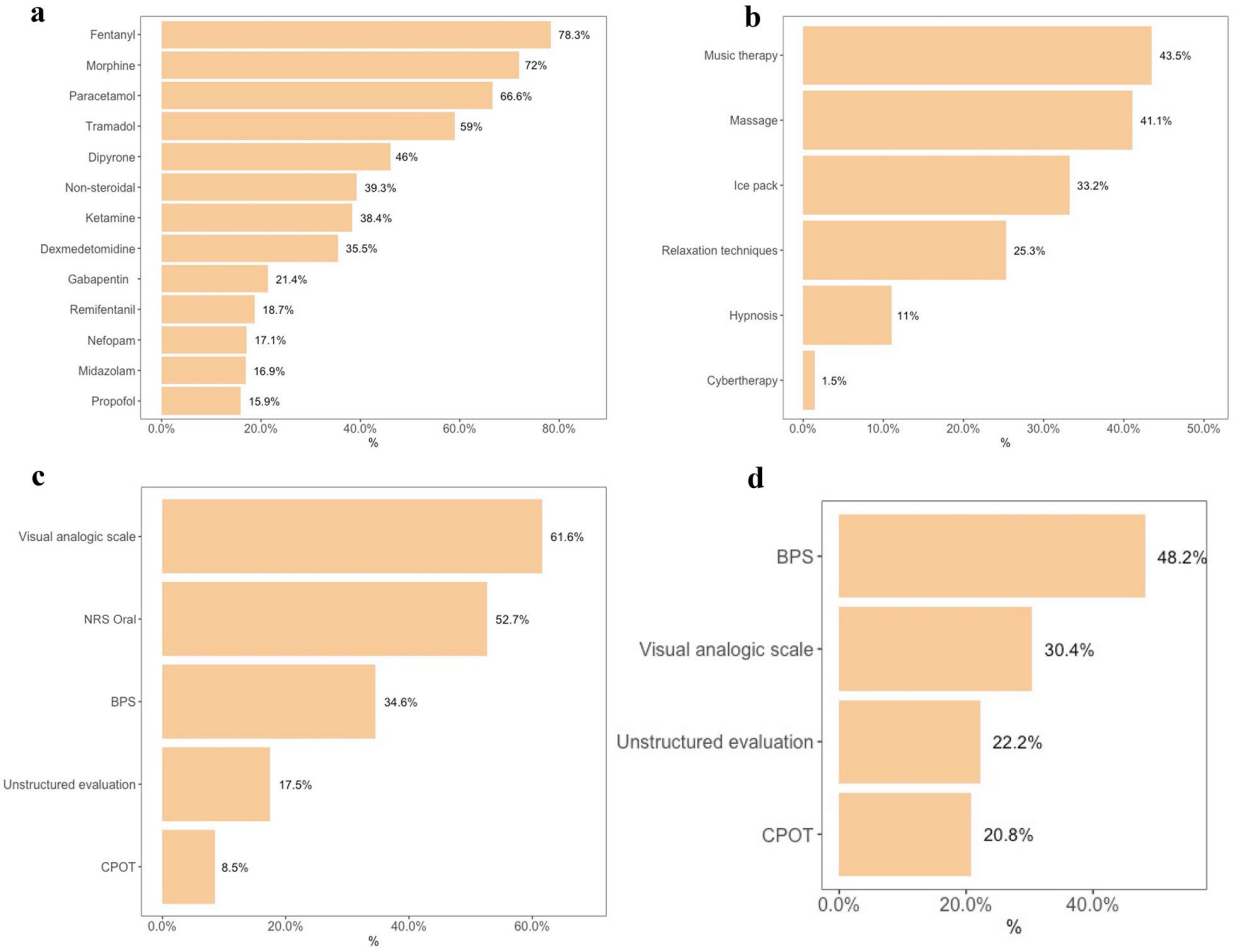
Analgesia practices before the COVID-19 pandemic. **a** Drugs for pain management; **b** nonpharmacologic strategies for pain management; **c** tools for pain assessment in patients able to communicate; **d** tools for pain assessment in patients unable to communicate

Most physicians (86.7%) assessed pain in patients who were able to communicate, being the Visual Analog Scale (VAS) and the numeric rating scale oral (NRS Oral) the most used scales (Fig. 1). However, only 67.4% of the physicians assessed pain in patients who were unable to communicate and the Behavioral Pain Score (BPS) was the main tool used for these patients (Fig. 1). Intravenous fentanyl was the primary drug for pain management, followed by morphine (Fig. 1); however, this finding varied by continent (Additional file 10: Table S1). Among non-opioid medications, paracetamol was the most often used analgesic (66.6%) in all continents, except South America, where dipyrone was the primary drug of choice (75.7%) (Additional file 10: Table S1). Drugs for analgesia are represented in Fig. 1. Forty-one percent of the participants reported nonpharmacologic strategies for pain management, and the participation of physiotherapists in daily rounds was the only factor independently associated with this management (odds ratio [OR] 1.54; confidence interval [CI] 1.14–2.09; *p* = 0.001) (Additional file 10: Table S2). Music therapy was the most common non-pharmacological intervention for pain (Fig. 1). Most respondents reported having daily rounds with intensive care specialists (92.4%) and an analgesia protocol in their ICU (66.8%). These characteristics were independently associated with pain monitoring using structured tools in patients who were able or not able to communicate (Additional file 10: Tables S3–S8).

### Agitation and sedation

**Fig. 2:**
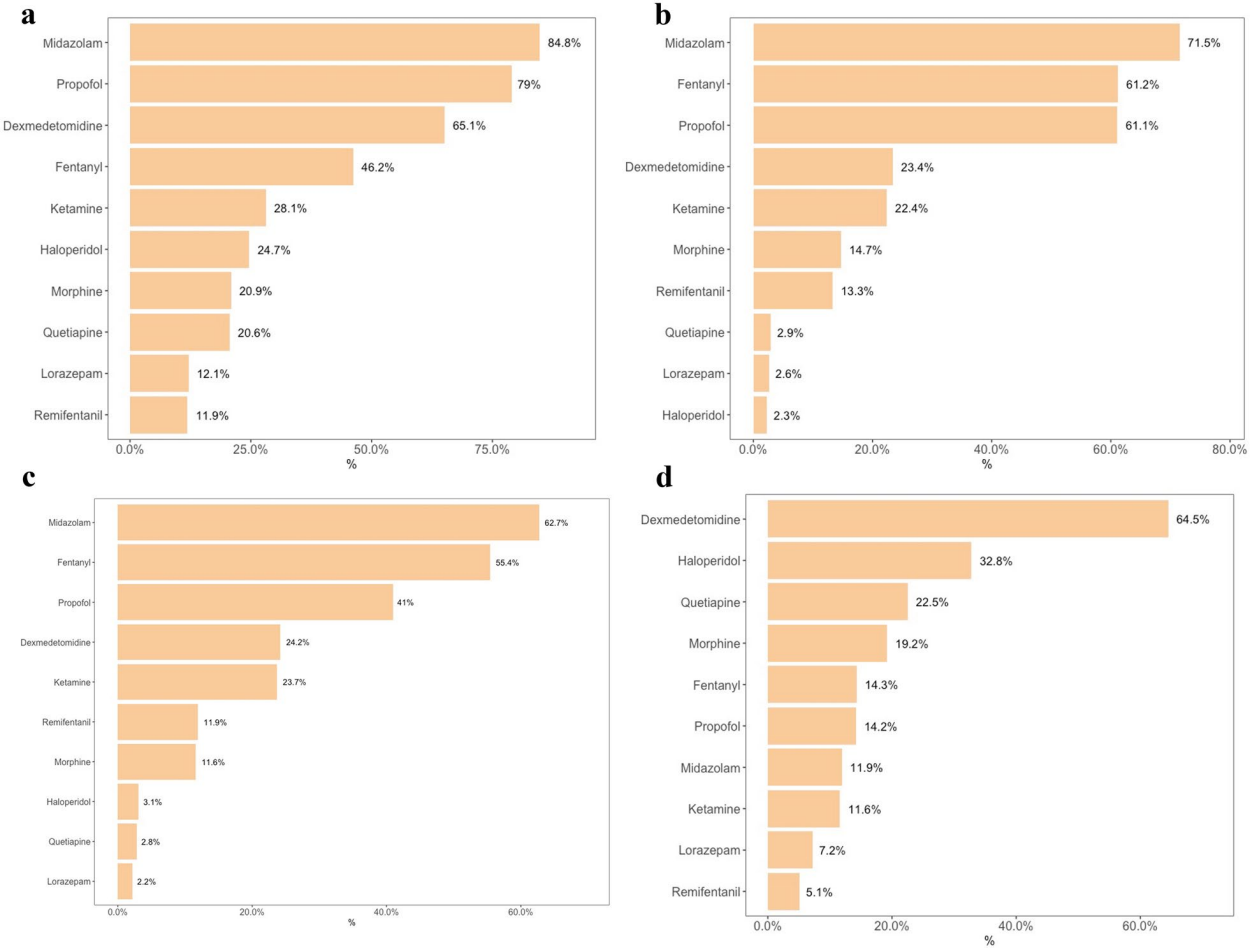
Sedation practices in different clinical settings before the COVID-19 pandemic. **a** Drugs usually used for sedation in patients under MV; **b** drugs used for sedation in patients with moderate-to-severe acute respiratory distress syndrome (ARDS) [25]; **c** drugs used for sedation in patients with septic shock; **d** drugs used for sedation in patients under noninvasive MV

**Fig. 3:**
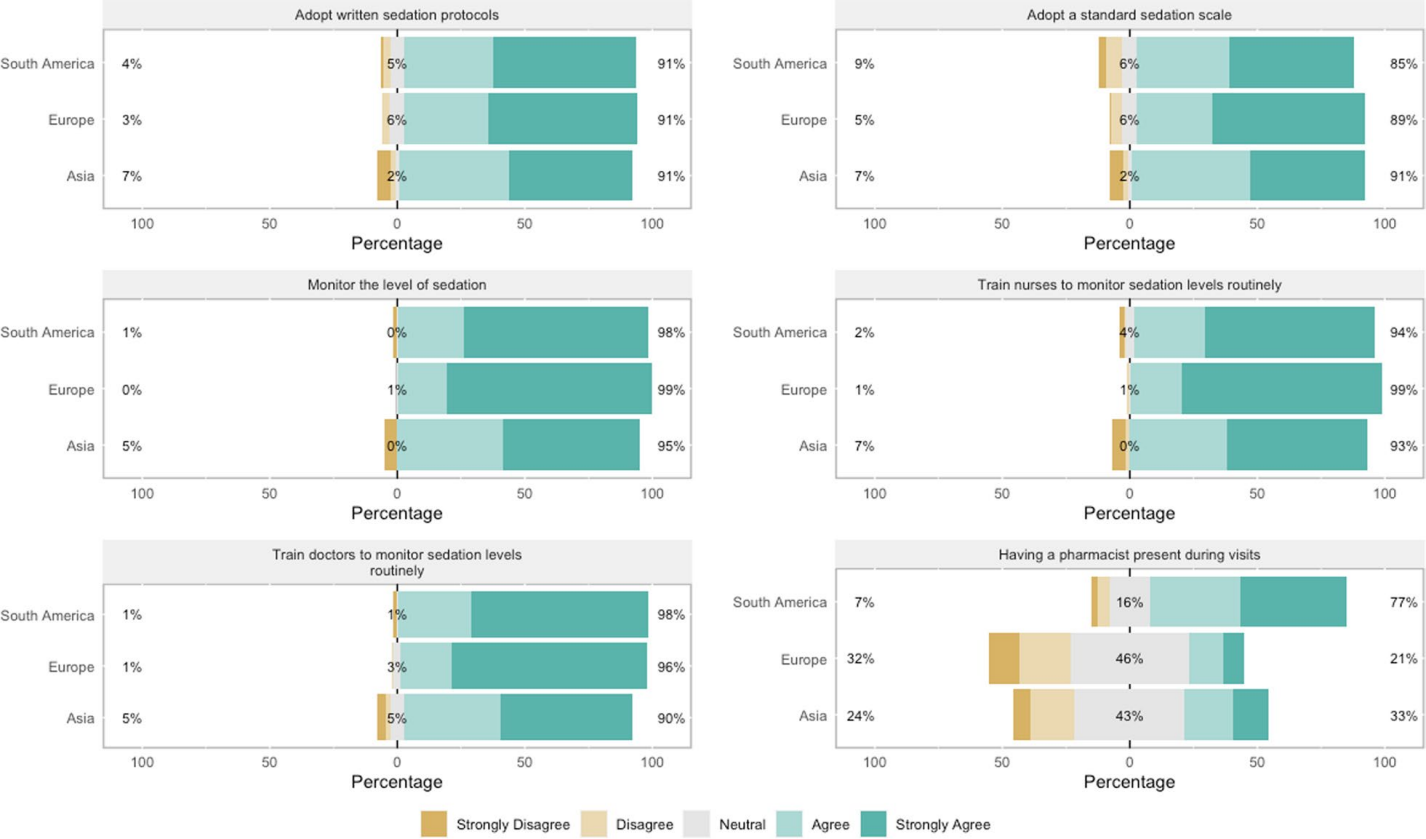
Physicians’ opinions about strategies for improving ICU sedation practices stratified by continents

Most physicians utilized a sedation scale for sedation level evaluation (85.4%). The Richmond Agitation–Sedation Scale (RASS) was most widely used (76.6%) as shown in Additional file 10: Fig. S2. Univariable analysis and factors independently associated with sedation scale use are shown in Additional file 10: Tables S9 and 10. Midazolam was the most used sedative (84.8%) followed by propofol (79%). However, only 41% of the physicians prescribed propofol for patients who were in septic shock (Fig. 2). The sedation drugs use frequency in specific clinical scenarios is represented in Fig. 2. Heterogeneity for sedation drug use among continents was observed (Table 2). Only 56% of physicians avoided some sort of sedatives, and midazolam was the most frequently avoided one (41.9%), as shown in Additional file 10: Fig. S3. Most physicians routinely used sedation for patients requiring MV (86%) and continuous sedation with dose titration was the main strategy (54.9%); however, variability in sedation strategies among the continents was found (Additional file 10: Table S11). Most respondents reported a sedation protocol in their practical settings (69.2%), and 65.5% always followed it. Many physicians (76.2%) reported that sedation goals were discussed during daily rounds, and most of them checked patient sedation level at least three times a day (58.2%). When asked about strategies for improving ICU sedation practices, most respondents agreed or strongly agreed with several practices: (1) adopting a sedation protocol (91%); (2) adopting a standard sedation scale (88%); (3) monitoring the sedation level (98%), and (4) training nurses and doctors to monitor the sedation level regularly (96% and 95%, respectively). Only half of the participants believe that the presence of a pharmacist in daily rounds is a valid strategy for improving sedation practices (Additional file 10: Fig. S4). This percentage increased to 70% among physicians reporting a pharmacist did participate in daily rounds (Additional file 10: Fig. S5). The physicians’ opinions about strategies for improving ICU sedation practices stratified by continent are represented in Fig. 3. Only 19% of the participants ordered mechanical restraints for patients on MV as part of the treatment routine (Additional file 10: Fig. S6). A rate of nurse per patient of (1:1 and 1:2) during daytime was the only variable independently associated with non-routine use of mechanical restraints (Additional file 10: Tables S12 and 13).

**Table 2:** Sedation choices by continent before the COVID-19 pandemic

Variables *n*	Asia 169 (%)	Europe 408 (%)	South America 739 (%)
Drugs usually used for sedation			
*Midazolam*^*a*^	*65.1*	*80.9*	*92.3*
Lorazepam^a^	10.1	4.4	16.6
Haloperidol^b^	20.7	20.3	28.3
Morphine^a^	45.6	17.2	17.6
Fentanyl^a^	77.5	19.1	53.6
* Propofol*^*a*^	*50.9*	*91.9*	*78.1*
Remifentanil^a^	0.6	27.9	6.1
Dexmedetomidine^a^	64.5	61.00	67.9
Ketamine^a^	5.9	25.7	34.2
Quetiapine^a^	14.2	8.3	29.2
Sedative drugs used for sedation in patients with septic shock			
*Midazolam*^*a*^	*34.9*	*64.6*	*69.1*
Lorazepam	3	0.7	2.7
Haloperidol^b^	4.1	0.7	3.9
Morphine^a^	32.5	11.8	7
Fentanyl^a^	79.9	25	66.1
*Propofol*^*a*^	*6.5*	*46.6*	*44.7*
Remifentanil^a^	0.59	20.3	10.2
Dexmedetomidine^a^	20.1	12	30.9
Ketamine^a^	8.3	16.2	32
Quetiapine^b^	2.4	0.3	4.2
Sedative drugs used for sedation in patients with ARDS			
*Midazolam*^a^	*49.7*	*66.7*	*80.4*
Lorazepam^b^	0.6	0.3	4
Haloperidol	2.4	1	3
Morphine^a^	39	14.5	10
*Fentanyl*^a^	*82.3*	*29.9*	*73*
Propofol^a^	22.5	67.2	65.6
Remifentanil^a^	0	21.6	10.26
Dexmedetomidine^a^	35.5	11.8	26.1
Ketamine^a^	2.4	16.4	30.9
Quetiapine	3.6	0.5	3.9
Sedative drugs used for sedation in agitated patients under noninvasive ventilation			
Midazolam^b^	20.1	13.7	8.9
Lorazepam	5.3	5.9	8.1
*Haloperidol*^b^	*23.7*	*35.3*	*34*
Morphine^b^	13.6	14.5	23.7
Fentanyl^a^	32	6.1	14.9
Propofol^a^	3	25.5	10.8
Remifentanil^a^	1.8	9.6	3.7
*Dexmedetomidine*^a^	*38.5*	*55.9*	*75.5*
Ketamine^a^	0.6	7.4	16.6
Quetiapine^a^	13	10.3	31.7

^a^ *p* < 0.001; ^b^*p* < 0.05; ^c^ARDS, acute respiratory distress syndrome

Italic emphasis are the most frequent choice in each context

### Delirium

Most physicians evaluated the presence of delirium at least once per day (95.4%), but only for patients presenting with clinical suspicion (61.5%). The most common diagnostic method was the Confusion Assessment Method for the Intensive Care Unit ([CAM-ICU] 66.6%), followed by a general clinical evaluation (52.1%) (Additional file 10: Fig. S7). Haloperidol (68.8%) and atypical antipsychotics (69.4%) were the most frequently prescribed drugs for delirium (Additional file 10: Fig. S8). However, when asked specifically about the hypoactive phenotype, most physicians reported using nonpharmacologic treatment strategies (54.3%) as described in Additional file 10: Figs. S9 and S10. Most physicians did not know the prevalence of delirium in their units (59.3%), but among those who knew, the prevalence was between 10 and 25%. The knowledge about delirium frequency, the presence of a physiotherapist in multidisciplinary rounds and teaching/university hospitals were factors independently associated with delirium investigation, using structured tools (Additional file 10: Tables S14–S17).

### Mobilization

Early mobilization was performed by 96.5% of the respondents and just 16.8% of them mobilized only patients without MV support. A mobilization team was present in 38.8% of the ICUs and the most common technique was verticalization by staff (at bedside, standing up, sitting on a chair, and walking) (93%), followed by the cycle ergometer (35%) as shown in Additional file 10: Fig. S11.

### Sleep deprivation

Very few physicians regularly prescribed drugs to induce sleep in patients requiring MV (13%) and 19% of them did not (Additional file 10: Fig. S6). Benzodiazepines (including midazolam), dexmedetomidine, and propofol were drugs for treating sleep deprivation (Additional file 10: Fig. S12).

Nonpharmacological strategies were used by 59.3% of the respondents, such as light (98%) and noise (89.4%) reduction and avoiding sleep disruption by procedures (such as exams, baths, medications, etc.) (81.4%) (Additional file 10: Fig. S13).

### Practices during the COVID-19 pandemic

Most practices about sedation, analgesia and delirium during the COVID-19 pandemic were comparable to the pre-COVID period. The rate of patients on MV was higher in the former period (Table 1). As expected, they received sedation more often than before pandemic (94% versus 86.1%, *p* ≤ 0.001) and their sedation goals were more frequently discussed in daily rounds (Additional file 10: Table S18). Morphine, instead of fentanyl, was the main analgesic used (77.2% versus 69%) as described in Additional file 10: Table S19. Some sedative drugs were prescribed more frequently during this period. These drugs included midazolam (84.7% versus 91.4%, *p* < 0.05), propofol (79% versus 88.4%, *p* ≤ 0.001), ketamine (28.1% versus 38.6%, *p* ≤ 0.001) and quetiapine (20.6% versus 27.3%, *p* < 0.05) as shown in Additional file 10: Table S18. Practices and management of delirium were similar (Additional file 10: Table S20). Being an intensive care specialist was independently associated with many practices, such as non-pharmacological treatment for pain, non-routine use of mechanical restraints, and sedation levels and pain investigation, using structured tools (Additional file 10: Tables S21–S32). As before the COVID-19 pandemic, knowledge about delirium frequency was associated with delirium investigation using structured tools (Additional file 10: Tables S33–S36).

## Discussion

This is a multinational survey directed to physicians aimed at learning the current practices in sedation, analgesia, delirium management, mobilization, and sleep disruption. We observed a frequent use of validated tools to assess pain, sedation levels, and delirium. Moreover, early mobilization and non-pharmacological strategies for pain management, hypoactive delirium, and sleep deprivation were implemented. These findings are aligned with current recommended practices [14]. Midazolam, fentanyl, and haloperidol or atypical antipsychotics remain as the main drugs selected for sedation, pain, and delirium management, respectively [10, 15, 21–23]. This study provides information about sedation choices in specific situations such as moderate and severe ARDS, septic shock, and noninvasive MV. It also highlights that the COVID-19 pandemic has led to changes in some of those practices.

Most physicians reported monitoring sedation with validated scales [15, 24], which is in accordance with clinical guidelines [14]. We observed that the preferred scale changed from the Ramsay sedation scale in the 2000s [22] to the RASS scale more recently [15, 24–26], a finding reinforced by our study. The rate of physicians monitoring delirium has increased over time; it was only 61.6% in 2009 [21], 70% in 2017 [15], and 84.5% in our survey. Validated tools to assess delirium were frequent, but nonstructured evaluation also turned out to be frequent in the present study; their low accuracy can lead to delay and disagreement towards delirium diagnosis and underestimation of its prevalence [27], and it is not recommended [14]. As in the Morandi et al. survey [15], we showed that early mobilization was regularly prescribed. Another positive finding was the rare use of mechanical restraints as part of routine care for mechanically ventilated patients. No randomized trials evaluating this practice are available, but studies showed that physical restraint is associated with the development of delirium and post-traumatic stress disorder [28, 29].

Pain is a frequent and distressful symptom for patients in the ICU [3, 30]. In our survey, pain was assessed less often in patients unable to verbalize, which can be improved by training the teams to properly use and evaluate the pain scales. Opioids were routinely prescribed even out of the context of pain management, such as for sedation and treatment of sleep deprivation, which can raise awareness of the dangers its excessive use may cause [31, 32].

Midazolam is still used often despite guidelines that advise propofol over benzodiazepines [14, 33]. We observed that many physicians do not use propofol for sedation in patients with septic shock, but there is no recommendation to support this practice [14, 34].

In the present survey, a minority of respondents believe that the presence of a pharmacist during daily rounds could lead to improvement of sedation practices, which resembles the Brazilian survey about delirium recognition and sedation practices in critically ill patients [21]. However, numerous studies showed the benefits of a having pharmacist participate in pain management, agitation, and delirium [35–40]. Moreover, an ICU pharmacist is associated with reductions in mortality, length of stay in hospital and ICU and also in adverse drug events caused by prescribing errors [41–43].

The use of midazolam, dexmedetomidine, and propofol to induce sleep in patients on MV was often seen in our study, but this practice is not in accordance with evidence-based recommendations [14]. Safety and efficacy of sedative drugs to improve sleep in the ICU were scarcely tested, which may increase the risk of polypharmacy and delirium rather than promoting sleep [14]. A study by Oto et al. showed that dexmedetomidine induced disturbances in sleep architecture [44].

The prescription of sedatives for patients on MV increased during the COVID-19 pandemic, possibly due to the high prevalence of ARDS [45, 46]. Similar to this survey, a cohort study found a high sedation rate during MV, with midazolam and propofol as the most used sedatives [46]. Most sites in this cohort also used RASS for sedation and CAM-ICU for delirium assessments [46]. Medication shortage, expected longer duration of MV and the presence of non-ICU trained staff to treat COVID-19 patients during the pandemic could explain the use of a greater variety of sedation and analgesic drugs in the second phase of this survey [47, 48].

Our study has several strengths. This is a large survey about all themes in the latest PADIS guideline for prevention and management of pain, agitation/sedation, delirium, immobility, and sleep disruption in ICU adult patients [14]. The presence of duplicated answers was checked and not found. The questionnaire was available in four languages and answered by physicians from all continents. However, some limitations also exist: (1) a questionnaire reliability assessment was not carried out; (2) the number of respondents during the COVID-19 pandemic was lower than before the pandemic, which can be justified by physicians being overwhelmed during that period; (3) the response rate is unknown and open web surveys are associated with an inherent selection bias; (4) the stratified analysis of the practices by continents was limited due to selection bias, and (5) most of the respondents of this survey were from Brazil, France and India. Moreover, it is known that the adherence rate to preconized practices in surveys consistently exceeds the actual findings [49, 50]; therefore, the data represent perceived practices rather than implemented interventions.

## Conclusion

Most sedation, analgesia and delirium practices were comparable during the COVID-19 pandemic. During that period, the intensive care specialty was associated with the best practices. Variability in practices by country was found, but it is impossible to assume continent comparability with these data. Although many findings are in accordance with evidence-based recommendations, some practices still need improving, for instance, pain assessment in patients unable to communicate, nonstructured evaluation for delirium assessment, infrequent presence of a pharmacist during daily ICU rounds, inappropriate use of sedative drugs to promote sleep, and the use of opioids out of the context of pain management. Promoting best practices and educational strategies are necessary to achieve these advances.

## Supplementary Information

**Additional file 1:** English version of the questionnaire. Contains English version of the questionnaire administrated before the COVID-19 pandemic.

**Additional file 2:** Spanish version of questionnaire. Contains Spanish version of the questionnaire administrated before the COVID-19 pandemic.

**Additional file 3:** French version of questionnaire. Contains French version of the questionnaire administrated before the COVID-19 pandemic.

**Additional file 4:** Portuguese version of the questionnaire. Contains the Portuguese version of the questionnaire administrated before the COVID-19 pandemic.

**Additional file 5:** English version of the questionnaire—COVID-19. Contains English version of the questionnaire administrated during the COVID-19 pandemic.

**Additional file 6:** Spanish version of the questionnaire—COVID-19. Contains Spanish version of the questionnaire administrated during the COVID-19 pandemic.

**Additional file 7:** French version of the questionnaire—COVID-19. Contains French version of the questionnaire administrated during the COVID-19 pandemic.

**Additional file 8:** Portuguese version of the questionnaire—COVID-19. Contains Portuguese version of the questionnaire administrated during the COVID-19 pandemic.

**Additional file 9:** CHERRIES Checklist. Contains checklist for Reporting Results of Internet E-Surveys (CHERRIES).

**Additional file 10:** Supplementary Material. Contains the supplementary results of the study.

## Data Availability

The datasets used and/or analyzed during the current study are available from the corresponding author on reasonable request.

## Abbreviations

ARDS: Acute respiratory distress syndrome
BPS: Behavioral Pain Score
CAM-ICU: Confusion Assessment Method for the Intensive Care Unit
CHERRIES: Checklist for Reporting Results of Internet E-Surveys
COVID-19: Coronavirus 2019 disease
ICU: Intensive Care Unit
IP: Internet Protocol
MV: Mechanical ventilation
PADIS: 2018 Clinical Practice Guidelines for the Prevention and Management of Pain, Agitation/Sedation, Delirium, Immobility, and Sleep Disruption in Adult Patients in the ICU
RASS: Richmond Agitation–Sedation Scale
VAS: Visual Analogic Scale
