# Supplementary material for: Practices in sedation, analgesia, mobilization, delirium, and sleep deprivation in adult intensive care units (SAMDS-ICU): an international survey before and during the COVID-19 pandemic
Source: Ann Intensive Care. 2022 Feb 4;12:9. doi: 10.1186/s13613-022-00985-y (PMC8815719; doi:10.1186/s13613-022-00985-y)
Supplement: Supplementary file 1 — Additional file 1: English version of the questionnaire. Contains English version of the questionnaire administrated before the COVID-19 pandemic. [file 13613_2022_985_MOESM1_ESM.pdf]

Sedation, Analgesia, Mobilization, Delirium and sleep deprivation in ICU - Multi-center and international study - SAMDS Study

Informed Consent

We would like to invite you to participate of this survey on practices of sedation, analgesia, delirium management and sleep deprivation in the intensive care unit. This study will be performed with an self-applied questionnaire (8 minutes duration), about your practice of sedation, analgesia, mobilization, sleep improvement, as well as delirium screening, monitoring and treatment in your work environment.

The researchers did not receive any financial support for doing thins study, and you will not have financial compensation for participating of this research. You will not be identified in this questionnaire. If you agree in participating of this study, please click on the dialog box below to have access to the questionnaire.

Doubts can be cleared at any time with the steering committee members.

Steering Committee of the SAMDS study:

Bruna Brandão Barreto (brunab_barreto@yahoo.com.br) - Brazil
Mariana Luz (marianaluzmed@gmail.com) - Brazil
Eduardo Tobar (edotobar@gmail.com) - Chile
Audrey De Jong (audreydejong@hotmail.fr) - France
Gérald Chanques (g-chanques@chu-montpellier.fr) - France
John Kress (jkress@medicine.bsd.uchicago.edu) - USA
Yahya Shehabi (yshehabi@ozmail.com.au) - Australia/New Zealand
Roberta Esteves Vieira de Castro (roberta-esteves@hotmail.com) - Brazil
Jorge Salluh (jorgesalluh@gmail.com) - Brazil
Felipe Dal-Pizzol (fdpizzol@gmail.com) - Brazil
Dimitri Gusmao-Flores (dimitrigusmao@gmail.com) - Brazil

* 1. Do you want to participate?

☐ Yes

Sedation, Analgesia, Mobilization, Delirium and sleep deprivation in ICU - Multi-center and international study - SAMDS Study

* 2. In which country do you work?

* 3. Age (completed years):

* 4. How long have you been working in intensive care (completed years)?

* 5. Are you an intensive care specialist?

- Yes
- No

SAMDS
SEDATION, ANALGESIA, MOBILIZATION, DELIRIUM AND SLEEP DEPRIVATION IN ICU

Sedation, Analgesia, Mobilization, Delirium and sleep deprivation in ICU - Multi-center and international study - SAMDS Study

* 6. How long have you been an intensive care specialist (completed years)?

SAMDS
SEDATION, ANALGESIA, MOBILIZATION, DELIRIUM AND SLEEP DEPRIVATION IN ICU

The answers below refer to the place where you dedicate the most of your working hours:

* 7. Type of hospital:

- Public hospital
- University Hospital / Teaching hospital
- Private Hospital

* 8. Type of ICU:

- Medical
- Cardiac
- Surgical
- Mixed
- Neuro
- Trauma
- Other (please specify)

9. Number of beds in your ICU:

- Up to 10
- 11-20
- >20

10. What is the frequency of patients using mechanical ventilation in your ICU?

- <20%
- 20-40%
- 40-70%
- >70%

11. Nursing : patient ratio (daytime):

- 1:1
- 1:2
- 1:3
- 1:4
- 1:5
- >1:5
- Not apply

12. Nursing : patient ratio (nighttime):

- ◯ 1:1
- ◯ 1:2
- ◯ 1:3
- ◯ 1:4
- ◯ 1:5
- ◯ >1:5
- ◯ Not apply

13. Has your ICU been organized with daily rounds with an intensive care specialist?

- ◯ Yes
- ◯ No

14. Which professional participate on multidisciplinary rounds (check all that apply)?

- ☐ Doctor
- ☐ Nurse
- ☐ Physiotherapist
- ☐ Nutritionist
- ☐ Pharmacist

15. Is there any analgesia protocol in your ICU?

- ◯ Yes
- ◯ No
- ◯ I don’t know

16. Do you monitor pain in patients that are able to communicate?

- ◯ Yes
- ◯ No

SAMDS
SEDATION, ANALGESIA, MOBILIZATION, DELIRIUM AND SLEEP DEPRIVATION IN ICU

17. How do you monitor pain in these patients (check all that apply)?

- [ ] Visual analogic scale
- [ ] Behavioral Pain Scale – BPS and/or BPS for non intubated patients
- [ ] Critical-Care Pain Observation Tool - CPOT
- [ ] "Other (please specify)"
- [ ] Unstructured evaluation
- [ ] Oral numeric scale

SAMDS
SEDATION, ANALGESIA, MOBILIZATION, DELIRIUM AND SLEEP DEPRIVATION IN ICU

Sedation, Analgesia, Mobilization, Delirium and sleep deprivation in ICU - Multi-center and international study - SAMDS Study

18. Do you monitor pain in patients that are not able to communicate?

- ( ) Yes
- ( ) No

SAMDS
SEDATION, ANALGESIA, MOBILIZATION, DELIRIUM AND SLEEP DEPRIVATION IN ICU

Sedation, Analgesia, Mobilization, Delirium and sleep deprivation in ICU - Multi-center and international study - SAMDS Study

19. How do you monitor pain in these patients (check all that apply)?

- [ ] Visual analogic scale
- [ ] Unstructured valuation
- [ ] Behavioral Pain Scale – BPS and/or BPS for non intubated patients
- [ ] Oral numeric scale
- [ ] Critical-Care Pain Observation Tool - CPOT
- [ ] "Other (please specify)"

SAMDS

SEDATION, ANALGESIA, MOBILIZATION, DELIRIUM AND SLEEP DEPRIVATION IN ICU

Sedation, Analgesia, Mobilization, Delirium and sleep deprivation in ICU - Multi-center and international study - SAMDS Study

20. Which drugs do you usually use for analgesia (check all that apply)?

- [ ] Midazolam
- [ ] Propofol
- [ ] Dipyrone (metamizole)
- [ ] Dexmedetomidine
- [ ] Morphine
- [ ] Nonsteroidal anti-inflammatory drugs
- [ ] Fentanyl
- [ ] Paracetamol
- [ ] Remifentanil
- [ ] Nefopam
- [ ] Tramadol
- [ ] Ketamine
- [ ] Gabapentine
- [ ] "Other (please specify)"

21. Do you use non pharmacologic therapy to treat pain?

- ( ) Yes
- ( ) No

SAMDS
SEDATION, ANALGESIA, MOBILIZATION, DELIRIUM AND SLEEP DEPRIVATION IN ICU

Sedation, Analgesia, Mobilization, Delirium and sleep deprivation in ICU - Multi-center and international study - SAMDS Study

22. Which one (check all that apply)?

- ☐ Massage
- ☐ Hypnosis
- ☐ Cybertherapy
- ☐ Relaxation techniques
- ☐ Cold therapy
- ☐ Music therapy
- ☐ Other (please specify)

SAMDS
SEDATION, ANALGESIA, MOBILIZATION, DELIRIUM AND SLEEP DEPRIVATION IN ICU

Sedation, Analgesia, Mobilization, Delirium and sleep deprivation in ICU - Multi-center and international study - SAMDS Study

23. Is there any sedation protocol in your ICU?

- ○ Yes
- ○ No
- ○ I don’t know

SAMDS
SEDATION, ANALGESIA, MOBILIZATION, DELIRIUM AND SLEEP DEPRIVATION IN ICU

Sedation, Analgesia, Mobilization, Delirium and sleep deprivation in ICU - Multi-center and international study - SAMDS Study

24. How often do you follow the sedation protocol?

- ◯ Never
- ◯ Sometimes
- ◯ Always

SAMDS
SEDATION, ANALGESIA, MOBILIZATION, DELIRIUM AND SLEEP DEPRIVATION IN ICU

Sedation, Analgesia, Mobilization, Delirium and sleep deprivation in ICU - Multi-center and international study - SAMDS Study

25. In your unit, do you routinely use sedative drugs for patients on mechanical ventilation?

- ◯ Yes
- ◯ No

26. When using sedative drugs for patients on mechanical ventilation, what is the most frequently used strategy?

- ◯ Continuous sedation with titration
- ◯ Continuous sedation with daily interruption
- ◯ Intermittent bolus

27. How often are the sedation goals discussed during rounds?

- ◯ Daily
- ◯ Sporadically
- ◯ Never

28. Do you use any sedation scale routinely?

- ○ Yes
- ○ No

SAMDS

SEDATION, ANALGESIA, MOBILIZATION, DELIRIUM AND SLEEP DEPRIVATION IN ICU

Sedation, Analgesia, Mobilization, Delirium and sleep deprivation in ICU - Multi-center and international study - SAMDS Study

29. Which scale do you use (check all that apply)?

- ☐ Ramsay
- ☐ Sedation-agitation scale - SAS
- ☐ Richmond agitation-sedation scale - RASS
- ☐ Glasgow
- ☐ "Other (please specify)"

SAMDS

SEDATION, ANALGESIA, MOBILIZATION, DELIRIUM AND SLEEP DEPRIVATION IN ICU

Sedation, Analgesia, Mobilization, Delirium and sleep deprivation in ICU - Multi-center and international study - SAMDS Study

30. How many times a day do you assess the level of sedation of patients in the ICU?

- ○ 1
- ○ 2
- ○ 3
- ○ >3

* 31. To improve sedation practices in ICUs, we must:

	Strongly Disagree	Disagree	Neutral	Agree	Strongly Agree
Adopt written protocols of sedation:	◯	◯	◯	◯	◯
Adopt a standard sedation scale:	◯	◯	◯	◯	◯
Monitor the level of sedation:	◯	◯	◯	◯	◯
Train nurses to monitor the levels of sedation routinely:	◯	◯	◯	◯	◯
Train doctors to monitor the levels of sedation routinely:	◯	◯	◯	◯	◯
Have the presence of a pharmacist on visits:	◯	◯	◯	◯	◯

32. Which drugs do you usually use for sedation (check all that apply)?

- [ ] Midazolam
- [ ] Lorazepan
- [ ] Haloperidol
- [ ] Morphine
- [ ] Fentanyl
- [ ] Propofol
- [ ] Remifentanyl
- [ ] Dexmedetomidine
- [ ] Ketamine
- [ ] Quetiapine
- [ ] "Other (please specify)"

33. Is there any sedative drug you do not use or avoid?

◯ Yes

◯ No

SAMDS

SEDATION, ANALGESIA, MOBILIZATION, DELIRIUM AND SLEEP DEPRIVATION IN ICU

34. Which one (check all that apply)?

- [ ] Midazolam
- [ ] Lorazepan
- [ ] Haloperidol
- [ ] Morphine
- [ ] Fentanyl
- [ ] Propofol
- [ ] Remifentanyl
- [ ] Dexmedetomidine
- [ ] Ketamine
- [ ] Quetiapine
- [ ] "Other (please specify)"

SAMDS
SEDATION, ANALGESIA, MOBILIZATION, DELIRIUM AND SLEEP DEPRIVATION IN ICU

Sedation, Analgesia, Mobilization, Delirium and sleep deprivation in ICU - Multi-center and international study - SAMDS Study

Which drugs would you use for sedation in each scenario below (check all that apply):

35. Septic shock:

- [ ] Midazolam
- [ ] Lorazepan
- [ ] Haloperidol
- [ ] Morphine
- [ ] Fentanyl
- [ ] Propofol
- [ ] Remifentanyl
- [ ] Dexmedetomidine
- [ ] Ketamine
- [ ] Quetiapine
- [ ] I don't use sedation
- [ ] "Other (please specify)"

36. Moderate or severe Acute Respiratory Distress Syndrome (ARDS)

- [ ] Midazolam
- [ ] Lorazepan
- [ ] Haloperidol
- [ ] Morphine
- [ ] Fentanyl
- [ ] Propofol
- [ ] "Other (please specify)"
- [ ] Remifentanyl
- [ ] Dexmedetomidine
- [ ] Ketamine
- [ ] Quetiapine
- [ ] I don't use sedation

37. Agitated patients on **non-invasive mechanical ventilation**:

- [ ] Midazolam
- [ ] Lorazepan
- [ ] Haloperidol
- [ ] Morphine
- [ ] Fentanyl
- [ ] Propofol
- [ ] "Other (please specify)"
- [ ] Remifentanyl
- [ ] Dexmedetomidine
- [ ] Ketamine
- [ ] Quetiapine
- [ ] I don't use sedation

SAMDS

SEDATION, ANALGESIA, MOBILIZATION, DELIRIUM AND SLEEP DEPRIVATION IN ICU

Sedation, Analgesia, Mobilization, Delirium and sleep deprivation in ICU - Multi-center and international study - SAMDS Study

38. How often do you use mechanical restraint in patients on mechanical ventilation?

- ( ) Never
- ( ) Sometimes
- ( ) Always

39. How often do you use any drug to induce sleep in patients on mechanical ventilation?

- Never
- Sometimes
- Always

SAMDS
SEDATION, ANALGESIA, MOBILIZATION, DELIRIUM AND SLEEP DEPRIVATION IN ICU

Sedation, Analgesia, Mobilization, Delirium and sleep deprivation in ICU - Multi-center and international study - SAMDS Study

40. Which one do you usually use?

- Midazolam
- Other benzodiazepines
- Morphine
- Fentanyl
- Propofol
- "Other (please specify)"
- Dexmedetomidine
- Ketamine
- Zolpidem
- Melatonin

SAMDS
SEDATION, ANALGESIA, MOBILIZATION, DELIRIUM AND SLEEP DEPRIVATION IN ICU

Sedation, Analgesia, Mobilization, Delirium and sleep deprivation in ICU - Multi-center and international study - SAMDS Study

41. Do you use any non-pharmacologic therapy to promote sleep in ICU?

- Yes
- No

SAMDS
SEDATION, ANALGESIA, MOBILIZATION, DELIRIUM AND SLEEP DEPRIVATION IN ICU

Sedation, Analgesia, Mobilization, Delirium and sleep deprivation in ICU - Multi-center and international study - SAMDS Study

42. Which one (check all that apply)

- ☐ Ear plugs
- ☐ Light reduction
- ☐ Noise control
- ☐ Other (please specify)
- ☐ Sleeping mask
- ☐ Reduce sleep interruption due to exam, bath and medication etc

SAMDS
SEDATION, ANALGESIA, MOBILIZATION, DELIRIUM AND SLEEP DEPRIVATION IN ICU

Sedation, Analgesia, Mobilization, Delirium and sleep deprivation in ICU - Multi-center and international study - SAMDS Study

43. Do you have information about the frequency of delirium in your unit?

- ○ Yes
- ○ No

SAMDS
SEDATION, ANALGESIA, MOBILIZATION, DELIRIUM AND SLEEP DEPRIVATION IN ICU

Sedation, Analgesia, Mobilization, Delirium and sleep deprivation in ICU - Multi-center and international study - SAMDS Study

44. What is this frequency?

- <10%
- 10-25%
- 25-50%
- 50-75%
- >75%

SAMDS
SEDATION, ANALGESIA, MOBILIZATION, DELIRIUM AND SLEEP DEPRIVATION IN ICU

Sedation, Analgesia, Mobilization, Delirium and sleep deprivation in ICU - Multi-center and international study - SAMDS Study

45. Do you investigate the presence of delirium?

- Yes
- No

SAMDS
SEDATION, ANALGESIA, MOBILIZATION, DELIRIUM AND SLEEP DEPRIVATION IN ICU

Sedation, Analgesia, Mobilization, Delirium and sleep deprivation in ICU - Multi-center and international study - SAMDS Study

46. Who is evaluated?

- All patients?
- Only patients with clinical suspicion

47. How do you diagnose delirium? (check all that apply)

- [ ] General clinical evaluation
- [ ] Confusion Assessment Method for the Intensive Care Delirium – CAM-ICU
- [ ] Delirium rating scale
- [ ] "Other (please specify)"
- [ ] Intensive care delirium screening checklist - ICDSC
- [ ] Mini-mental State Examination - MMSE

48. How many times a day is the presence of delirium assessed in your ICU?

- ( ) 0
- ( ) 1
- ( ) 2
- ( ) 3
- ( ) >3

SAMDS

SEDATION, ANALGESIA, MOBILIZATION, DELIRIUM AND SLEEP DEPRIVATION IN ICU

49.
Which drugs do you generally use to treat delirium? (check all that apply)

- [ ] Midazolam
- [ ] Other benzodiazepines
- [ ] Haloperidol
- [ ] Morphine
- [ ] Fentanyl
- [ ] 'Other (please specify)'
- [ ] Propofol
- [ ] Dexmedetomidine
- [ ] Atypical antipsychotics (olanzapine, quetiapine, clozapine, risperidone)
- [ ] I don't use drugs to treat delirium

50. How do you treat hypoactive delirium (check all that apply) ?

- ◯ Pharmacological therapy
- ◯ Non-pharmacological therapy
- ◯ I do not treat

SAMDS

SEDATION, ANALGESIA, MOBILIZATION, DELIRIUM
AND SLEEP DEPRIVATION IN ICU

Sedation, Analgesia, Mobilization, Delirium and sleep deprivation in ICU - Multi-center and international study - SAMDS Study

51. Which non-pharmacological therapy do you use? (check all that apply)

- ☐ Improve sleep
- ☐ Family engagement
- ☐ Mobilization
- ☐ Music therapy
- ☐ Cognitive stimulation / occupational therapy
- ☐ "Other (please specify)"

____

SAMDS

SEDATION, ANALGESIA, MOBILIZATION, DELIRIUM
AND SLEEP DEPRIVATION IN ICU

Sedation, Analgesia, Mobilization, Delirium and sleep deprivation in ICU - Multi-center and international study - SAMDS Study

52. Do you perform early mobilization in your unit?

- ◯ Yes
- ◯ Only in patients without mechanical ventilation support
- ◯ No

53. Do you have early mobilization team in your unit?

- ○ Yes
- ○ No

54. Which techniques of mobilization do you use ?

- ☐ Verticalization by staff (seating at the bedside, standing up, seating in a chair, walking)
- ☐ Verticalization using a table
- ☐ Bed cycling
- ☐ Electrostimulation
- ☐ "Other (please specify)"
