# Supplementary material for: Practices in sedation, analgesia, mobilization, delirium, and sleep deprivation in adult intensive care units (SAMDS-ICU): an international survey before and during the COVID-19 pandemic
Source: Ann Intensive Care. 2022 Feb 4;12:9. doi: 10.1186/s13613-022-00985-y (PMC8815719; doi:10.1186/s13613-022-00985-y)
Supplement: Supplementary file 2 — Additional file 2: Spanish version of questionnaire. Contains Spanish version of the questionnaire administrated before the COVID-19 pandemic. [file 13613_2022_985_MOESM2_ESM.pdf]

SAMDS

SEDATION, ANALGESIA, MOBILIZATION, DELIRIUM AND SLEEP DEPRIVATION IN ICU

Sedación, analgesia, mobilización, delirium y deprivación de sueño en la UCI
Estudio internacional multicéntrico - SAMDS study

Consentimiento Informado

Nos gustaría invitarlo a participar en esta encuesta relacionada con las prácticas de analgesia, sedación, manejo del delirium y deprivación del sueño en la unidad de cuidados intensivos. Este estudio se realizará mediante un cuestionario auto-administrado (8 minutos de duración) sobre sus prácticas en sedación, analgesia, mobilización, optimización del sueño, así como el diagnóstico, manejo y tratamiento del delirium en su lugar de trabajo.
Los investigadores no han recibido ningún apoyo financiero para la realización del presente estudio, y usted no recibirá compensación económica por participar en esta investigación. Usted no será identificado en este cuestionario. Si acepta participar en este estudio, por favor marque el cuadro de diálogo a continuación para acceder al cuestionario.

En caso de cualquier duda, puede contactar en cualquier momento a los miembros del Comité Directivo.

Comité Directivo SAMDS Estudio:

Bruna Brandão Barreto (brunab_barreto@yahoo.com.br) - Brazil
Mariana Luz (marianaluzmed@gmail.com) - Brazil
Eduardo Tobar (edotobar@gmail.com) - Chile
Audrey De Jong (audreydejong@hotmail.fr) - France
Gérald Chanques (g-chanques@chu-montpellier.fr) - France
John Kress (jkress@medicine.bsd.uchicago.edu) - USA
Yahya Shehabi (yshehabi@ozmail.com.au) - Australia/New Zealand
Roberta Esteves Vieira de Castro (roberta-esteves@hotmail.com) - Brazil
Jorge Salluh (jorgesalluh@gmail.com) - Brazil
Felipe Dal-Pizzol (fdpizzol@gmail.com) - Brazil
Dimitri Gusmao-Flores (dimitrigusmao@gmail.com) - Brazil

* 1. Do you want to participate?

- [ ] Yes

SAMDS

SEDATION, ANALGESIA, MOBILIZATION, DELIRIUM AND SLEEP DEPRIVATION IN ICU

Sedación, analgesia, mobilización, delirium y deprivación de sueño en la UCI
Estudio internacional multicéntrico - SAMDS study

2. ¿En qué país trabaja?

* 3. Edad (años completos):

* 4. ¿Cuánto tiempo lleva trabajando en cuidados intensivos? (años completos)

5. ¿Es usted especialista en cuidados intensivos?

- ○ Si
- ○ No

SAMDS

SEDATION, ANALGESIA, MOBILIZATION, DELIRIUM AND SLEEP DEPRIVATION IN ICU

Sedación, analgesia, mobilización, delirium y deprivación de sueño en la UCI
Estudio internacional multicéntrico - SAMDS study

* 6. ¿Hace cuánto tiempo es un especialista en cuidados intensivos?

SAMDS

SEDATION, ANALGESIA, MOBILIZATION, DELIRIUM AND SLEEP DEPRIVATION IN ICU

Sedación, analgesia, mobilización, delirium y deprivación de sueño en la UCI
Estudio internacional multicéntrico - SAMDS study

Las preguntas a continuación de refieren a la institución donde usted dedica la mayor cantidad de tiempo (horas de trabajo)

7. Tipo de hospital

- ○ Hospital público
- ○ Hospital Universitario
- ○ Hospital privado

8. Tipo de UCI

- ○ Médica
- ○ Cardiaca
- ○ Quirúrgica
- ○ Otra (especifique)
- ○ Mixta
- ○ Neurológica
- ○ Trauma

[ ]

9. Número de camas existentes en su UCI:

- ○ Menos de 10
- ○ Entre 11 y 20
- ○ Más de 20

10. ¿Cuál es la frecuencia de uso de Ventilación Mecánica en los pacientes de su UCI?

- ○ < 20%
- ○ 20-40%
- ○ 40-70%
- ○ > 70%

11. ¿Cuál es su relación enfermera(o): paciente durante el día:

- 1:1
- 1:2
- 1:3
- 1:4
- 1:5
- > 1:5
- No aplica

12. ¿Cuál es su relación enfermera(o): paciente, durante el horario nocturno:

- 1:1
- 1:2
- 1:3
- 1:4
- 1:5
- >1:5
- No aplica

13. ¿Su UCI ha organizado rondas clínicas diarias con un especialista en cuidados intensivos?

- Si
- No

14. ¿Qué profesionales participan en su ronda multidiciplinaria? (marque todas las que apliquen)

- [ ] Médico
- [ ] Enfermera(o)
- [ ] Fisioterapeuta (o Kinesiólogo)
- [ ] Nutricionista
- [ ] Farmaceútico

15. ¿Existe un protocolo de analgesia en su UCI?

- Si
- No
- No lo se

16. ¿Se monitoriza el dolor en los pacientes que son capaces de comunicarse

- Si
- No

Sedación, analgesia, mobilización, delirium y deprivación de sueño en la UCI
Estudio internacional multicéntrico - SAMDS study

17. ¿Cómo monitoriza el dolor en esos pacientes? (marque todas las que apliquen)

- ☐ Escala Visual Análoga (EVA)
- ☐ Escala numérica oral (NRS)
- ☐ Escala conductual del dolor (BPS) y/o BPS para pacientes no intubados
- ☐ Critical-Care Pain Observation Tool (CPOT)
- ☐ Evaluación no estructurada
- ☐ Otra (especifique)

SAMDS
SEDATION, ANALGESIA, MOBILIZATION, DELIRIUM AND SLEEP DEPRIVATION IN ICU

Sedación, analgesia, mobilización, delirium y deprivación de sueño en la UCI
Estudio internacional multicéntrico - SAMDS study

18. ¿Se monitoriza el dolor en los pacientes que son incapaces de comunicarse

- ○ Si
- ○ No

SAMDS
SEDATION, ANALGESIA, MOBILIZATION, DELIRIUM AND SLEEP DEPRIVATION IN ICU

Sedación, analgesia, mobilización, delirium y deprivación de sueño en la UCI
Estudio internacional multicéntrico - SAMDS study

19. ¿Cómo monitoriza el dolor en esos pacientes? (marque todas las que apliquen)

- [ ] Escala Visual Análoga (EVA)
- [ ] Escala numérica oral (NRS)
- [ ] Escala conductual del dolor (BPS) y/o BPS para pacientes no intubados
- [ ] Critical-Care Pain Observation Tool (CPOT)
- [ ] Evaluación no estructurada
- [ ] Otro (especifique)

SAMDS

SEDATION, ANALGESIA, MOBILIZATION, DELIRIUM AND SLEEP DEPRIVATION IN ICU

Sedación, analgesia, mobilización, delirium y deprivación de sueño en la UCI
Estudio internacional multicéntrico - SAMDS study

20. ¿Qué droga usa habitualmente para la analgesia? (marque todas las que apliquen)

- [ ] Midazolam
- [ ] Dipirona (Metamizol)
- [ ] Morfina
- [ ] Fentanyl
- [ ] Remifentanyl
- [ ] Tramadol
- [ ] Gabapentina
- [ ] Propofol
- [ ] Dexmedetomidina
- [ ] Anti-inflamatorios no esteroidales (AINEs)
- [ ] Paracetamol
- [ ] Nefopam
- [ ] Ketamina
- [ ] Otro (especifique)

21. ¿Usted emplea terapia no farmacológica para el tratamiento del dolor?

- ( ) Si
- ( ) No

SAMDS
SEDATION, ANALGESIA, MOBILIZATION, DELIRIUM AND SLEEP DEPRIVATION IN ICU

Sedación, analgesia, mobilización, delirium y deprivación de sueño en la UCI
Estudio internacional multicéntrico - SAMDS study

22. ¿Cuál? (marque todas las que apliquen)

- [ ] Masaje
- [ ] Técnicas de relajación
- [ ] Hipnosis
- [ ] Terapia de frío
- [ ] Ciberterapia
- [ ] Musicoterapia
- [ ] Otra (especifique)

SAMDS
SEDATION, ANALGESIA, MOBILIZATION, DELIRIUM AND SLEEP DEPRIVATION IN ICU

Sedación, analgesia, mobilización, delirium y deprivación de sueño en la UCI
Estudio internacional multicéntrico - SAMDS study

Las preguntas a continuación de refieren a la institución donde usted dedica la mayor cantidad de tiempo (horas de trabajo)

23. ¿Existe un protocolo de sedación en su UCI?

- Si
- No
- No lo se

SAMDS
SEDATION, ANALGESIA, MOBILIZATION, DELIRIUM AND SLEEP DEPRIVATION IN ICU

Sedación, analgesia, mobilización, delirium y deprivación de sueño en la UCI
Estudio internacional multicéntrico - SAMDS study

24. ¿Cuán a menudo usted sigue el protocolo de sedación?

- Nunca
- Algunas veces
- Siempre

SAMDS
SEDATION, ANALGESIA, MOBILIZATION, DELIRIUM AND SLEEP DEPRIVATION IN ICU

Sedación, analgesia, mobilización, delirium y deprivación de sueño en la UCI
Estudio internacional multicéntrico - SAMDS study

25. En su unidad, ¿se usan de forma rutinaria drogas sedantes para los pacientes en ventilación mecánica?

- Si
- No

26. Cuándo usan drogas sedantes para pacientes en ventilación mecánica, ¿Cuál es la estrategia más frecuente?

- Sedación continua con titulación
- Sedación continua con interrupción diaria
- Bolus intermitentes

27. ¿Cuán a menudo son discutidos los objetivos de sedación durante las rondas clínicas?

- ○ Diariamente
- ○ Algunas veces
- ○ Nunca

28. ¿Usted usa rutinariamente alguna escala de sedación?

- ○ Si
- ○ No

SAMDS
SEDATION, ANALGESIA, MOBILIZATION, DELIRIUM AND SLEEP DEPRIVATION IN ICU

Sedación, analgesia, mobilización, delirium y deprivación de sueño en la UCI
Estudio internacional multicéntrico - SAMDS study

29. ¿Qué escala emplea? (marque todas las que apliquen)

- ☐ Ramsay
- ☐ Escala de sedación-agitación SAS
- ☐ Escala de sedación-agitación de Richmond RASS
- ☐ Glasgow
- ☐ Otra (especifique)

SAMDS
SEDATION, ANALGESIA, MOBILIZATION, DELIRIUM AND SLEEP DEPRIVATION IN ICU

Sedación, analgesia, mobilización, delirium y deprivación de sueño en la UCI
Estudio internacional multicéntrico - SAMDS study

30. Cuántas veces al día, se evalúa el nivel de sedación en los pacientes de su UCI?

- ○ 1
- ○ 2
- ○ 3
- ○ >3

* 31. Para mejorar las prácticas de sedación en la UCI, nosotros deberíamos:

	Muy en desacuerdo	En desacuerdo	Neutral	De acuerdo	Muy de acuerdo
Adoptar protocolos escritos de sedación	○	○	○	○	○
Adoptar una escala de sedación estándar	○	○	○	○	○
Monitorizar el nivel de sedación	○	○	○	○	○
Entrenar a las enfermeras a monitorizar el nivel de sedación rutinariamente	○	○	○	○	○
Entrenar a los médicos a monitorizar el nivel de sedación rutinariamente	○	○	○	○	○
Contar con la presencia de un farmacéutico en las visitas	○	○	○	○	○

32. ¿Qué drogas usa habitualmente para la sedación? (marque todas las que apliquen)

- ☐ Midazolam
- ☐ Lorazepam
- ☐ Haloperidol
- ☐ Morfina
- ☐ Fentanyl
- ☐ Otro (especifique)
- ☐ Propofol
- ☐ Remifentanyl
- ☐ Dexmedetomidina
- ☐ Ketamina
- ☐ Quetiapina

33. ¿Existe alguna droga sedante que no use o evite?

- ○ Si
- ○ No

Sedación, analgesia, mobilización, delirium y deprivación de sueño en la UCI
Estudio internacional multicéntrico - SAMDS study

34. ¿Cuál? (marque todas las que apliquen)

- [ ] Midazolam
- [ ] Lorazepam
- [ ] Haloperidol
- [ ] Morfina
- [ ] Fentanyl
- [ ] Otra (especifique)
- [ ] Propofol
- [ ] Remifentanyl
- [ ] Dexmedetomidina
- [ ] Ketamina
- [ ] Quetiapina

Sedación, analgesia, mobilización, delirium y deprivación de sueño en la UCI
Estudio internacional multicéntrico - SAMDS study

¿Qué drogas usted usaría para sedación en cada uno de los escenarios señalados a continuación?

(marque todas las que apliquen)

35. Shock Séptico:

- ☐ Midazolam
- ☐ Lorazepam
- ☐ Haloperidol
- ☐ Morfina
- ☐ Fentanyl
- ☐ Propofol
- ☐ Otra (especifique)
- ☐ Remifentanyl
- ☐ Dexmedetomidina
- ☐ Ketamina
- ☐ Quetiapina
- ☐ Yo no uso sedación

36. Sindrome de Distress Respiratorio Agudo (SDRA) moderado a severo:

- ☐ Midazolam
- ☐ Lorazepam
- ☐ Haloperidol
- ☐ Morfina
- ☐ Fentanyl
- ☐ Propofol
- ☐ Otra (especifique)
- ☐ Remifentanyl
- ☐ Dexmedetomidina
- ☐ Ketamina
- ☐ Quetiapina
- ☐ Yo no uso sedación

37. Paciente agitado en ventilación mecánica no invasiva:

- ☐ Midazolam
- ☐ Lorazepam
- ☐ Haloperidol
- ☐ Morfina
- ☐ Fentanyl
- ☐ Propofol
- ☐ Otra (especifique)
- ☐ Remifentanyl
- ☐ Dexmedetomidina
- ☐ Ketamina
- ☐ Quetiapina
- ☐ Yo no uso sedación

Sedación, analgesia, mobilización, delirium y deprivación de sueño en la UCI
Estudio internacional multicéntrico - SAMDS study

38. ¿Cuán a menudo usa contenciones mecánicas en pacientes en ventilación mecánica?

- Nunca
- Algunas veces
- Siempre

39. ¿Cuán a menudo usa alguna droga para inducir el sueño en pacientes en ventilación mecánica?

- Nunca
- Algunas veces
- Siempre

Sedación, analgesia, mobilización, delirium y deprivación de sueño en la UCI
Estudio internacional multicéntrico - SAMDS study

40. ¿Cuál de las siguientes alternativas emplea habitualmente?

- [ ] Midazolam
- [ ] Otra benzodiacepina
- [ ] Morfina
- [ ] Fentanyl
- [ ] Propofol
- [ ] Otra (especifique)
- [ ] Dexmedetomidina
- [ ] Ketamina
- [ ] Zolpidem
- [ ] Melatonina

SAMDS

SEDATION, ANALGESIA, MOBILIZATION, DELIRIUM AND SLEEP DEPRIVATION IN ICU

Sedación, analgesia, mobilización, delirium y deprivación de sueño en la UCI
Estudio internacional multicéntrico - SAMDS study

41. ¿Usted emplea alguna terapia no farmacológica para promover el sueño en la UCI?

- ( ) Si
- ( ) No

SAMDS

SEDATION, ANALGESIA, MOBILIZATION, DELIRIUM AND SLEEP DEPRIVATION IN ICU

Sedación, analgesia, mobilización, delirium y deprivación de sueño en la UCI
Estudio internacional multicéntrico - SAMDS study

42. ¿Cuál? (marque todas las que apliquen)

- ☐ Tapones en los oídos
- ☐ Reducción de la luz
- ☐ Control del ruido
- ☐ Mascara para dormir
- ☐ Reducir la interrupción del sueño debido a exámenes, baño o administración de medicamentos, etc.

SAMDS
SEDATION, ANALGESIA, MOBILIZATION, DELIRIUM AND SLEEP DEPRIVATION IN ICU

Sedación, analgesia, mobilización, delirium y deprivación de sueño en la UCI
Estudio internacional multicéntrico - SAMDS study

43. ¿Usted tiene información en relación a la frecuencia de delirium en su unidad?

- ◯ Si
- ◯ No

SAMDS
SEDATION, ANALGESIA, MOBILIZATION, DELIRIUM AND SLEEP DEPRIVATION IN ICU

Sedación, analgesia, mobilización, delirium y deprivación de sueño en la UCI
Estudio internacional multicéntrico - SAMDS study

44. ¿Cuál es esa frecuencia?

- ◯ < 10%
- ◯ 10-25%
- ◯ 25-50%
- ◯ 50-75%
- ◯ > 75%

SAMDS

SEDATION, ANALGESIA, MOBILIZATION, DELIRIUM AND SLEEP DEPRIVATION IN ICU

Sedación, analgesia, mobilización, delirium y deprivación de sueño en la UCI
Estudio internacional multicéntrico - SAMDS study

45. ¿Usted evalúa la presencia de delirium?

- ○ Si
- ○ No

SAMDS

SEDATION, ANALGESIA, MOBILIZATION, DELIRIUM AND SLEEP DEPRIVATION IN ICU

Sedación, analgesia, mobilización, delirium y deprivación de sueño en la UCI
Estudio internacional multicéntrico - SAMDS study

46. ¿Quién es evaluado?

- ○ Todos los pacientes
- ○ Solo aquellos pacientes con sospecha clínica

47. ¿Cómo realiza el diagnóstico de delirium? (marque todas las que apliquen)

- ☐ Evaluación clínica general
- ☐ CAM-ICU
- ☐ Delirium rating scale (DRS)
- ☐ Otra (especifique)
- ☐ Intensive care delirium screening checklist (ICDSC)
- ☐ Mini-mental State Examination (MMSEE)

48. ¿Cuántas veces al día se evalúa la presencia de delirium en su unidad?

- ○ 0
- ○ 1
- ○ 2
- ○ >3

SAMDS
SEDATION, ANALGESIA, MOBILIZATION, DELIRIUM AND SLEEP DEPRIVATION IN ICU

Sedación, analgesia, mobilización, delirium y deprivación de sueño en la UCI
Estudio internacional multicéntrico - SAMDS study

49. ¿Qué droga generalmente usa para el tratamiento del delirium? (marque todas las que apliquen)

- ☐ Midazolam
- ☐ Otra benzodiacepina
- ☐ Haloperidol
- ☐ Morfina
- ☐ Fentanyl
- ☐ Otra (especifique)
- ☐ Propofol
- ☐ Dexmedetomidina
- ☐ Antipsicóticos atípicos (olanzapina, quetiapina, clozapina, risperidona)
- ☐ Yo no uso fármacos para tratar el delirium

50. ¿Cómo trata usted el delirium hipoactivo? (marque todas las que apliquen)

- ☐ Terapia farmacológica
- ☐ Terapia no farmacológica
- ☐ Yo no lo trato

SAMDS
SEDATION, ANALGESIA, MOBILIZATION, DELIRIUM AND SLEEP DEPRIVATION IN ICU

51. ¿Qué terapia no farmacológica usted emplea? (marque todas las que apliquen)

- [ ] Música
- [ ] Mobilización
- [ ] Estimulación cognitiva / Terapia Ocupacional
- [ ] Participación de la familia
- [ ] Otra (especifique)

SAMDS
SEDATION, ANALGESIA, MOBILIZATION, DELIRIUM AND SLEEP DEPRIVATION IN ICU

Sedación, analgesia, mobilización, delirium y deprivación de sueño en la UCI
Estudio internacional multicéntrico - SAMDS study

52. ¿Usted realiza movilización precoz en su unidad?

- ○ Si
- ○ Sólo en pacientes sin apoyo de ventilación mecánica
- ○ No

53. ¿Usted cuenta con un equipo de movilización precoz en su unidad?

- ○ Si
- ○ No

54. ¿Qué técnicas de movilización usted usa? (marque todas las que apliquen)

- [ ] Verticalización por staff (sentarse al borde de la cama, ponerse de pie, sentarse en una silla,caminar)
- [ ] Verticalización usando una mesa tilt (tilt table)
- [ ] Bed cycling (pedalear en la cama)
- [ ] Electroestimulación
- [ ] Otra (especifique)
