# Supplementary material for: Practices in sedation, analgesia, mobilization, delirium, and sleep deprivation in adult intensive care units (SAMDS-ICU): an international survey before and during the COVID-19 pandemic
Source: Ann Intensive Care. 2022 Feb 4;12:9. doi: 10.1186/s13613-022-00985-y (PMC8815719; doi:10.1186/s13613-022-00985-y)
Supplement: Supplementary file 3 — Additional file 3: French version of questionnaire. Contains French version of the questionnaire administrated before the COVID-19 pandemic. [file 13613_2022_985_MOESM3_ESM.pdf]

Sédation, Analgésie, Mobilisation, Delirium et Manque de Sommeil en Soins intensifs/Réanimation
Étude multicentrique et internationale - SAMDS Study

Formulaire de Consentement

Nous voudrions vous inviter à participer à une étude sur les pratiques de sédation, analgésie, mobilisation, delirium et sommeil en soins intensifs/réanimation. Cette étude sera réalisée grâce à un questionnaire (durée de 8 minutes), sur votre pratique de sédation, analgésie, mobilisation, gestion du sommeil, ainsi que le dépistage, monitorage et traitement du delirium dans votre lieu de travail.

Les chercheurs n'ont reçu aucun soutien financier pour développer cette étude, et vous ne recevrez pas non plus de compensation financière. Si vous acceptez de participer à cette étude, cliquez sur la boite de dialogue ci-dessous pour avoir accès au questionnaire, s'il vous plait.
N'hésitez pas à contacter les membres du comité directeur de l'étude pour toute question.

Comité Directeur de l'étude SAMDS:

Bruna Brandão Barreto (brunab_barreto@yahoo.com.br) - Brazil
Mariana Luz (marianaluzmed@gmail.com) - Brazil
Eduardo Tobar (edotobar@gmail.com) - Chile
Audrey De Jong (audreydejong@hotmail.fr) - France
Gérald Chanques (g-chanques@chu-montpellier.fr) - France
John Kress (jkress@medicine.bsd.uchicago.edu) - USA
Yahya Shehabi (yshehabi@ozmail.com.au) - Australia/New Zealand
Roberta Esteves Vieira de Castro (roberta-esteves@hotmail.com) - Brazil
Jorge Salluh (jorgesalluh@gmail.com) - Brazil
Felipe Dal-Pizzol (fdpizzol@gmail.com) - Brazil
Dimitri Gusmao-Flores (dimitrigusmao@gmail.com) - Brazil

* 1. Acceptez-vous de participer de l'étude?

☐ Oui

Sédation, Analgésie, Mobilisation, Delirium et Manque de Sommeil en Soins intensifs/Réanimation
Étude multicentrique et internationale - SAMDS Study

* 2. Dans quel pays travaillez vous?

____

* 3. Quel est votre âge(ans)

____

* 4. Depuis combien de temps vous travaillez dans l’Unité de Soins Intensifs / Réanimation ?

____

* 5. Etes-vous spécialiste en Soins Intensifs/ Réanimation ?

- ○ Oui
- ○ Non

SAMDS
SEDATION, ANALGESIA, MOBILIZATION, DELIRIUM AND SLEEP DEPRIVATION IN ICU

Sédation, Analgésie, Mobilisation, Delirium et Manque de Sommeil en Soins intensifs/Réanimation
Étude multicentrique et internationale - SAMDS Study

* 6. Depuis quand vous êtes spécialiste diplômé en Soins Intensifs / Réanimation ?

____

SAMDS
SEDATION, ANALGESIA, MOBILIZATION, DELIRIUM AND SLEEP DEPRIVATION IN ICU

Les réponses ci-dessous concernent l'hôpital où vous travaillez la plupart du temps.

* 7. Il s'agit d'un :

- ◯ Hôpital Public
- ◯ Hôpital Universitaire/ Hôpital d'enseignement
- ◯ Hôpital privé

* 8. Il s'agit d'une Unité de Soins Intensifs / Réanimation :

- ◯ Médicale
- ◯ Cardiaque
- ◯ Chirurgicale
- ◯ Autres
- ◯ Polyvalente (mixte)
- ◯ Neurologique
- ◯ Traumatologique

[ ]

* 9. Combien de lits comporte l'Unité de Soins Intensifs/Réanimation ?

- ◯ 0 à 10
- ◯ 11 à 20
- ◯ plus de 20

* 10. Quelle est la proportion approximative de patients ayant reçu une ventilation mécanique invasive pendant leur séjour en Soins intensifs/Réanimation?

- ◯ Moins 20%
- ◯ 20-40%
- ◯ 40-70%
- ◯ Plus 70%

* 11. Nombre de patients par infirmier (e) (la journée) :

- ◯ 1:1
- ◯ 2:1
- ◯ 3:1
- ◯ 4:1
- ◯ 5:1
- ◯ > 5:1
- ◯ Non applicable

* 12. Nombre de patients par infirmier (e) (la nuit) :

- 1:1
- 2:1
- 3:1
- 4:1
- 5:1
- >5:1
- Non applicable

* 13. Votre unité a-t-elle des visites quotidiennes d'un médecin intensiviste/réanimateur ?

- Oui
- Non

* 14. Quels sont les professionnels intervenant quotidiennement dans l'unité de soins intensifs/réanimation ?

- [ ] Médecin
- [ ] Infirmier (e)
- [ ] Kinésithérapeute
- [ ] Diététicien (e)
- [ ] Pharmacien (e)

* 15. Votre unité a-t-elle un protocole d'analgésie ?

- Oui
- Non
- Je ne sais pas

* 16. Monitorez-vous la douleur chez les patients capables de communiquer ?

- Oui
- Non

SAMDS
SEDATION, ANALGESIA, MOBILIZATION, DELIRIUM AND SLEEP DEPRIVATION IN ICU

* 17. Comment réalisez-vous cette évaluation ? (marquez tout ce qui s'applique)

- ☐ Échelle analogique visuelle
- ☐ Échelle numérique orale
- ☐ Behavioural Pain Scale (BPS) and/or BPS for non intubated patients
- ☐ Critical-Care Pain Observation Tool (CPOT)
- ☐ Evaluation non systématisée
- ☐ Autre (veuillez préciser)

SAMDS
SEDATION, ANALGESIA, MOBILIZATION, DELIRIUM AND SLEEP DEPRIVATION IN ICU

Sédation, Analgésie, Mobilisation, Delirium et Manque de Sommeil en Soins intensifs/Réanimation
Étude multicentrique et internationale - SAMDS Study

* 18. Monitorez-vous la douleur chez les patients qui ne sont pas capables de communiquer ?

- ◯ Oui
- ◯ Non

SAMDS
SEDATION, ANALGESIA, MOBILIZATION, DELIRIUM AND SLEEP DEPRIVATION IN ICU

Sédation, Analgésie, Mobilisation, Delirium et Manque de Sommeil en Soins intensifs/Réanimation
Étude multicentrique et internationale - SAMDS Study

* 19. Comment réalisez-vous cette évaluation ? (marquez tout ce qui s'applique)

- [ ] Échelle analogique visuelle
- [ ] Échelle numérique orale
- [ ] Behavioural Pain Scale (BPS) and/or BPS for non intubated patients
- [ ] Critical-Care Pain Observation Tool (CPOT)
- [ ] Evaluation non systématisée
- [ ] Autre (veuillez préciser)

SAMDS
SEDATION, ANALGESIA, MOBILIZATION, DELIRIUM AND SLEEP DEPRIVATION IN ICU

Sédation, Analgésie, Mobilisation, Delirium et Manque de Sommeil en Soins intensifs/Réanimation
Étude multicentrique et internationale - SAMDS Study

* 20. Quel médicament utilisez-vous habituellement pour traiter la douleur ? (marquez tout ce qui s'applique)

- [ ] Midazolam
- [ ] Dipyrone (metamizole)
- [ ] Morphine
- [ ] Fentanyl
- [ ] Remifentanyl
- [ ] Tramadol
- [ ] Gabapentine
- [ ] Propofol
- [ ] Dexmédétomidine
- [ ] Anti-inflammatoire non stéroïdien
- [ ] Paracétamol
- [ ] Nefopam
- [ ] Ketamine
- [ ] Autre (veuillez préciser)

* 21. Utilisez-vous des thérapies non-pharmacologiques pour traiter la douleur ?

- ( ) Oui
- ( ) Non

SAMDS
SEDATION, ANALGESIA, MOBILIZATION, DELIRIUM AND SLEEP DEPRIVATION IN ICU

Sédation, Analgésie, Mobilisation, Delirium et Manque de Sommeil en Soins intensifs/Réanimation
Étude multicentrique et internationale - SAMDS Study

* 22. Quelles thérapies utilisez-vous ? (marquez tout ce qui s'applique):

- ☐ Massage
- ☐ Hypnose
- ☐ Cyber thérapie
- ☐ Autre (veuillez préciser)
- ☐ Technique de relaxation
- ☐ Technique de glace
- ☐ Musique

SAMDS
SEDATION, ANALGESIA, MOBILIZATION, DELIRIUM AND SLEEP DEPRIVATION IN ICU

Sédation, Analgésie, Mobilisation, Delirium et Manque de Sommeil en Soins intensifs/Réanimation
Étude multicentrique et internationale - SAMDS Study

Les réponses ci-dessous concernent l'hôpital où vous travaillez la plupart du temps

* 23. Votre unité a-t-elle un protocole de sédation?

- ◯ Oui
- ◯ Non
- ◯ Je ne sais pas

SAMDS
SEDATION, ANALGESIA, MOBILIZATION, DELIRIUM AND SLEEP DEPRIVATION IN ICU

Sédation, Analgésie, Mobilisation, Delirium et Manque de Sommeil en Soins intensifs/Réanimation
Étude multicentrique et internationale - SAMDS Study

* 24. A quelle fréquence utilisez-vous ce protocole ?

- ◯ Jamais
- ◯ Parfois
- ◯ Toujours

SAMDS
SEDATION, ANALGESIA, MOBILIZATION, DELIRIUM AND SLEEP DEPRIVATION IN ICU

Sédation, Analgésie, Mobilisation, Delirium et Manque de Sommeil en Soins intensifs/Réanimation
Étude multicentrique et internationale - SAMDS Study

* 25. Dans votre unité, utilisez-vous régulièrement la sédation chez les patients sous ventilation mécanique?

- ◯ Oui
- ◯ Non

* 26. Quand vous utilisez des sédatifs pour les patients sous ventilation mécanique, quelle stratégie utilisez-vous le plus :

- ◯ Sédation continue avec titration
- ◯ Sédation continue avec interruption quotidienne
- ◯ Bolus intermittent

* 27. Les objectifs de sédation sont discutés pendant la visite :

- Tous les jours
- Parfois
- Jamais

* 28. Utilisez-vous une échelle de sédation régulièrement ?

- Oui
- Non

SAMDS
SEDATION, ANALGESIA, MOBILIZATION, DELIRIUM AND SLEEP DEPRIVATION IN ICU

Sédation, Analgésie, Mobilisation, Delirium et Manque de Sommeil en Soins intensifs/Réanimation
Étude multicentrique et internationale - SAMDS Study

* 29. Quelle(s) échelle(s) ? (marquez tout ce qui s'applique)

- Ramsay
- Sedation-Agitation Scale (SAS)
- Richmond Agitation-Sedation Scale (RASS)
- Glasgow
- Autre (veuillez préciser)

SAMDS
SEDATION, ANALGESIA, MOBILIZATION, DELIRIUM AND SLEEP DEPRIVATION IN ICU

* 30. Combien de fois par jour vous évaluez-vous le niveau de sédation des patients dans l'unité ?

- 1
- 2
- 3
- Plus de 3

* 31. Pour améliorer les pratiques de sédation dans la réanimation, nous devons:

	Pas du tout d'accord	Pas D'accord	Neutre	D'accord	Complètement d'accord
Adopter un protocole écrit de sédation:	○	○	○	○	○
Adopter une échelle de sédation standardisée:	○	○	○	○	○
Surveiller le niveau de sédation:	○	○	○	○	○
Entrainer les infirmier(e)s pour surveiller les niveaux de sédation régulièrement:	○	○	○	○	○
Entrainer les médecins pour surveiller les niveaux de sédation régulièrement:	○	○	○	○	○
Avoir un pharmacien pendant les visites:	○	○	○	○	○

* 32. Quel médicament utilisez-vous habituellement pour sédater les patients (marquez tout ce qui s'applique)

- [ ] Midazolam
- [ ] Lorazepam
- [ ] Halopéridol
- [ ] Morphine
- [ ] Fentanyl
- [ ] Propofol
- [ ] Remifentanyl
- [ ] Dexmédétomidine
- [ ] Ketamina
- [ ] Quétiapine
- [ ] Autre (veuillez préciser)

* 33. Existe-t-il des médicaments que vous n’utilisez pas ou que vous évitez d’utiliser comme sédatif ?

- Oui
- Non

SAMDS
SEDATION, ANALGESIA, MOBILIZATION, DELIRIUM AND SLEEP DEPRIVATION IN ICU

Sédation, Analgésie, Mobilisation, Delirium et Manque de Sommeil en Soins intensifs/Réanimation
Étude multicentrique et internationale - SAMDS Study

* 34. Lesquels? (marquez tout ce qui s’applique)

- [ ] Midazolam
- [ ] Propofol
- [ ] Lorazepam
- [ ] Remifentanyl
- [ ] Halopéridol
- [ ] Dexmédétomidine
- [ ] Morphine
- [ ] Ketamine
- [ ] Fentanyl
- [ ] Quétiapine
- [ ] Autre (veuillez préciser)

SAMDS
SEDATION, ANALGESIA, MOBILIZATION, DELIRIUM AND SLEEP DEPRIVATION IN ICU

Sédation, Analgésie, Mobilisation, Delirium et Manque de Sommeil en Soins intensifs/Réanimation
Étude multicentrique et internationale - SAMDS Study

Quel sédatif utiliseriez-vous dans les cas ci-dessous (marquez tout ce qui s’applique) :

* 35. Choc septique

- [ ] Midazolam
- [ ] Lorazepam
- [ ] Halopéridol
- [ ] Morphine
- [ ] Fentanyl
- [ ] Propofol
- [ ] Autre (veuillez préciser)
- [ ] Remifentanyl
- [ ] Dexmédétomidine
- [ ] Ketamine
- [ ] Quétiapine
- [ ] Je n’utilise pas de sédatif

* 36. Syndrome de Détresse Respiratoire Aiguë sévère / modéré:

- [ ] Midazolam
- [ ] Lorazepam
- [ ] Halopéridol
- [ ] Morphine
- [ ] Fentanyl
- [ ] Propofol
- [ ] Autre (veuillez préciser)
- [ ] Remifentanyl
- [ ] Dexmédétomidine
- [ ] Ketamine
- [ ] Quétiapine
- [ ] Je n’utilise pas de sédatif

* 37. Patients agités sous ventilation mécanique **non-invasive:**

- [ ] Midazolam
- [ ] Lorazepam
- [ ] Halopéridol
- [ ] Morphine
- [ ] Fentanyl
- [ ] Propofol
- [ ] Autre (veuillez préciser)
- [ ] Remifentanyl
- [ ] Dexmédétomidine
- [ ] Ketamine
- [ ] Quétiapine
- [ ] Je n’utilise pas de sédatif

SEDATION, ANALGESIA, MOBILIZATION, DELIRIUM AND SLEEP DEPRIVATION IN ICU

Sédation, Analgésie, Mobilisation, Delirium et Manque de Sommeil en Soins intensifs/Réanimation
Étude multicentrique et internationale - SAMDS Study

* 38. Utilisez-vous des contentions physiques chez le patient sous ventilation mécanique ?

- Jamais
- Parfois
- Toujours

* 39. Utilisez vous des médicaments pour induire le sommeil chez les patients en ventilation mécanique ?

- Jamais
- Parfois
- Toujours

SEDATION, ANALGESIA, MOBILIZATION, DELIRIUM AND SLEEP DEPRIVATION IN ICU

Sédation, Analgésie, Mobilisation, Delirium et Manque de Sommeil en Soins intensifs/Réanimation
Étude multicentrique et internationale - SAMDS Study

* 40. Lequel ? (marquez tout ce qui s’applique)

- ☐ Midazolam
- ☐ Autres benzodiazépines
- ☐ Halopéridol
- ☐ Morphine
- ☐ Fentanyl
- ☐ Autre (veuillez préciser)
- ☐ Propofol
- ☐ Dexmédétomidine
- ☐ Kétamine
- ☐ Zolpidem
- ☐ Mélatonine

SAMDS
SEDATION, ANALGESIA, MOBILIZATION, DELIRIUM AND SLEEP DEPRIVATION IN ICU

Sédation, Analgésie, Mobilisation, Delirium et Manque de Sommeil en Soins intensifs/Réanimation
Étude multicentrique et internationale - SAMDS Study

* 41. Utilisez-vous des interventions non-pharmacologiques pour favoriser le sommeil ?

- ○ Oui
- ○ Non

SAMDS
SEDATION, ANALGESIA, MOBILIZATION, DELIRIUM AND SLEEP DEPRIVATION IN ICU

Sédation, Analgésie, Mobilisation, Delirium et Manque de Sommeil en Soins intensifs/Réanimation
Étude multicentrique et internationale - SAMDS Study

* 42. Quelles interventions utilisez-vous ? (marquez tout ce qui s’applique):

- [ ] Bouchon d’oreille
- [ ] Réduction de luminosité
- [ ] Contrôle de bruits de l’ambiant
- [ ] Autre (veuillez préciser)
- [ ] Masque de sommeil
- [ ] Eviter le réveil nocturne des patients en limitant les examens, toilettes, médications, etc

SAMDS
SEDATION, ANALGESIA, MOBILIZATION, DELIRIUM AND SLEEP DEPRIVATION IN ICU

Sédation, Analgésie, Mobilisation, Delirium et Manque de Sommeil en Soins intensifs/Réanimation
Étude multicentrique et internationale - SAMDS Study

* 43. Avez-vous des informations sur la fréquence du delirium dans votre unité?

- ( ) Oui
- ( ) Non

SAMDS
SEDATION, ANALGESIA, MOBILIZATION, DELIRIUM AND SLEEP DEPRIVATION IN ICU

Sédation, Analgésie, Mobilisation, Delirium et Manque de Sommeil en Soins intensifs/Réanimation
Étude multicentrique et internationale - SAMDS Study

* 44. Quelle est la fréquence ?

- Moins de 10%
- 10-25%
- 25-50%
- 50-75%
- Plus de 75%

SAMDS
SEDATION, ANALGESIA, MOBILIZATION, DELIRIUM AND SLEEP DEPRIVATION IN ICU

Sédation, Analgésie, Mobilisation, Delirium et Manque de Sommeil en Soins intensifs/Réanimation
Étude multicentrique et internationale - SAMDS Study

* 45. Recherchez-vous la présence d’un delirium (confusion mentale)?

- Oui
- Non

SAMDS
SEDATION, ANALGESIA, MOBILIZATION, DELIRIUM AND SLEEP DEPRIVATION IN ICU

Sédation, Analgésie, Mobilisation, Delirium et Manque de Sommeil en Soins intensifs/Réanimation
Étude multicentrique et internationale - SAMDS Study

* 46. Cette évaluation est faite chez :

- Tous les patients
- Les patients avec une suspicion clinique

* 47. Si vous diagnostiquez un delirium, comment faites-vous ce diagnostic ? (marquez tout ce qui s'applique)

- [ ] Evaluation non systématisée
- [ ] Confusion Assessment Method for the ICU (CAM-ICU)
- [ ] Delirium rating scale (DRS)
- [ ] Autre (veuillez préciser)
- [ ] Intensive care delirium screening checklist (ICDSC)
- [ ] Mini-mental State Examination (MMSEE)

* 48. Combien de fois par jour évaluez-vous la présence du delirium chez les patients dans votre unité ?

- ( ) 0
- ( ) 1
- ( ) 2
- ( ) plus de 3

SAMDS
SEDATION, ANALGESIA, MOBILIZATION, DELIRIUM AND SLEEP DEPRIVATION IN ICU

Sédation, Analgésie, Mobilisation, Delirium et Manque de Sommeil en Soins intensifs/Réanimation
Étude multicentrique et internationale - SAMDS Study

* 49. Quel médicament utilisez-vous régulièrement pour traiter le delirium ? (marquez tout ce qui s'applique)

- [ ] Midazolam
- [ ] Autres benzodiazépines
- [ ] Halopéridol
- [ ] Morphine
- [ ] Fentanyl
- [ ] Autre (veuillez préciser)
- [ ] Propofol
- [ ] Dexmédétomidine
- [ ] Antipsychotique atypique (Olanzapine, Quétiapine, Rispéridone)
- [ ] Je ne utilise pas de médicament

* 50. Comment traitez-vous le delirium hypoactif (sans agitation) ? (marquez tout ce qui s’applique)

- [ ] Thérapie pharmacologique
- [ ] Thérapie non-pharmacologique
- [ ] Je ne traite pas

SAMDS
SEDATION, ANALGESIA, MOBILIZATION, DELIRIUM AND SLEEP DEPRIVATION IN ICU

Sédation, Analgésie, Mobilisation, Delirium et Manque de Sommeil en Soins intensifs/Réanimation
Étude multicentrique et internationale - SAMDS Study

51. Quelles thérapies non-pharmacologiques utilisez-vous? (marquez tout ce qui s’applique)

- [ ] Musique
- [ ] Mobilisation
- [ ] Stimulation cognitive / Thérapie occupationnelle
- [ ] Engagement de la famille
- [ ] Autres (especifique)

SAMDS
SEDATION, ANALGESIA, MOBILIZATION, DELIRIUM AND SLEEP DEPRIVATION IN ICU

Sédation, Analgésie, Mobilisation, Delirium et Manque de Sommeil en Soins intensifs/Réanimation
Étude multicentrique et internationale - SAMDS Study

52. Réalisez-vous une mobilisation précoce chez les patients en soins/intensifs/réanimation ?

- ◯ Oui
- ◯ Seulement chez les patients non-ventilés
- ◯ Non

53. Avez-vous une équipe de mobilisation précoce dans votre unité ?

- ◯ Oui
- ◯ Non

54. Quelles techniques de mobilisation utilisez-vous ?

- ☐ Verticalisation par l’équipe soignante
- ☐ Verticalisation avec une table
- ☐ Vélo au lit
- ☐ Électrostimulation
- ☐ Autres (especifique)
