# Supplementary material for: Practices in sedation, analgesia, mobilization, delirium, and sleep deprivation in adult intensive care units (SAMDS-ICU): an international survey before and during the COVID-19 pandemic
Source: Ann Intensive Care. 2022 Feb 4;12:9. doi: 10.1186/s13613-022-00985-y (PMC8815719; doi:10.1186/s13613-022-00985-y)
Supplement: Supplementary file 4 — Additional file 4: Portuguese version of the questionnaire. Contains the Portuguese version of the questionnaire administrated before the COVID-19 pandemic. [file 13613_2022_985_MOESM4_ESM.pdf]

Sedação, Analgesia, Mobilização, Delirium e Privação de Sono em UTI
Estudo Multicêntrico e Internacional - SAMDS Study

Termo de consentimento livre e esclarecido

Gostaríamos de convidar você para participar deste estudo sobre práticas de sedação, analgesia, mobilização, *delirium* e privação de sono em unidades de terapia intensiva. Este estudo será realizado através de um questionário auto-aplicável, com duração estimada de 08 minutos, sobre sua prática e estratégias de sedação, analgesia, mobilização, controle de privação de sono, bem como forma de rastreio, monitorização e tratamento de *delirium* no seu ambiente de trabalho.

Os investigadores não são remunerados para a realização dessa pesquisa, assim como você não receberá benefícios financeiros para sua participação. Todas as informações coletadas serão mantidas em sigilo. Você pode ou não participar da pesquisa. Se concordar com sua participação deverá clicar na caixa de diálogo abaixo para ter acesso ao questionário.

Dúvidas poderão ser esclarecidas, a qualquer momento, com o comitê do estudo.

Comitê SAMDS Study:

Bruna Brandão Barreto (brunab_barreto@yahoo.com.br) - Brazil
Mariana Luz (marianaluzmed@gmail.com) - Brazil
Eduardo Tobar (edotobar@gmail.com) - Chile
Audrey De Jong (audreydejong@hotmail.fr) - France
Gérald Chanques (g-chanques@chu-montpellier.fr) - France
John Kress (jkress@medicine.bsd.uchicago.edu) - USA
Yahya Shehabi (yshehabi@ozmail.com.au) - Australia/New Zealand
Roberta Esteves Vieira de Castro (roberta-esteves@hotmail.com) - Brazil
Jorge Salluh (jorgesalluh@gmail.com) - Brazil
Felipe Dal-Pizzol (fdpizzol@gmail.com) - Brazil
Dimitri Gusmao-Flores (dimitrigusmao@gmail.com) - Brazil

* 1. Aceita Participar?

☐ Sim

Sedação, Analgesia, Mobilização, Delirium e Privação de Sono em UTI
Estudo Multicêntrico e Internacional - SAMDS Study

* 2. Em que país você trabalha?

* 3. Idade (anos completos):

* 4. Tempo de prática em medicina intensiva (anos completos):

5. Especialista em medicina intensiva:

- Sim
- Não

SAMDS
SEDATION, ANALGESIA, MOBILIZATION, DELIRIUM AND SLEEP DEPRIVATION IN ICU

Sedação, Analgesia, Mobilização, Delirium e Privação de Sono em UTI
Estudo Multicêntrico e Internacional - SAMDS Study

* 6. Tempo de titulação em medicina intensiva (anos completos):

SAMDS
SEDATION, ANALGESIA, MOBILIZATION, DELIRIUM AND SLEEP DEPRIVATION IN ICU

As respostas seguintes se referem ao local onde você dedica a maior carga horária:

* 7. Local principal em que atua:

- Hospital Publico
- Hospital Universitário / Hospital de ensino
- Hospital Privado

* 8. Tipo de UTI

- Clínica
- Cirúrgica
- Mista
- Neurológica
- Trauma
- Outro (especifique)

* 9. Número de leitos em sua UTI:

- Até 10
- 11-20
- >20

* 10. Qual o percentual aproximado de pacientes em ventilação mecânica na sua UTI?

- <20%
- 20-40%
- 40-70%
- >70%

* 11. Relação enfermagem : paciente (dia):

- 1:1
- 1:2
- 1:3
- 1:4
- 1:5
- >1:5

* 12. Relação enfermagem : paciente (noite):

- 1:1
- 1:2
- 1:3
- 1:4
- 1:5
- >1:5

* 13. Sua UTI tem visitas diárias com médico intensivista?

- Sim
- Não

* 14. As suas visitas diárias contam com (marque todos que se aplicam):

- [ ] Médico
- [ ] Enfermeiro
- [ ] Fisioterapeuta
- [ ] Nutricionista
- [ ] Farmacêutico

* 15. Sua UTI possui um protocolo de analgesia?

- Sim
- Não
- Não sei

* 16. Você monitoriza dor na sua unidade para pacientes comunicantes?

- Sim
- Não

Sedação, Analgesia, Mobilização, Delirium e Privação de Sono em UTI
Estudo Multicêntrico e Internacional - SAMDS Study

* 17. De que forma faz esta monitorização? (marque todos que se aplicam)

- [ ] Escala analógica visual
- [ ] Escala numérica oral
- [ ] Behavioural Pain Scale (BPS) e ou BPS para pacientes intubados
- [ ] Critical Care Pain Observation Tool (CPOT)
- [ ] Avaliação não estruturada
- [ ] Outro (especifique)

Sedação, Analgesia, Mobilização, Delirium e Privação de Sono em UTI
Estudo Multicêntrico e Internacional - SAMDS Study

As respostas seguintes se referem ao local onde você dedica a maior carga horária:

* 18. Você monitoriza dor na sua unidade para pacientes não comunicantes?

- ( ) Sim
- ( ) Não

SAMDS

SEDATION, ANALGESIA, MOBILIZATION, DELIRIUM AND SLEEP DEPRIVATION IN ICU

Sedação, Analgesia, Mobilização, Delirium e Privação de Sono em UTI
Estudo Multicêntrico e Internacional - SAMDS Study

* 19. De que forma faz esta monitorização? (marque todos que se aplicam)

- [ ] Escala visual
- [ ] Escala numérica oral
- [ ] Behavioural Pain Scale (BPS)
- [ ] Critical Care Pain Observation Tool (CPOT)
- [ ] Avaliação não estruturada
- [ ] Outro (especifique)

SAMDS

SEDATION, ANALGESIA, MOBILIZATION, DELIRIUM AND SLEEP DEPRIVATION IN ICU

Sedação, Analgesia, Mobilização, Delirium e Privação de Sono em UTI
Estudo Multicêntrico e Internacional - SAMDS Study

* 20. Que drogas utiliza habitualmente para a analgesia (marque todos que se aplicam):

- [ ] Midazolam
- [ ] Dipirona (metimazol)
- [ ] Morfina
- [ ] Fentanil
- [ ] Remifentanil
- [ ] Tramadol
- [ ] Gabapentina
- [ ] Propofol
- [ ] Dexmedetomidina
- [ ] Anti-inflamatórios
- [ ] Paracetamol
- [ ] Nefopam
- [ ] Ketamina
- [ ] Outro (especifique)

* 21. Você utiliza estratégia não farmacológica para dor?

- ( ) Sim
- ( ) Não

SAMDS
SEDATION, ANALGESIA, MOBILIZATION, DELIRIUM AND SLEEP DEPRIVATION IN ICU

Sedação, Analgesia, Mobilização, Delirium e Privação de Sono em UTI
Estudo Multicêntrico e Internacional - SAMDS Study

* 22. Quais (marque todas que você utiliza)?

- [ ] Massagem
- [ ] Hipnose
- [ ] Cyberterapia
- [ ] Técnicas de relaxamento
- [ ] Compressa de gelo
- [ ] Musicoterapia
- [ ] Outro (especifique)

SAMDS
SEDATION, ANALGESIA, MOBILIZATION, DELIRIUM AND SLEEP DEPRIVATION IN ICU

Sedação, Analgesia, Mobilização, Delirium e Privação de Sono em UTI
Estudo Multicêntrico e Internacional - SAMDS Study

As respostas seguintes se referem ao local onde você dedica a maior carga horária:

* 23. Sua UTI possui um protocolo de sedação?

- ( ) Sim
- ( ) Não
- ( ) Não sei

SAMDS
SEDATION, ANALGESIA, MOBILIZATION, DELIRIUM AND SLEEP DEPRIVATION IN ICU

Sedação, Analgesia, Mobilização, Delirium e Privação de Sono em UTI
Estudo Multicêntrico e Internacional - SAMDS Study

* 24. Com que frequência você segue o protocolo de sedação?

- Nunca
- As vezes
- Sempre

SAMDS

SEDATION, ANALGESIA, MOBILIZATION, DELIRIUM AND SLEEP DEPRIVATION IN ICU

Sedação, Analgesia, Mobilização, Delirium e Privação de Sono em UTI
Estudo Multicêntrico e Internacional - SAMDS Study

As respostas seguintes se referem ao local onde você dedica a maior carga horária:

* 25. Na sua unidade, você utiliza rotineiramente sedativo para os pacientes em ventilação mecânica:

- Sim
- Não

26. Quando utiliza sedativo para os pacientes em ventilação mecânica, qual a estratégia mais frequentemente utilizada:

- Sedação contínua com titulação
- Sedação contínua com interrupção diária
- Bolus intermitentes

* 27. Metas de sedação são discutidas durante as visitas:

- Diariamente
- As vezes
- Nunca

* 28. Você utiliza alguma escala de sedação de forma rotineira?

- Sim
- Não

SAMDS

SEDATION, ANALGESIA, MOBILIZATION, DELIRIUM AND SLEEP DEPRIVATION IN ICU

Sedação, Analgesia, Mobilização, Delirium e Privação de Sono em UTI
Estudo Multicêntrico e Internacional - SAMDS Study

* 29. Qual?

- ☐ Ramsay
- ☐ Sedation-Agitation Scale (SAS)
- ☐ Richmond Agitation-Sedation Scale (RASS)
- ☐ Glasgow
- ☐ Mais de uma escala
- ☐ Outro (especifique)

SAMDS

SEDATION, ANALGESIA, MOBILIZATION, DELIRIUM AND SLEEP DEPRIVATION IN ICU

Sedação, Analgesia, Mobilização, Delirium e Privação de Sono em UTI
Estudo Multicêntrico e Internacional - SAMDS Study

* 30. Quantas vezes por dia você avalia o nível de sedação dos pacientes na UTI ?

- ○ 1
- ○ 2
- ○ 3
- ○ >3

* 31. Para melhorar as práticas de sedação nas UTIs, devemos:

	Discordo totalmente	Discordo	Neutro	Concordo	Concordo Totalmente
Adotar protocolos escritos de sedação:	○	○	○	○	○
Adotar uma escala de sedação padrão:	○	○	○	○	○
Monitorar o nível de sedação:	○	○	○	○	○
Treinar enfermeiras para monitorar os níveis de sedação rotineiramente:	○	○	○	○	○
Treinar médicos para monitorar os níveis de sedação rotineiramente:	○	○	○	○	○
Ter a presença de um farmacêutico nas visitas:	○	○	○	○	○

* 32. Que drogas utiliza habitualmente para a sedação (marque todos que se aplicam):

- [ ] Midazolam
- [ ] Lorazepam
- [ ] Haloperidol
- [ ] Morfina
- [ ] Fentanil
- [ ] Propofol
- [ ] Remifentanil
- [ ] Dexmedetomidina
- [ ] Ketamina
- [ ] Quetiapina
- [ ] Outro (especifique)

* 33. Existe alguma droga sedativa que você não utiliza ou evita:

- ○ Sim
- ○ Não

SAMDS
SEDATION, ANALGESIA, MOBILIZATION, DELIRIUM AND SLEEP DEPRIVATION IN ICU

Sedação, Analgesia, Mobilização, Delirium e Privação de Sono em UTI
Estudo Multicêntrico e Internacional - SAMDS Study

* 34. Qual (marque todas que se aplicam):

- [ ] Midazolam
- [ ] Lorazepam
- [ ] Haloperidol
- [ ] Morfina
- [ ] Fentanil
- [ ] Propofol
- [ ] Remifentanil
- [ ] Dexmeditomidina
- [ ] Ketamina
- [ ] Quetiapina
- [ ] Outro (especifique)

SAMDS
SEDATION, ANALGESIA, MOBILIZATION, DELIRIUM AND SLEEP DEPRIVATION IN ICU

Sedação, Analgesia, Mobilização, Delirium e Privação de Sono em UTI
Estudo Multicêntrico e Internacional - SAMDS Study

Que drogas para a sedação usaria nos cenários abaixo (marque todos que se aplicam):

* 35. Choque séptico (marque todas que se aplicam):

- [ ] Midazolam
- [ ] Lorazepam
- [ ] Haloperidol
- [ ] Morfina
- [ ] Fentanil
- [ ] Propofol
- [ ] Remifentanil
- [ ] Dexmedetomidina
- [ ] Ketamina
- [ ] Quetiapina
- [ ] Não utilizo sedação

Outro (especifique)

* 36. Síndrome do desconforto respiratório agudo (SDRA) grave / moderada (marque todas que se aplicam):

- [ ] Midazolam
- [ ] Lorazepam
- [ ] Haloperidol
- [ ] Morfina
- [ ] Fentanil
- [ ] Propofol
- [ ] Remifentanil
- [ ] Dexmedetomidina
- [ ] Ketamina
- [ ] Quetiapina
- [ ] Não utilizo sedação
- [ ] Outro (especifique)

* 37. Pacientes agitados e em uso de **Ventilação Não Invasiva - VNI** (marque todas que se aplicam):

- [ ] Midazolam
- [ ] Lorazepam
- [ ] Haloperidol
- [ ] Morfina
- [ ] Fentanil
- [ ] Propofol
- [ ] Remifentanil
- [ ] Dexmedetomidina
- [ ] Ketamina
- [ ] Quetiapina
- [ ] Não utilizo sedação

Outro (especifique)

SAMDS
SEDATION, ANALGESIA, MOBILIZATION, DELIRIUM AND SLEEP DEPRIVATION IN ICU

Sedação, Analgesia, Mobilização, Delirium e Privação de Sono em UTI
Estudo Multicêntrico e Internacional - SAMDS Study

* 38. Você utiliza contenção física em pacientes sob ventilação mecânica:

- ○ Nunca
- ○ As vezes
- ○ Sempre

* 39. Você utiliza alguma medicação para induzir o sono nos pacientes em ventilação mecânica:

- ○ Nunca
- ○ As vezes
- ○ Sempre

Sedação, Analgesia, Mobilização, Delirium e Privação de Sono em UTI
Estudo Multicêntrico e Internacional - SAMDS Study

* 40. Qual (marque todas que se aplicam)?

- [ ] Midazolam
- [ ] Outros benzodiazepínicos
- [ ] Morfina
- [ ] Fentanil
- [ ] Propofol
- [ ] Dexmedetomidina
- [ ] Ketamina
- [ ] Zopidem
- [ ] Melatonina
- [ ] Outro (especifique)

Sedação, Analgesia, Mobilização, Delirium e Privação de Sono em UTI
Estudo Multicêntrico e Internacional - SAMDS Study

* 41. Você utiliza intervenções não farmacológicas para promover sono?

- ( ) Sim
- ( ) Não

Sedação, Analgesia, Mobilização, Delirium e Privação de Sono em UTI
Estudo Multicêntrico e Internacional - SAMDS Study

* 42. Quais (marque todas que se aplicam)?

- [ ] Plugues de ouvido
- [ ] Redução de luminosidade
- [ ] Controle de ruído no ambiente
- [ ] Evitar despertar o paciente a noite para realização de exames, banho, medicações etc.
- [ ] Máscara de dormir
- [ ] Outro (especifique)

Sedação, Analgesia, Mobilização, Delirium e Privação de Sono em UTI
Estudo Multicêntrico e Internacional - SAMDS Study

As respostas seguintes se referem ao local onde você dedica a maior carga horária:

* 43. Você tem informação sobre a frequência de delirium na sua unidade:

- ( ) Sim
- ( ) Não

SAMDS
SEDATION, ANALGESIA, MOBILIZATION, DELIRIUM AND SLEEP DEPRIVATION IN ICU

Sedação, Analgesia, Mobilização, Delirium e Privação de Sono em UTI
Estudo Multicêntrico e Internacional - SAMDS Study

* 44. Qual é esta frequência?

- ◯ <10%
- ◯ 10-25%
- ◯ 25-50%
- ◯ 50-75%
- ◯ >75%

SAMDS
SEDATION, ANALGESIA, MOBILIZATION, DELIRIUM AND SLEEP DEPRIVATION IN ICU

Sedação, Analgesia, Mobilização, Delirium e Privação de Sono em UTI
Estudo Multicêntrico e Internacional - SAMDS Study

* 45. Você investiga a presença de delirium:

- ◯ Sim
- ◯ Não

SAMDS
SEDATION, ANALGESIA, MOBILIZATION, DELIRIUM AND SLEEP DEPRIVATION IN ICU

As respostas seguintes se referem ao local onde você dedica a maior carga horária:

* 46. Esta avaliação é feita em:

- ◯ Todos os pacientes
- ◯ Apenas com suspeita clinica

* 47. Que instrumento usam (marque todas que se aplicam):

- ☐ Avaliação clínica geral
- ☐ Confusion Assessment Method for the ICU (CAM-ICU)
- ☐ Delirium rating scale (DRS)
- ☐ Intensive Care Delirium Screening Checklist (ICDSC)
- ☐ Mini Mental State Examination (MMSE)
- ☐ Outro (especifique)

* 48. Quantas vezes ao dia a presença de delirium é avaliada em sua UTI ?

- ◯ 0
- ◯ 1
- ◯ 2
- ◯ 3
- ◯ >3

SAMDS
SEDATION, ANALGESIA, MOBILIZATION, DELIRIUM AND SLEEP DEPRIVATION IN ICU

Sedação, Analgesia, Mobilização, Delirium e Privação de Sono em UTI
Estudo Multicêntrico e Internacional - SAMDS Study

* 49. Que drogas utiliza habitualmente para tratar o delirium (marque todos que se aplicam):

- [ ] Midazolam
- [ ] Outros benzodiazepínicos
- [ ] Haloperidol
- [ ] Morfina
- [ ] Fentanil
- [ ] Propofol
- [ ] Dexmedetomidina
- [ ] Anti-psicóticos atípicos (Olanzapina, Quetiapina, Clozapina, Risperidona)
- [ ] Nenhuma
- [ ] Outro (especifique)

* 50. Como você trata delirium hipoativo (marque todas que se aplicam):

- [ ] Terapia farmacológica
- [ ] Terapia não farmacológica
- [ ] Não trato

SAMDS
SEDATION, ANALGESIA, MOBILIZATION, DELIRIUM AND SLEEP DEPRIVATION IN ICU

* 51. Quais terapias não farmacológicas você utiliza (marque todas que se aplicam):

- [ ] Música
- [ ] Mobilização
- [ ] Estímulo cognitivo / Terapia ocupacional
- [ ] Engajamento familiar
- [ ] Outro (especifique)

Sedação, Analgesia, Mobilização, Delirium e Privação de Sono em UTI
Estudo Multicêntrico e Internacional - SAMDS Study

As respostas seguintes se referem ao local onde você dedica a maior carga horária:

* 52. Você realiza mobilização precoce na sua unidade:

- ( ) Sim
- ( ) Somente em pacientes não ventilados
- ( ) Não

* 53. Sua unidade tem um grupo voltado somente para mobilização precoce dos pacientes?

- ( ) Sim
- ( ) Não

* 54. Quais técnicas de mobilização você utiliza (marque todas que se aplicam):

- [ ] Verticalização pela equipe (sentar na beira do leito, levantar, sentar na cadeira, caminhar
- [ ] Verticalização utilizando prancha ortostática
- [ ] Cicloergômetro
- [ ] Eletroestimulação
- [ ] Outro (especifique)
