# Supplementary material for: Practices in sedation, analgesia, mobilization, delirium, and sleep deprivation in adult intensive care units (SAMDS-ICU): an international survey before and during the COVID-19 pandemic
Source: Ann Intensive Care. 2022 Feb 4;12:9. doi: 10.1186/s13613-022-00985-y (PMC8815719; doi:10.1186/s13613-022-00985-y)
Supplement: Supplementary file 5 — Additional file 5: English version of the questionnaire—COVID-19. Contains English version of the questionnaire administrated during the COVID-19 pandemic. [file 13613_2022_985_MOESM5_ESM.pdf]

SAMDS
SEDATION, ANALGESIA, MOBILIZATION, DELIRIUM AND SLEEP DEPRIVATION IN ICU

Sedation, Analgesia and Delirium in ICU for patients with COVID-19 - Multi-center and international study - SAMDS Study

Informed Consent

We would like to invite you to participate in this survey on practices of sedation, analgesia and delirium in the intensive care unit for patients with COVID-19. This study will be performed with a self-applied 7-minute questionnaire about your practices of sedation and analgesia, as well as delirium screening, monitoring and treatment in your work environment (ICU for patients with COVID-19).

The researchers did not receive any financial support for doing this study, and you will not have any financial compensation for participating in this research. You will not be identified in this questionnaire. If you agree to participate in this study, please click on the dialog box below to have access to the questionnaire.

The Institutional Review Board of the Universidade do Extremo Sul Catarinense, Santa Catarina, Brazil (the main institution for the study, e.mail: cetica@unesc.net) approved it (ID 3.542.658).

If you have any questions, please feel free to contact our steering committee members.

Steering Committee of the SAMDS study:
Bruna Brandão Barreto (brunab_barreto@yahoo.com.br) - Brazil
Mariana Luz (marianaluzmed@gmail.com) - Brazil
Eduardo Tobar (edotobar@gmail.com) - Chile
Audrey De Jong (audreydejong@hotmail.fr) - France
Gérald Chanques (g-chanques@chu-montpellier.fr) - France
John Kress (jkress@medicine.bsd.uchicago.edu) - USA
Yahya Shehabi (yshehabi@ozmail.com.au) - Australia/New Zealand
Roberta Esteves Vieira de Castro (roberta-esteves@hotmail.com) - Brazil
Jorge Salluh (jorgesalluh@gmail.com) - Brazil
Felipe Dal-Pizzol (fdpizzol@gmail.com) - Brazil
Dimitri Gusmao-Flores (dimitrigusmao@gmail.com) - Brazil

* 1. Do you want to participate?

- [ ] Yes

SAMDS
SEDATION, ANALGESIA, MOBILIZATION, DELIRIUM AND SLEEP DEPRIVATION IN ICU

2. In what country do you work?

* 3. Age (completed years):

* 4. How long have you been working in intensive care (completed years)?

* 5. Are you an intensive care specialist?

- Yes
- No

SAMDS
SEDATION, ANALGESIA, MOBILIZATION, DELIRIUM AND SLEEP DEPRIVATION IN ICU

Sedation, Analgesia and Delirium in ICU for patients with COVID-19 - Multi-center and international study - SAMDS Study

* 6. How long have you been an intensive care specialist (completed years)?

SAMDS
SEDATION, ANALGESIA, MOBILIZATION, DELIRIUM AND SLEEP DEPRIVATION IN ICU

The answers below refer to the place where you dedicate the most of your working hours:

* 7. Type of hospital:

- Public hospital
- University Hospital / Teaching hospital
- Private Hospital

8. Number of beds in your ICU:

- Up to 10
- 11-20
- >20

9. What is the frequency of patients using mechanical ventilation in your ICU?

- <20%
- 20-40%
- 40-70%
- >70%

10. Nursing : patient ratio (daytime):

- 1:1
- 1:2
- 1:3
- 1:4
- 1:5
- >1:5
- Not apply

11. Nursing : patient ratio (nighttime):

- 1:1
- 1:2
- 1:3
- 1:4
- 1:5
- >1:5
- Not apply

12. Has your ICU been organized with daily rounds with an intensive care specialist?

- ◯ Yes
- ◯ No

13. Which professional participate on multidisciplinary rounds (check all that apply)?

- ☐ Doctor
- ☐ Nutritionist
- ☐ Nurse
- ☐ Pharmacist
- ☐ Physiotherapist

14. Is there any analgesia protocol in your ICU?

- ◯ Yes
- ◯ No
- ◯ I don’t know

15. Do you monitor pain in patients that are able to communicate?

- ◯ Yes
- ◯ No

SAMDS
SEDATION, ANALGESIA, MOBILIZATION, DELIRIUM AND SLEEP DEPRIVATION IN ICU

Sedation, Analgesia and Delirium in ICU for patients with COVID-19 - Multi-center and international study - SAMDS Study

16. How do you monitor pain in these patients (check all that apply)?

- ☐ Visual analogic scale
- ☐ Unstructured evaluation
- ☐ Behavioral Pain Scale – BPS and/or BPS for non intubated patients
- ☐ Oral numeric scale
- ☐ Critical-Care Pain Observation Tool - CPOT
- ☐ "Other (please specify)"

SAMDS
SEDATION, ANALGESIA, MOBILIZATION, DELIRIUM AND SLEEP DEPRIVATION IN ICU

Sedation, Analgesia and Delirium in ICU for patients with COVID-19 - Multi-center and international study - SAMDS Study

17. Do you monitor pain in patients that are not able to communicate?

- ○ Yes
- ○ No

SAMDS
SEDATION, ANALGESIA, MOBILIZATION, DELIRIUM AND SLEEP DEPRIVATION IN ICU

Sedation, Analgesia and Delirium in ICU for patients with COVID-19 - Multi-center and international study - SAMDS Study

18. How do you monitor pain in these patients (check all that apply)?

- ☐ Visual analogic scale
- ☐ Behavioral Pain Scale – BPS and/or BPS for non intubated patients
- ☐ Critical-Care Pain Observation Tool - CPOT
- ☐ "Other (please specify)"
- ☐ Unstructured valuation
- ☐ Oral numeric scale

SAMDS
SEDATION, ANALGESIA, MOBILIZATION, DELIRIUM AND SLEEP DEPRIVATION IN ICU

19. Which drugs do you usually use for analgesia (check all that apply)?

- [ ] Midazolam
- [ ] Dipyrone (metamizole)
- [ ] Morphine
- [ ] Fentanyl
- [ ] Remifentanil
- [ ] Tramadol
- [ ] Gabapentine
- [ ] "Other (please specify)"
- [ ] Propofol
- [ ] Dexmedetomidine
- [ ] Nonsteroidal anti-inflammatory drugs
- [ ] Paracetamol
- [ ] Nefopam
- [ ] Ketamine

20. Do you use non pharmacologic therapy to treat pain?

- ( ) Yes
- ( ) No

SAMDS
SEDATION, ANALGESIA, MOBILIZATION, DELIRIUM AND SLEEP DEPRIVATION IN ICU

Sedation, Analgesia and Delirium in ICU for patients with COVID-19 - Multi-center and international study - SAMDS Study

21. Which one (check all that apply)?

- [ ] Massage
- [ ] Hypnosis
- [ ] Cybertherapy
- [ ] Other (please specify)
- [ ] Relaxation techniques
- [ ] Cold therapy
- [ ] Music therapy

SAMDS
SEDATION, ANALGESIA, MOBILIZATION, DELIRIUM AND SLEEP DEPRIVATION IN ICU

Sedation, Analgesia and Delirium in ICU for patients with COVID-19 - Multi-center and international study - SAMDS Study

22. Is there any sedation protocol in your ICU?

- ◯ Yes
- ◯ No
- ◯ I don’t know

SAMDS
SEDATION, ANALGESIA, MOBILIZATION, DELIRIUM AND SLEEP DEPRIVATION IN ICU

Sedation, Analgesia and Delirium in ICU for patients with COVID-19 - Multi-center and international study - SAMDS Study

23. How often do you follow the sedation protocol?

- ◯ Never
- ◯ Sometimes
- ◯ Always

SAMDS
SEDATION, ANALGESIA, MOBILIZATION, DELIRIUM AND SLEEP DEPRIVATION IN ICU

24. In your unit, do you routinely use sedative drugs for patients on mechanical ventilation?

- Yes
- No

25. When using sedative drugs for patients on mechanical ventilation, what is the most frequently used strategy?

- Continuous sedation with titration
- Continuous sedation with daily interruption
- Intermittent bolus

26. How often are the sedation goals discussed during rounds?

- Daily
- Sporadically
- Never

27. Do you use any sedation scale routinely?

- Yes
- No

SAMDS

SEDATION, ANALGESIA, MOBILIZATION, DELIRIUM AND SLEEP DEPRIVATION IN ICU

Sedation, Analgesia and Delirium in ICU for patients with COVID-19 - Multi-center and international study - SAMDS Study

28. Which scale do you use (check all that apply)?

- ☐ Ramsay
- ☐ Sedation-agitation scale - SAS
- ☐ Richmond agitation-sedation scale - RASS
- ☐ Glasgow
- ☐ "Other (please specify)"

SAMDS

SEDATION, ANALGESIA, MOBILIZATION, DELIRIUM AND SLEEP DEPRIVATION IN ICU

Sedation, Analgesia and Delirium in ICU for patients with COVID-19 - Multi-center and international study - SAMDS Study

29. How many times a day do you assess the level of sedation of patients in the ICU?

- ◯ 1
- ◯ 2
- ◯ 3
- ◯ >3

30. Which drugs do you usually use for sedation (check all that apply)?

- ☐ Midazolam
- ☐ Lorazepan
- ☐ Haloperidol
- ☐ Morphine
- ☐ Fentanyl
- ☐ "Other (please specify)"
- ☐ Propofol
- ☐ Remifentanyl
- ☐ Dexmedetomidine
- ☐ Ketamine
- ☐ Quetiapine

31. Is there any sedative drug you do not use or avoid?

- ◯ Yes
- ◯ No

Sedation, Analgesia and Delirium in ICU for patients with COVID-19 - Multi-center and international study - SAMDS Study

32. Which one (check all that apply)?

- [ ] Midazolam
- [ ] Lorazepan
- [ ] Haloperidol
- [ ] Morphine
- [ ] Fentanyl
- [ ] Propofol
- [ ] Remifentanyl
- [ ] Dexmedetomidine
- [ ] Ketamine
- [ ] Quetiapine
- [ ] "Other (please specify)"

SAMDS
SEDATION, ANALGESIA, MOBILIZATION, DELIRIUM AND SLEEP DEPRIVATION IN ICU

Sedation, Analgesia and Delirium in ICU for patients with COVID-19 - Multi-center and international study - SAMDS Study

Which drugs would you use for sedation in each scenario below (check all that apply):

33. Septic shock:

- [ ] Midazolam
- [ ] Lorazepan
- [ ] Haloperidol
- [ ] Morphine
- [ ] Fentanyl
- [ ] Propofol
- [ ] "Other (please specify)"
- [ ] Remifentanyl
- [ ] Dexmedetomidine
- [ ] Ketamine
- [ ] Quetiapine
- [ ] I don't use sedation

34. Moderate or severe Acute Respiratory Distress Syndrome (ARDS)

- [ ] Midazolam
- [ ] Lorazepan
- [ ] Haloperidol
- [ ] Morphine
- [ ] Fentanyl
- [ ] Propofol
- [ ] "Other (please specify)"
- [ ] Remifentanyl
- [ ] Dexmedetomidine
- [ ] Ketamine
- [ ] Quetiapine
- [ ] I don't use sedation

35. Agitated patients on **non-invasive mechanical ventilation**:

- [ ] Midazolam
- [ ] Lorazepan
- [ ] Haloperidol
- [ ] Morphine
- [ ] Fentanyl
- [ ] Propofol
- [ ] "Other (please specify)"
- [ ] Remifentanyl
- [ ] Dexmedetomidine
- [ ] Ketamine
- [ ] Quetiapine
- [ ] I don't use sedation

Sedation, Analgesia and Delirium in ICU for patients with COVID-19 - Multi-center and international study - SAMDS Study

36. How often do you use mechanical restraint in patients on mechanical ventilation?

- Never
- Sometimes
- Always

SAMDS
SEDATION, ANALGESIA, MOBILIZATION, DELIRIUM AND SLEEP DEPRIVATION IN ICU

Sedation, Analgesia and Delirium in ICU for patients with COVID-19 - Multi-center and international study - SAMDS Study

37. Do you have information about the frequency of delirium in your unit?

- Yes
- No

SAMDS
SEDATION, ANALGESIA, MOBILIZATION, DELIRIUM AND SLEEP DEPRIVATION IN ICU

Sedation, Analgesia and Delirium in ICU for patients with COVID-19 - Multi-center and international study - SAMDS Study

38. What is this frequency?

- <10%
- 10-25%
- 25-50%
- 50-75%
- >75%

Sedation, Analgesia and Delirium in ICU for patients with COVID-19 - Multi-center and international study - SAMDS Study

39. Do you investigate the presence of delirium?

- ( ) Yes
- ( ) No

Sedation, Analgesia and Delirium in ICU for patients with COVID-19 - Multi-center and international study - SAMDS Study

40. Who is evaluated?

- ( ) All patients?
- ( ) Only patients with clinical suspicion

41. How do you diagnose delirium? (check all that apply)

- [ ] General clinical evaluation
- [ ] Confusion Assessment Method for the Intensive Care Delirium – CAM-ICU
- [ ] Delirium rating scale
- [ ] "Other (please specify)"
- [ ] Intensive care delirium screening checklist - ICDSC
- [ ] Mini-mental State Examination - MMSE

42. How many times a day is the presence of delirium assessed in your ICU?

- ◯ 0
- ◯ 1
- ◯ 2
- ◯ 3
- ◯ >3

SAMDS
SEDATION, ANALGESIA, MOBILIZATION, DELIRIUM AND SLEEP DEPRIVATION IN ICU

Sedation, Analgesia and Delirium in ICU for patients with COVID-19 - Multi-center and international study - SAMDS Study

43.
Which drugs do you generally use to treat delirium? (check all that apply)

- ☐ Midazolam
- ☐ Other benzodiazepines
- ☐ Haloperidol
- ☐ Morphine
- ☐ Fentanyl
- ☐ 'Other (please specify)'
- ☐ Propofol
- ☐ Dexmedetomidine
- ☐ Atypical antipsychotics (olanzapine, quetiapine, clozapine, risperidone)
- ☐ I don't use drugs to treat delirium

44. How do you treat hypoactive delirium (check all that apply) ?

- ☐ Pharmacological therapy
- ☐ Non-pharmacological therapy
- ☐ I do not treat

SAMDS
SEDATION, ANALGESIA, MOBILIZATION, DELIRIUM AND SLEEP DEPRIVATION IN ICU

45. Which non-pharmacological therapy do you use? (check all that apply)

- [ ] Improve sleep
- [ ] Family engagement
- [ ] Mobilization
- [ ] Music therapy
- [ ] Cognitive stimulation / occupational therapy
- [ ] Other (please specify)

SAMDS

SEDATION, ANALGESIA, MOBILIZATION, DELIRIUM AND SLEEP DEPRIVATION IN ICU

Sedation, Analgesia and Delirium in ICU for patients with COVID-19 - Multi-center and international study - SAMDS Study

Thank you!
