# Supplementary material for: Practices in sedation, analgesia, mobilization, delirium, and sleep deprivation in adult intensive care units (SAMDS-ICU): an international survey before and during the COVID-19 pandemic
Source: Ann Intensive Care. 2022 Feb 4;12:9. doi: 10.1186/s13613-022-00985-y (PMC8815719; doi:10.1186/s13613-022-00985-y)
Supplement: Supplementary file 6 — Additional file 6: Spanish version of the questionnaire—COVID-19. Contains Spanish version of the questionnaire administrated during the COVID-19 pandemic. [file 13613_2022_985_MOESM6_ESM.pdf]

SAMDS
SEDATION, ANALGESIA, MOBILIZATION, DELIRIUM AND SLEEP DEPRIVATION IN ICU

# Sedación, analgesia y delirium en la UCI para pacientes con COVID-19
# Estudio internacional multicéntrico - SAMDS study

## Consentimiento Informado

**Nos gustaría invitarlo a participar en esta encuesta relacionada con las prácticas de analgesia, sedación y manejo del delirium en la unidad de cuidados intensivos para pacientes con COVID-19. Este estudio se realizará mediante un cuestionario auto-administrado (7 minutos de duración), para doctores, sobre sus prácticas en sedación, analgesia, así como el diagnóstico, manejo y tratamiento del delirium en su lugar de trabajo (UCI para pacientes con COVID-19).**

**Los investigadores no han recibido ningún apoyo financiero para la realización del presente estudio, y usted no recibirá compensación económica por participar en esta investigación. Usted no será identificado en este cuestionario. Si acepta participar en este estudio, por favor marque el cuadro de diálogo a continuación para acceder al cuestionario.**

**La Junta de Ética en Investigación de la Universidade do Extremo Sul Catarinense, Santa Catarina, Brasil (correo electrónico: cetica@unesc.net) aprobó este estudio (ID 3.542.658).**

**En caso de cualquier duda, puede contactar en cualquier momento a los miembros del Comité Directivo.**

**Comité Directivo SAMDS Estudio:**


**Bruna Brandão Barreto (brunab_barreto@yahoo.com.br) - Brazil**
**Mariana Luz (marianaluzmed@gmail.com) - Brazil**
**Eduardo Tobar (edotobar@gmail.com) - Chile**
**Audrey De Jong (audreydejong@hotmail.fr) - France**
**Gérald Chanques (g-chanques@chu-montpellier.fr) - France**
**John Kress (jkress@medicine.bsd.uchicago.edu) - USA**
**Yahya Shehabi (yshehabi@ozmail.com.au) - Australia/New Zealand**
**Roberta Esteves Vieira de Castro (roberta-esteves@hotmail.com) - Brazil**
**Jorge Salluh (jorgesalluh@gmail.com) - Brazil**
**Felipe Dal-Pizzol (fdpizzol@gmail.com) - Brazil**
**Dimitri Gusmao-Flores (dimitrigusmao@gmail.com) - Brazil**


* 1. Do you want to participate?

- [ ] Yes

SAMDS
SEDATION, ANALGESIA, MOBILIZATION, DELIRIUM AND SLEEP DEPRIVATION IN ICU

Sedación, analgesia y delirium en la UCI para pacientes con COVID-19
Estudio internacional multicéntrico - SAMDS study

2. ¿En qué país trabaja?

* 3. Edad (años completos):

* 4. ¿Cuánto tiempo lleva trabajando en cuidados intensivos? (años completos)

5. ¿Es usted especialista en cuidados intensivos?

- Si
- No

SAMDS
SEDATION, ANALGESIA, MOBILIZATION, DELIRIUM AND SLEEP DEPRIVATION IN ICU

Sedación, analgesia y delirium en la UCI para pacientes con COVID-19
Estudio internacional multicéntrico - SAMDS study

* 6. ¿Hace cuánto tiempo es un especialista en cuidados intensivos?

Sedación, analgesia y delirium en la UCI para pacientes con COVID-19
Estudio internacional multicéntrico - SAMDS study

7. Tipo de hospital

- Hospital público
- Hospital Universitario
- Hospital privado

8. Número de camas existentes en su UCI:

- Menos de 10
- Entre 11 y 20
- Más de 20

9. ¿Cuál es la frecuencia de uso de Ventilación Mecánica en los pacientes de su UCI?

- < 20%
- 20-40%
- 40-70%
- > 70%

10. ¿Cuál es su relación enfermera(o): paciente durante el día:

- 1:1
- 1:2
- 1:3
- 1:4
- 1:5
- > 1:5
- No aplica

11. ¿Cuál es su relación enfermera(o): paciente, durante el horario nocturno:

- ◯ 1:1
- ◯ 1:2
- ◯ 1:3
- ◯ 1:4
- ◯ 1:5
- ◯ >1:5
- ◯ No aplica

12. ¿Su UCI ha organizado rondas clínicas diarias con un especialista en cuidados intensivos?

- ◯ Si
- ◯ No

13. ¿Qué profesionales participan en su ronda multidiciplinaria? (marque todas las que apliquen)

- ☐ Médico
- ☐ Enfermera(o)
- ☐ Fisioterapeuta (o Kinesiólogo)
- ☐ Nutricionista
- ☐ Farmaceútico

14. ¿Existe un protocolo de analgesia en su UCI?

- ◯ Si
- ◯ No
- ◯ No lo se

15. ¿Se monitoriza el dolor en los pacientes que son capaces de comunicarse

- ◯ Si
- ◯ No

SAMDS
SEDATION, ANALGESIA, MOBILIZATION, DELIRIUM AND SLEEP DEPRIVATION IN ICU

16. ¿Cómo monitoriza el dolor en esos pacientes? (marque todas las que apliquen)

- [ ] Escala Visual Análoga (EVA)
- [ ] Escala numérica oral (NRS)
- [ ] Escala conductual del dolor (BPS) y/o BPS para pacientes no intubados
- [ ] Critical-Care Pain Observation Tool (CPOT)
- [ ] Evaluación no estructurada
- [ ] Otra (especifique)

SAMDS
SEDATION, ANALGESIA, MOBILIZATION, DELIRIUM AND SLEEP DEPRIVATION IN ICU

Sedación, analgesia y delirium en la UCI para pacientes con COVID-19
Estudio internacional multicéntrico - SAMDS study

17. ¿Se monitoriza el dolor en los pacientes que son incapaces de comunicarse

- ( ) Si
- ( ) No

SAMDS
SEDATION, ANALGESIA, MOBILIZATION, DELIRIUM AND SLEEP DEPRIVATION IN ICU

Sedación, analgesia y delirium en la UCI para pacientes con COVID-19
Estudio internacional multicéntrico - SAMDS study

18. ¿Cómo monitoriza el dolor en esos pacientes? (marque todas las que apliquen)

- [ ] Escala Visual Análoga (EVA)
- [ ] Escala numérica oral (NRS)
- [ ] Escala conductual del dolor (BPS) y/o BPS para pacientes no intubados
- [ ] Critical-Care Pain Observation Tool (CPOT)
- [ ] Evaluación no estructurada
- [ ] Otro (especifique)

SAMDS

SEDATION, ANALGESIA, MOBILIZATION, DELIRIUM AND SLEEP DEPRIVATION IN ICU

Sedación, analgesia y delirium en la UCI para pacientes con COVID-19
Estudio internacional multicéntrico - SAMDS study

19. ¿Qué droga usa habitualmente para la analgesia? (marque todas las que apliquen)

- [ ] Midazolam
- [ ] Dipirona (Metamizol)
- [ ] Morfina
- [ ] Fentanyl
- [ ] Remifentanyl
- [ ] Tramadol
- [ ] Gabapentina
- [ ] Otro (especifique)
- [ ] Propofol
- [ ] Dexmedetomidina
- [ ] Anti-inflamatorios no esteroidales (AINEs)
- [ ] Paracetamol
- [ ] Nefopam
- [ ] Ketamina

20. ¿Usted emplea terapia no farmacológica para el tratamiento del dolor?

- ( ) Si
- ( ) No

SAMDS

SEDATION, ANALGESIA, MOBILIZATION, DELIRIUM AND SLEEP DEPRIVATION IN ICU

Sedación, analgesia y delirium en la UCI para pacientes con COVID-19
Estudio internacional multicéntrico - SAMDS study

21. ¿Cuál? (marque todas las que apliquen)

- [ ] Masaje
- [ ] Hipnosis
- [ ] Ciberterapia
- [ ] Técnicas de relajación
- [ ] Terapia de frío
- [ ] Musicoterapia
- [ ] Otra (especifique)

SAMDS
SEDATION, ANALGESIA, MOBILIZATION, DELIRIUM AND SLEEP DEPRIVATION IN ICU

## Sedación, analgesia y delirium en la UCI para pacientes con COVID-19
## Estudio internacional multicéntrico - SAMDS study

22. ¿Existe un protocolo de sedación en su UCI?

- ( ) Si
- ( ) No
- ( ) No lo se

SAMDS
SEDATION, ANALGESIA, MOBILIZATION, DELIRIUM AND SLEEP DEPRIVATION IN ICU

## Sedación, analgesia y delirium en la UCI para pacientes con COVID-19
## Estudio internacional multicéntrico - SAMDS study

23. ¿Cuán a menudo usted sigue el protocolo de sedación?

- ( ) Nunca
- ( ) Algunas veces
- ( ) Siempre

Sedación, analgesia y delirium en la UCI para pacientes con COVID-19
Estudio internacional multicéntrico - SAMDS study

24. En su unidad, ¿se usan de forma rutinaria drogas sedantes para los pacientes en ventilación mecánica?

- Si
- No

25. Cuándo usan drogas sedantes para pacientes en ventilación mecánica, ¿Cuál es la estrategia más frecuente?

- Sedación continua con titulación
- Sedación continua con interrupción diaria
- Bolus intermitentes

26. ¿Cuán a menudo son discutidos los objetivos de sedación durante las rondas clínicas?

- Diariamente
- Algunas veces
- Nunca

27. ¿Usted usa rutinariamente alguna escala de sedación?

- Si
- No

Sedación, analgesia y delirium en la UCI para pacientes con COVID-19
Estudio internacional multicéntrico - SAMDS study

28. ¿Qué escala emplea? (marque todas las que apliquen)

- [ ] Ramsay
- [ ] Escala de sedación-agitación SAS
- [ ] Escala de sedación-agitación de Richmond RASS
- [ ] Glasgow
- [ ] Otra (especifique)

SAMDS
SEDATION, ANALGESIA, MOBILIZATION, DELIRIUM AND SLEEP DEPRIVATION IN ICU

Sedación, analgesia y delirium en la UCI para pacientes con COVID-19
Estudio internacional multicéntrico - SAMDS study

29. Cuántas veces al día, se evalúa el nivel de sedación en los pacientes de su UCI?

- 1
- 2
- 3
- >3

30. ¿Qué drogas usa habitualmente para la sedación? (marque todas las que apliquen)

- ☐ Midazolam
- ☐ Lorazepam
- ☐ Haloperidol
- ☐ Morfina
- ☐ Fentanyl
- ☐ Propofol
- ☐ Remifentanyl
- ☐ Dexmedetomidina
- ☐ Ketamina
- ☐ Quetiapina
- ☐ Otro (especifique)

31. ¿Existe alguna droga sedante que no use o evite?

- ◯ Si
- ◯ No

SAMDS
SEDATION, ANALGESIA, MOBILIZATION, DELIRIUM AND SLEEP DEPRIVATION IN ICU

## Sedación, analgesia y delirium en la UCI para pacientes con COVID-19
## Estudio internacional multicéntrico - SAMDS study

32. ¿Cuál? (marque todas las que apliquen)

- ☐ Midazolam
- ☐ Lorazepam
- ☐ Haloperidol
- ☐ Morfina
- ☐ Fentanyl
- ☐ Propofol
- ☐ Remifentanyl
- ☐ Dexmedetomidina
- ☐ Ketamina
- ☐ Quetiapina
- ☐ Otra (especifique)

SAMDS

SEDATION, ANALGESIA, MOBILIZATION, DELIRIUM AND SLEEP DEPRIVATION IN ICU

Sedación, analgesia y delirium en la UCI para pacientes con COVID-19
Estudio internacional multicéntrico - SAMDS study

¿Qué drogas usted usaría para sedación en cada uno de los escenarios señalados a continuación?

**(marque todas las que apliquen)**

33. Shock Séptico:

- ☐ Midazolam
- ☐ Lorazepam
- ☐ Haloperidol
- ☐ Morfina
- ☐ Fentanyl
- ☐ Propofol
- ☐ Otra (especifique)
- ☐ Remifentanyl
- ☐ Dexmedetomidina
- ☐ Ketamina
- ☐ Quetiapina
- ☐ Yo no uso sedación

34. Sindrome de Distress Respiratorio Agudo (SDRA) moderado a severo:

- ☐ Midazolam
- ☐ Lorazepam
- ☐ Haloperidol
- ☐ Morfina
- ☐ Fentanyl
- ☐ Propofol
- ☐ Otra (especifique)
- ☐ Remifentanyl
- ☐ Dexmedetomidina
- ☐ Ketamina
- ☐ Quetiapina
- ☐ Yo no uso sedación

35. Paciente agitado en ventilación mecánica no invasiva:

- [ ] Midazolam
- [ ] Lorazepam
- [ ] Haloperidol
- [ ] Morfina
- [ ] Fentanyl
- [ ] Propofol
- [ ] Otra (especifique)
- [ ] Remifentanyl
- [ ] Dexmedetomidina
- [ ] Ketamina
- [ ] Quetiapina
- [ ] Yo no uso sedación

SAMDS
SEDATION, ANALGESIA, MOBILIZATION, DELIRIUM AND SLEEP DEPRIVATION IN ICU

Sedación, analgesia y delirium en la UCI para pacientes con COVID-19
Estudio internacional multicéntrico - SAMDS study

36. ¿Cuán a menudo usa contenciones mecánicas en pacientes en ventilación mecánica?

- ( ) Nunca
- ( ) Algunas veces
- ( ) Siempre

SAMDS
SEDATION, ANALGESIA, MOBILIZATION, DELIRIUM AND SLEEP DEPRIVATION IN ICU

Sedación, analgesia y delirium en la UCI para pacientes con COVID-19
Estudio internacional multicéntrico - SAMDS study

37. ¿Usted tiene información en relación a la frecuencia de delirium en su unidad?

- Si
- No

SAMDS
SEDATION, ANALGESIA, MOBILIZATION, DELIRIUM AND SLEEP DEPRIVATION IN ICU

Sedación, analgesia y delirium en la UCI para pacientes con COVID-19
Estudio internacional multicéntrico - SAMDS study

38. ¿Cuál es esa frecuencia?

- < 10%
- 10-25%
- 25-50%
- 50-75%
- > 75%

SAMDS
SEDATION, ANALGESIA, MOBILIZATION, DELIRIUM AND SLEEP DEPRIVATION IN ICU

Sedación, analgesia y delirium en la UCI para pacientes con COVID-19
Estudio internacional multicéntrico - SAMDS study

39. ¿Usted evalúa la presencia de delirium?

- Si
- No

SAMDS
SEDATION, ANALGESIA, MOBILIZATION, DELIRIUM AND SLEEP DEPRIVATION IN ICU

40. ¿Quién es evaluado?

- ◯ Todos los pacientes
- ◯ Solo aquellos pacientes con sospecha clínica

41. ¿Cómo realiza el diagnóstico de delirium? (marque todas las que apliquen)

- ☐ Evaluación clínica general
- ☐ CAM-ICU
- ☐ Delirium rating scale (DRS)
- ☐ Otra (especifique)
- ☐ Intensive care delirium screening checklist (ICDSC)
- ☐ Mini-mental State Examination (MMSEE)

42. ¿Cuántas veces al día se evalúa la presencia de delirium en su unidad?

- ◯ 0
- ◯ 1
- ◯ 2
- ◯ >3

SAMDS
SEDATION, ANALGESIA, MOBILIZATION, DELIRIUM
AND SLEEP DEPRIVATION IN ICU

Sedación, analgesia y delirium en la UCI para pacientes con COVID-19
Estudio internacional multicéntrico - SAMDS study

43. ¿Qué droga generalmente usa para el tratamiento del delirium? (marque todas las que apliquen)

- [ ] Midazolam
- [ ] Otra benzodiacepina
- [ ] Haloperidol
- [ ] Morfina
- [ ] Fentanyl
- [ ] Otra (especifique)
- [ ] Propofol
- [ ] Dexmedetomidina
- [ ] Antipsicóticos atípicos (olanzapina, quetiapina, clozapina, risperidona)
- [ ] Yo no uso fármacos para tratar el delirium

44. ¿Cómo trata usted el delirium hipoactivo? (marque todas las que apliquen)

- [ ] Terapia farmacológica
- [ ] Terapia no farmacológica
- [ ] Yo no lo trato

SAMDS
SEDATION, ANALGESIA, MOBILIZATION, DELIRIUM AND SLEEP DEPRIVATION IN ICU

Sedación, analgesia y delirium en la UCI para pacientes con COVID-19
Estudio internacional multicéntrico - SAMDS study

45. ¿Qué terapia no farmacológica usted emplea? (marque todas las que apliquen)

- [ ] Música
- [ ] Mobilización
- [ ] Estimulación cognitiva / Terapia Ocupacional
- [ ] Participación de la familia
- [ ] Otra (especifique)

Sedación, analgesia y delirium en la UCI para pacientes con COVID-19
Estudio internacional multicéntrico - SAMDS study

Gracias!
