# Supplementary material for: Practices in sedation, analgesia, mobilization, delirium, and sleep deprivation in adult intensive care units (SAMDS-ICU): an international survey before and during the COVID-19 pandemic
Source: Ann Intensive Care. 2022 Feb 4;12:9. doi: 10.1186/s13613-022-00985-y (PMC8815719; doi:10.1186/s13613-022-00985-y)
Supplement: Supplementary file 7 — Additional file 7: French version of the questionnaire—COVID-19. Contains French version of the questionnaire administrated during the COVID-19 pandemic. [file 13613_2022_985_MOESM7_ESM.pdf]

Sédation, Analgésie et Delirium en Soins intensifs/Réanimation pour les patients COVID-19
Étude multicentrique et internationale - SAMDS Study

Formulaire de Consentement

Nous voudrions vous inviter à participer à une étude sur les pratiques de sédation, analgésie et delirium en soins intensifs/réanimation pour les patients COVID-19. Cette étude sera réalisée grâce à un questionnaire (durée de 7 minutes), adressé aux médecins, sur votre pratique de sédation, analgésie, mobilisation, gestion du sommeil, ainsi que sur le dépistage, monitorage et traitement du delirium dans votre lieu de travail (Soins intensifs/Réanimation pour les patients COVID-19).

Les chercheurs n'ont reçu aucun soutien financier pour développer cette étude, et vous ne recevrez pas non plus de compensation financière. Si vous acceptez de participer à cette étude, cliquez sur la boite de dialogue ci-dessous pour avoir accès au questionnaire, s'il vous plait.

Le Comité d'éthique de la recherche de l' Universidade do Extremo Sul Catarinense, Santa Catarina, Brazil (e-mail: cetica@unesc.net) a approuvé cette étude (ID 3.542.658).

N'hésitez pas à contacter les membres du comité directeur de l'étude pour toute question.

Comité Directeur de l'étude SAMDS:

Bruna Brandão Barreto (brunab_barreto@yahoo.com.br) - Brazil
Mariana Luz (marianaluzmed@gmail.com) - Brazil
Eduardo Tobar (edotobar@gmail.com) - Chile
Audrey De Jong (audreydejong@hotmail.fr) - France
Gérald Chanques (g-chanques@chu-montpellier.fr) - France
John Kress (jkress@medicine.bsd.uchicago.edu) - USA
Yahya Shehabi (yshehabi@ozmail.com.au) - Australia/New Zealand
Roberta Esteves Vieira de Castro (roberta-esteves@hotmail.com) - Brazil
Jorge Salluh (jorgesalluh@gmail.com) - Brazil
Felipe Dal-Pizzol (fdpizzol@gmail.com) - Brazil
Dimitri Gusmao-Flores (dimitrigusmao@gmail.com) - Brazil

* 1. Acceptez-vous de participer de l'étude?

☐ Oui

Sédation, Analgésie et Delirium en Soins intensifs/Réanimation pour les patients COVID-19
Étude multicentrique et internationale - SAMDS Study

2. Dans quel pays travaillez-vous ?

* 3. Quel est votre âge(ans)

* 4. Depuis combien d'années vous travaillez dans l'Unité de Soins Intensifs / Réanimation ?

* 5. Etes-vous spécialiste en Soins Intensifs/ Réanimation ?

- Oui
- Non

SAMDS
SEDATION, ANALGESIA, MOBILIZATION, DELIRIUM AND SLEEP DEPRIVATION IN ICU

Sédation, Analgésie et Delirium en Soins intensifs/Réanimation pour les patients COVID-19
Étude multicentrique et internationale - SAMDS Study

* 6. Depuis combien d'années vous êtes spécialiste diplômé en Soins Intensifs / Réanimation ?

* 7. Il s’agit d’un :

- ◯ Hôpital Public
- ◯ Hôpital Universitaire/ Hôpital d’enseignement
- ◯ Hôpital privé

* 8. Combien de lits comporte l’Unité de Soins Intensifs/Réanimation ?

- ◯ 0 à 10
- ◯ 11 à 20
- ◯ plus de 20

* 9. Quelle est la proportion approximative de patients ayant reçu une ventilation mécanique invasive pendant leur séjour en Soins intensifs/Réanimation?

- ◯ Moins 20%
- ◯ 20-40%
- ◯ 40-70%
- ◯ Plus 70%

* 10. Nombre de patients par infirmier (e) (la journée) :

- ◯ 1:1
- ◯ 2:1
- ◯ 3:1
- ◯ 4:1
- ◯ 5:1
- ◯ > 5:1
- ◯ Non applicable

* 11. Nombre de patients par infirmier (e) (la nuit) :

- 1:1
- 2:1
- 3:1
- 4:1
- 5:1
- >5:1
- Non applicable

* 12. Votre unité a-t-elle des visites quotidiennes d’un médecin intensiviste/réanimateur ?

- Oui
- Non

* 13. Quels sont les professionnels intervenant quotidiennement dans l’unité de soins intensifs/réanimation ?

- [ ] Médecin
- [ ] Infirmier (e)
- [ ] Kinésithérapeute
- [ ] Diététicien (e)
- [ ] Pharmacien (e)

* 14. Votre unité a-t-elle un protocole d’analgésie ?

- Oui
- Non
- Je ne sais pas

* 15. Monitorez-vous la douleur chez les patients capables de communiquer ?

- Oui
- Non

SAMDS
SEDATION, ANALGESIA, MOBILIZATION, DELIRIUM AND SLEEP DEPRIVATION IN ICU

* 16. Comment réalisez-vous cette évaluation ? (marquez tout ce qui s’applique)

- ☐ Échelle analogique visuelle
- ☐ Échelle numérique orale
- ☐ Behavioural Pain Scale (BPS) and/or BPS for non intubated patients
- ☐ Critical-Care Pain Observation Tool (CPOT)
- ☐ Evaluation non systématisée
- ☐ Autre (veuillez préciser)

SAMDS
SEDATION, ANALGESIA, MOBILIZATION, DELIRIUM AND SLEEP DEPRIVATION IN ICU

Sédation, Analgésie et Delirium en Soins intensifs/Réanimation pour les patients COVID-19
Étude multicentrique et internationale - SAMDS Study

* 17. Monitorez-vous la douleur chez les patients qui ne sont pas capables de communiquer ?

- ◯ Oui
- ◯ Non

SAMDS
SEDATION, ANALGESIA, MOBILIZATION, DELIRIUM AND SLEEP DEPRIVATION IN ICU

Sédation, Analgésie et Delirium en Soins intensifs/Réanimation pour les patients COVID-19
Étude multicentrique et internationale - SAMDS Study

* 18. Comment réalisez-vous cette évaluation ? (marquez tout ce qui s’applique)

- ☐ Échelle analogique visuelle
- ☐ Échelle numérique orale
- ☐ Behavioural Pain Scale (BPS) and/or BPS for non intubated patients
- ☐ Critical-Care Pain Observation Tool (CPOT)
- ☐ Evaluation non systématisée
- ☐ Autre (veuillez préciser)

SAMDS
SEDATION, ANALGESIA, MOBILIZATION, DELIRIUM AND SLEEP DEPRIVATION IN ICU

Sédation, Analgésie et Delirium en Soins intensifs/Réanimation pour les patients COVID-19
Étude multicentrique et internationale - SAMDS Study

* 19. Quel médicament utilisez-vous habituellement pour traiter la douleur ? (marquez tout ce qui s’applique)

- ☐ Midazolam
- ☐ Dipyrone (metamizole)
- ☐ Morphine
- ☐ Fentanyl
- ☐ Remifentanyl
- ☐ Tramadol
- ☐ Gabapentine
- ☐ Autre (veuillez préciser)
- ☐ Propofol
- ☐ Dexmédétomidine
- ☐ Anti-inflammatoire non stéroïdien
- ☐ Paracétamol
- ☐ Nefopam
- ☐ Ketamine

* 20. Utilisez-vous des thérapies non-pharmacologiques pour traiter la douleur ?

- ◯ Oui
- ◯ Non

SAMDS
SEDATION, ANALGESIA, MOBILIZATION, DELIRIUM AND SLEEP DEPRIVATION IN ICU

Sédation, Analgésie et Delirium en Soins intensifs/Réanimation pour les patients COVID-19
Étude multicentrique et internationale - SAMDS Study

* 21. Quelles thérapies utilisez-vous ? (marquez tout ce qui s'applique):

- [ ] Massage
- [ ] Hypnose
- [ ] Cyber thérapie
- [ ] Technique de relaxation
- [ ] Technique de glace
- [ ] Musique
- [ ] Autre (veuillez préciser)

SAMDS
SEDATION, ANALGESIA, MOBILIZATION, DELIRIUM AND SLEEP DEPRIVATION IN ICU

Sédation, Analgésie et Delirium en Soins intensifs/Réanimation pour les patients COVID-19
Étude multicentrique et internationale - SAMDS Study

* 22. Votre unité a-t-elle un protocole de sédation?

- Oui
- Non
- Je ne sais pas

SAMDS
SEDATION, ANALGESIA, MOBILIZATION, DELIRIUM AND SLEEP DEPRIVATION IN ICU

Sédation, Analgésie et Delirium en Soins intensifs/Réanimation pour les patients COVID-19
Étude multicentrique et internationale - SAMDS Study

* 23. A quelle fréquence utilisez-vous ce protocole ?

- ◯ Jamais
- ◯ Parfois
- ◯ Toujours

SAMDS
SEDATION, ANALGESIA, MOBILIZATION, DELIRIUM AND SLEEP DEPRIVATION IN ICU

Sédation, Analgésie et Delirium en Soins intensifs/Réanimation pour les patients COVID-19
Étude multicentrique et internationale - SAMDS Study

* 24. Dans votre unité, utilisez-vous régulièrement la sédation chez les patients sous ventilation mécanique?

- ◯ Oui
- ◯ Non

* 25. Quand vous utilisez des sédatifs pour les patients sous ventilation mécanique, quelle stratégie utilisez-vous le plus :

- ◯ Sédation continue avec titration
- ◯ Sédation continue avec interruption quotidienne
- ◯ Bolus intermittent

* 26. Les objectifs de sédation sont discutés pendant la visite :

- ○ Tous les jours
- ○ Parfois
- ○ Jamais

* 27. Utilisez-vous une échelle de sédation régulièrement ?

- ○ Oui
- ○ Non

SAMDS
SEDATION, ANALGESIA, MOBILIZATION, DELIRIUM AND SLEEP DEPRIVATION IN ICU

Sédation, Analgésie et Delirium en Soins intensifs/Réanimation pour les patients COVID-19
Étude multicentrique et internationale - SAMDS Study

* 28. Quelle(s) échelle(s) ? (marquez tout ce qui s'applique)

- ☐ Ramsay
- ☐ Sedation-Agitation Scale (SAS)
- ☐ Richmond Agitation-Sedation Scale (RASS)
- ☐ Glasgow
- ☐ Autre (veuillez préciser)

SAMDS
SEDATION, ANALGESIA, MOBILIZATION, DELIRIUM AND SLEEP DEPRIVATION IN ICU

* 29. Combien de fois par jour vous évaluez-vous le niveau de sédation des patients dans l’unité ?

- ◯ 1
- ◯ 2
- ◯ 3
- ◯ Plus de 3

* 30. Quel médicament utilisez-vous habituellement pour sédater les patients (marquez tout ce qui s’applique)

- ☐ Midazolam
- ☐ Lorazepam
- ☐ Halopéridol
- ☐ Morphine
- ☐ Fentanyl
- ☐ Propofol
- ☐ Remifentanyl
- ☐ Dexmédétomidine
- ☐ Ketamina
- ☐ Quétiapine
- ☐ Autre (veuillez préciser)

* 31. Existe-t-il des médicaments que vous n’utilisez pas ou que vous évitez d’utiliser comme sédatif ?

- ◯ Oui
- ◯ Non

SAMDS
SEDATION, ANALGESIA, MOBILIZATION, DELIRIUM AND SLEEP DEPRIVATION IN ICU

Sédation, Analgésie et Delirium en Soins intensifs/Réanimation pour les patients COVID-19
Étude multicentrique et internationale - SAMDS Study

* 32. Lesquels? (marquez tout ce qui s’applique)

- [ ] Midazolam
- [ ] Lorazepam
- [ ] Halopéridol
- [ ] Morphine
- [ ] Fentanyl
- [ ] Propofol
- [ ] Remifentanyl
- [ ] Dexmédétomidine
- [ ] Ketamine
- [ ] Quétiapine
- [ ] Autre (veuillez préciser)

SAMDS
SEDATION, ANALGESIA, MOBILIZATION, DELIRIUM AND SLEEP DEPRIVATION IN ICU

Sédation, Analgésie et Delirium en Soins intensifs/Réanimation pour les patients COVID-19
Étude multicentrique et internationale - SAMDS Study

Quel sédatif utiliseriez-vous dans les cas ci-dessous (marquez tout ce qui s’applique) :

* 33. Choc septique

- [ ] Midazolam
- [ ] Lorazepam
- [ ] Halopéridol
- [ ] Morphine
- [ ] Fentanyl
- [ ] Propofol
- [ ] Remifentanyl
- [ ] Dexmédétomidine
- [ ] Ketamine
- [ ] Quétiapine
- [ ] Je n’utilise pas de sédatif
- [ ] Autre (veuillez préciser)

* 34. Syndrome de Détresse Respiratoire Aiguë sévère / modéré:

- [ ] Midazolam
- [ ] Lorazepam
- [ ] Halopéridol
- [ ] Morphine
- [ ] Fentanyl
- [ ] Propofol
- [ ] Autre (veuillez préciser)
- [ ] Remifentanyl
- [ ] Dexmédétomidine
- [ ] Ketamine
- [ ] Quétiapine
- [ ] Je n’utilise pas de sédatif

* 35. Patients agités sous ventilation mécanique **non-invasive:**

- [ ] Midazolam
- [ ] Lorazepam
- [ ] Halopéridol
- [ ] Morphine
- [ ] Fentanyl
- [ ] Propofol
- [ ] Autre (veuillez préciser)
- [ ] Remifentanyl
- [ ] Dexmédétomidine
- [ ] Ketamine
- [ ] Quétiapine
- [ ] Je n’utilise pas de sédatif

SAMDS
SEDATION, ANALGESIA, MOBILIZATION, DELIRIUM AND SLEEP DEPRIVATION IN ICU

Sédation, Analgésie et Delirium en Soins intensifs/Réanimation pour les patients COVID-19
Étude multicentrique et internationale - SAMDS Study

* 36. Utilisez-vous des contentions physiques chez le patient sous ventilation mécanique ?

- ( ) Jamais
- ( ) Parfois
- ( ) Toujours

SAMDS
SEDATION, ANALGESIA, MOBILIZATION, DELIRIUM
AND SLEEP DEPRIVATION IN ICU

Sédation, Analgésie et Delirium en Soins intensifs/Réanimation pour les patients COVID-19
Étude multicentrique et internationale - SAMDS Study

* 37. Avez-vous des informations sur la fréquence du delirium dans votre unité?

- Oui
- Non

SAMDS
SEDATION, ANALGESIA, MOBILIZATION, DELIRIUM
AND SLEEP DEPRIVATION IN ICU

Sédation, Analgésie et Delirium en Soins intensifs/Réanimation pour les patients COVID-19
Étude multicentrique et internationale - SAMDS Study

* 38. Quelle est la fréquence ?

- Moins de 10%
- 10-25%
- 25-50%
- 50-75%
- Plus de 75%

SAMDS
SEDATION, ANALGESIA, MOBILIZATION, DELIRIUM
AND SLEEP DEPRIVATION IN ICU

Sédation, Analgésie et Delirium en Soins intensifs/Réanimation pour les patients COVID-19
Étude multicentrique et internationale - SAMDS Study

* 39. Recherchez-vous la présence d'un delirium (confusion mentale)?

- ◯ Oui
- ◯ Non

SAMDS
SEDATION, ANALGESIA, MOBILIZATION, DELIRIUM AND SLEEP DEPRIVATION IN ICU

Sédation, Analgésie et Delirium en Soins intensifs/Réanimation pour les patients COVID-19
Étude multicentrique et internationale - SAMDS Study

* 40. Cette évaluation est faite chez :

- ◯ Tous les patients
- ◯ Les patients avec une suspicion clinique

* 41. Si vous diagnostiquez un delirium, comment faites-vous ce diagnostic ? (marquez tout ce qui s'applique)

- ☐ Evaluation non systématisée
- ☐ Confusion Assessment Method for the ICU (CAM-ICU)
- ☐ Delirium rating scale (DRS)
- ☐ Autre (veuillez préciser)
- ☐ Intensive care delirium screening checklist (ICDSC)
- ☐ Mini-mental State Examination (MMSEE)

* 42. Combien de fois par jour évaluez-vous la présence du delirium chez les patients dans votre unité ?

- 0
- 1
- 2
- plus de 3

SAMDS
SEDATION, ANALGESIA, MOBILIZATION, DELIRIUM AND SLEEP DEPRIVATION IN ICU

Sédation, Analgésie et Delirium en Soins intensifs/Réanimation pour les patients COVID-19
Étude multicentrique et internationale - SAMDS Study

* 43. Quel médicament utilisez-vous régulièrement pour traiter le delirium ? (marquez tout ce qui s'applique)

- [ ] Midazolam
- [ ] Autres benzodiazépines
- [ ] Halopéridol
- [ ] Morphine
- [ ] Fentanyl
- [ ] Autre (veuillez préciser)
- [ ] Propofol
- [ ] Dexmédétomidine
- [ ] Antipsychotique atypique (Olanzapine, Quétiapine, Rispéridone)
- [ ] Je ne utilise pas de médicament

* 44. Comment traitez-vous le delirium hypoactif (sans agitation) ? (marquez tout ce qui s'applique)

- [ ] Thérapie pharmacologique
- [ ] Thérapie non-pharmacologique
- [ ] Je ne traite pas

SAMDS
SEDATION, ANALGESIA, MOBILIZATION, DELIRIUM AND SLEEP DEPRIVATION IN ICU

Sédation, Analgésie et Delirium en Soins intensifs/Réanimation pour les patients COVID-19
Étude multicentrique et internationale - SAMDS Study

45. Quelles thérapies non-pharmacologiques utilisez-vous? (marquez tout ce qui s’applique)

- ☐ Musique
- ☐ Mobilisation
- ☐ Stimulation cognitive / Thérapie occupationnelle
- ☐ Engagement de la famille
- ☐ Autres (especifique)

SAMDS
SEDATION, ANALGESIA, MOBILIZATION, DELIRIUM AND SLEEP DEPRIVATION IN ICU

Sédation, Analgésie et Delirium en Soins intensifs/Réanimation pour les patients COVID-19
Étude multicentrique et internationale - SAMDS Study

Merci!
