# Supplementary material for: Practices in sedation, analgesia, mobilization, delirium, and sleep deprivation in adult intensive care units (SAMDS-ICU): an international survey before and during the COVID-19 pandemic
Source: Ann Intensive Care. 2022 Feb 4;12:9. doi: 10.1186/s13613-022-00985-y (PMC8815719; doi:10.1186/s13613-022-00985-y)
Supplement: Supplementary file 8 — Additional file 8: Portuguese version of the questionnaire—COVID-19. Contains Portuguese version of the questionnaire administrated during the COVID-19 pandemic. [file 13613_2022_985_MOESM8_ESM.pdf]

Sedação, Analgesia e Delirium em UTI destinada aos pacientes com COVID-19
Estudo Multicêntrico e Internacional - SAMDS Study

Termo de consentimento livre e esclarecido

Gostaríamos de convidar você para participar deste estudo sobre práticas de sedação, analgesia e *delirium* em unidades de terapia intensiva destinadas a pacientes com COVID-19. Este estudo será realizado através de um questionário auto-aplicável, direcionado para médicos, com duração estimada de 07 minutos, sobre as práticas e estratégias de sedação, analgesia, bem como rastreio, monitorização e tratamento de *delirium* no seu ambiente de trabalho (UTI destinada aos pacientes com COVID-19).

Os investigadores não são remunerados para a realização dessa pesquisa, assim como você não receberá benefícios financeiros para sua participação. Todas as informações coletadas serão mantidas em sigilo. Você pode ou não participar da pesquisa. Se concordar com sua participação deverá clicar na caixa de diálogo abaixo para ter acesso ao questionário.

O comitê de ética da Universidade do Extremo Sul Catarinense, Santa Catarina, Brasil (e-mail: cetica@unesc.net) aprovou este estudo (ID 3.542.658).

Dúvidas poderão ser esclarecidas, a qualquer momento, com o comitê do estudo.

Comitê SAMDS Study:

Bruna Brandão Barreto (brunab_barreto@yahoo.com.br) - Brazil
Mariana Luz (marianaluzmed@gmail.com) - Brazil
Eduardo Tobar (edotobar@gmail.com) - Chile
Audrey De Jong (audreydejong@hotmail.fr) - France
Gérald Chanques (g-chanques@chu-montpellier.fr) - France
John Kress (jkress@medicine.bsd.uchicago.edu) - USA
Yahya Shehabi (yshehabi@ozmail.com.au) - Australia/New Zealand
Roberta Esteves Vieira de Castro (roberta-esteves@hotmail.com) - Brazil
Jorge Salluh (jorgesalluh@gmail.com) - Brazil
Felipe Dal-Pizzol (fdpizzol@gmail.com) - Brazil
Dimitri Gusmao-Flores (dimitrigusmao@gmail.com) - Brazil

* 1. Aceita Participar?

☐ Sim

SAMDS
SEDATION, ANALGESIA, MOBILIZATION, DELIRIUM AND SLEEP DEPRIVATION IN ICU

Sedação, Analgesia e Delirium em UTI destinada aos pacientes com COVID-19
Estudo Multicêntrico e Internacional - SAMDS Study

2. Em que país você trabalha?

* 3. Idade (anos completos):

* 4. Tempo de prática em medicina intensiva (anos completos):

5. Especialista em medicina intensiva:

- Sim
- Não

SAMDS
SEDATION, ANALGESIA, MOBILIZATION, DELIRIUM AND SLEEP DEPRIVATION IN ICU

Sedação, Analgesia e Delirium em UTI destinada aos pacientes com COVID-19
Estudo Multicêntrico e Internacional - SAMDS Study

* 6. Tempo de titulação em medicina intensiva (anos completos):

SEDATION, ANALGESIA, MOBILIZATION, DELIRIUM AND SLEEP DEPRIVATION IN ICU

Sedação, Analgesia e Delirium em UTI destinada aos pacientes com COVID-19
Estudo Multicêntrico e Internacional - SAMDS Study

* 7. Tipo de hospital em que atua:

- ◯ Hospital Publico
- ◯ Hospital Universitário / Hospital de ensino
- ◯ Hospital Privado

* 8. Número de leitos em sua UTI:

- ◯ Até 10
- ◯ 11-20
- ◯ >20

* 9. Qual o percentual aproximado de pacientes em ventilação mecânica na sua UTI?

- ◯ <20%
- ◯ 20-40%
- ◯ 40-70%
- ◯ >70%

* 10. Relação enfermagem : paciente (dia):

- ◯ 1:1
- ◯ 1:2
- ◯ 1:3
- ◯ 1:4
- ◯ 1:5
- ◯ >1:5

* 11. Relação enfermagem : paciente (noite):

- 1:1
- 1:2
- 1:3
- 1:4
- 1:5
- >1:5

* 12. Sua UTI tem visitas diárias com médico intensivista?

- Sim
- Não

* 13. As suas visitas diárias contam com (marque todos que se aplicam):

- Médico
- Enfermeiro
- Fisioterapeuta
- Nutricionista
- Farmacêutico

* 14. Sua UTI possui um protocolo de analgesia?

- Sim
- Não
- Não sei

* 15. Você monitoriza dor na sua unidade para pacientes comunicantes?

- Sim
- Não

SAMDS
SEDATION, ANALGESIA, MOBILIZATION, DELIRIUM AND SLEEP DEPRIVATION IN ICU

Sedação, Analgesia e Delirium em UTI destinada aos pacientes com COVID-19
Estudo Multicêntrico e Internacional - SAMDS Study

* 16. De que forma faz esta monitorização? (marque todos que se aplicam)

- [ ] Escala analógica visual
- [ ] Escala numérica oral
- [ ] Behavioural Pain Scale (BPS) e ou BPS para pacientes intubados
- [ ] Critical Care Pain Observation Tool (CPOT)
- [ ] Avaliação não estruturada
- [ ] Outro (especifique)

Sedação, Analgesia e Delirium em UTI destinada aos pacientes com COVID-19
Estudo Multicêntrico e Internacional - SAMDS Study

As respostas seguintes se referem ao local onde você dedica a maior carga horária:

* 17. Você monitoriza dor na sua unidade para pacientes não comunicantes?

- ( ) Sim
- ( ) Não

Sedação, Analgesia e Delirium em UTI destinada aos pacientes com COVID-19
Estudo Multicêntrico e Internacional - SAMDS Study

* 18. De que forma faz esta monitorização? (marque todos que se aplicam)

- [ ] Escala visual
- [ ] Escala numérica oral
- [ ] Behavioural Pain Scale (BPS)
- [ ] Critical Care Pain Observation Tool (CPOT)
- [ ] Avaliação não estruturada
- [ ] Outro (especifique)

SAMDS

SEDATION, ANALGESIA, MOBILIZATION, DELIRIUM AND SLEEP DEPRIVATION IN ICU

Sedação, Analgesia e Delirium em UTI destinada aos pacientes com COVID-19
Estudo Multicêntrico e Internacional - SAMDS Study

* 19. Que drogas utiliza habitualmente para a analgesia (marque todos que se aplicam):

- [ ] Midazolam
- [ ] Dipirona (metimazol)
- [ ] Morfina
- [ ] Fentanil
- [ ] Remifentanil
- [ ] Tramadol
- [ ] Gabapentina
- [ ] Propofol
- [ ] Dexmedetomidina
- [ ] Anti-inflamatórios
- [ ] Paracetamol
- [ ] Nefopam
- [ ] Ketamina
- [ ] Outro (especifique)

* 20. Você utiliza estratégia não farmacológica para dor?

- ◯ Sim
- ◯ Não

SAMDS
SEDATION, ANALGESIA, MOBILIZATION, DELIRIUM AND SLEEP DEPRIVATION IN ICU

Sedação, Analgesia e Delirium em UTI destinada aos pacientes com COVID-19
Estudo Multicêntrico e Internacional - SAMDS Study

* 21. Quais (marque todas que você utiliza)?

- ☐ Massagem
- ☐ Hipnose
- ☐ Cyberterapia
- ☐ Técnicas de relaxamento
- ☐ Compressa de gelo
- ☐ Musicoterapia
- ☐ Outro (especifique)

SAMDS
SEDATION, ANALGESIA, MOBILIZATION, DELIRIUM AND SLEEP DEPRIVATION IN ICU

Sedação, Analgesia e Delirium em UTI destinada aos pacientes com COVID-19
Estudo Multicêntrico e Internacional - SAMDS Study

* 22. Sua UTI possui um protocolo de sedação?

- Sim
- Não
- Não sei

SAMDS
SEDATION, ANALGESIA, MOBILIZATION, DELIRIUM AND SLEEP DEPRIVATION IN ICU

* 23. Com que frequência você segue o protocolo de sedação?

- Nunca
- As vezes
- Sempre

SAMDS
SEDATION, ANALGESIA, MOBILIZATION, DELIRIUM AND SLEEP DEPRIVATION IN ICU

Sedação, Analgesia e Delirium em UTI destinada aos pacientes com COVID-19
Estudo Multicêntrico e Internacional - SAMDS Study

* 24. Na sua unidade, você utiliza rotineiramente sedativo para os pacientes em ventilação mecânica:

- Sim
- Não

25. Quando utiliza sedativo para os pacientes em ventilação mecânica, qual a estratégia mais frequentemente utilizada:

- ◯ Sedação contínua com titulação
- ◯ Sedação contínua com interrupção diária
- ◯ Bolus intermitentes

* 26. Metas de sedação são discutidas durante as visitas:

- ◯ Diariamente
- ◯ As vezes
- ◯ Nunca

* 27. Você utiliza alguma escala de sedação de forma rotineira?

- ◯ Sim
- ◯ Não

SAMDS

SEDATION, ANALGESIA, MOBILIZATION, DELIRIUM AND SLEEP DEPRIVATION IN ICU

Sedação, Analgesia e Delirium em UTI destinada aos pacientes com COVID-19
Estudo Multicêntrico e Internacional - SAMDS Study

* 28. Qual?

- ☐ Ramsay
- ☐ Sedation-Agitation Scale (SAS)
- ☐ Richmond Agitation-Sedation Scale (RASS)
- ☐ Glasgow
- ☐ Mais de uma escala
- ☐ Outro (especifique)

Sedação, Analgesia e Delirium em UTI destinada aos pacientes com COVID-19
Estudo Multicêntrico e Internacional - SAMDS Study

* 29. Quantas vezes por dia você avalia o nível de sedação dos pacientes na UTI ?

- ○ 1
- ○ 2
- ○ 3
- ○ >3

* 30. Que drogas utiliza habitualmente para a sedação (marque todos que se aplicam):

- ☐ Midazolam
- ☐ Lorazepam
- ☐ Haloperidol
- ☐ Morfina
- ☐ Fentanil
- ☐ Propofol
- ☐ Remifentanil
- ☐ Dexmedetomidina
- ☐ Ketamina
- ☐ Quetiapina
- ☐ Outro (especifique)

* 31. Existe alguma droga sedativa que você não utiliza ou evita:

- ○ Sim
- ○ Não

Sedação, Analgesia e Delirium em UTI destinada aos pacientes com COVID-19
Estudo Multicêntrico e Internacional - SAMDS Study

* 32. Qual (marque todas que se aplicam):

- [ ] Midazolam
- [ ] Lorazepam
- [ ] Haloperidol
- [ ] Morfina
- [ ] Fentanil
- [ ] Propofol
- [ ] Remifentanil
- [ ] Dexmeditomidina
- [ ] Ketamina
- [ ] Quetiapina
- [ ] Outro (especifique)

SAMDS
SEDATION, ANALGESIA, MOBILIZATION, DELIRIUM AND SLEEP DEPRIVATION IN ICU

Sedação, Analgesia e Delirium em UTI destinada aos pacientes com COVID-19
Estudo Multicêntrico e Internacional - SAMDS Study

Que drogas para a sedação usaria nos cenários abaixo (marque todos que se aplicam):

* 33. Choque séptico (marque todas que se aplicam):

- [ ] Midazolam
- [ ] Lorazepam
- [ ] Haloperidol
- [ ] Morfina
- [ ] Fentanil
- [ ] Propofol
- [ ] Remifentanil
- [ ] Dexmedetomidina
- [ ] Ketamina
- [ ] Quetiapina
- [ ] Não utilizo sedação

Outro (especifique)

* 34. Síndrome do desconforto respiratório agudo (SDRA) grave / moderada (marque todas que se aplicam):

- [ ] Midazolam
- [ ] Lorazepam
- [ ] Haloperidol
- [ ] Morfina
- [ ] Fentanil
- [ ] Propofol
- [ ] Remifentanil
- [ ] Dexmedetomidina
- [ ] Ketamina
- [ ] Quetiapina
- [ ] Não utilizo sedação
- [ ] Outro (especifique)

* 35. Pacientes agitados e em uso de **Ventilação Não Invasiva - VNI** (marque todas que se aplicam):

- [ ] Midazolam
- [ ] Lorazepam
- [ ] Haloperidol
- [ ] Morfina
- [ ] Fentanil
- [ ] Propofol
- [ ] Remifentanil
- [ ] Dexmedetomidina
- [ ] Ketamina
- [ ] Quetiapina
- [ ] Não utilizo sedação

Outro (especifique)

SAMDS

SEDATION, ANALGESIA, MOBILIZATION, DELIRIUM AND SLEEP DEPRIVATION IN ICU

Sedação, Analgesia e Delirium em UTI destinada aos pacientes com COVID-19
Estudo Multicêntrico e Internacional - SAMDS Study

* 36. Você utiliza contenção física em pacientes sob ventilação mecânica:

- ○ Nunca
- ○ As vezes
- ○ Sempre

SAMDS

SEDATION, ANALGESIA, MOBILIZATION, DELIRIUM AND SLEEP DEPRIVATION IN ICU

Sedação, Analgesia e Delirium em UTI destinada aos pacientes com COVID-19
Estudo Multicêntrico e Internacional - SAMDS Study

* 37. Você tem informação sobre a frequência de delirium na sua unidade:

- Sim
- Não

SAMDS
SEDATION, ANALGESIA, MOBILIZATION, DELIRIUM AND SLEEP DEPRIVATION IN ICU

Sedação, Analgesia e Delirium em UTI destinada aos pacientes com COVID-19
Estudo Multicêntrico e Internacional - SAMDS Study

* 38. Qual é esta frequência?

- <10%
- 10-25%
- 25-50%
- 50-75%
- >75%

SAMDS
SEDATION, ANALGESIA, MOBILIZATION, DELIRIUM AND SLEEP DEPRIVATION IN ICU

Sedação, Analgesia e Delirium em UTI destinada aos pacientes com COVID-19
Estudo Multicêntrico e Internacional - SAMDS Study

* 39. Você investiga a presença de delirium:

- ◯ Sim
- ◯ Não

SAMDS

SEDATION, ANALGESIA, MOBILIZATION, DELIRIUM AND SLEEP DEPRIVATION IN ICU

Sedação, Analgesia e Delirium em UTI destinada aos pacientes com COVID-19
Estudo Multicêntrico e Internacional - SAMDS Study

As respostas seguintes se referem ao local onde você dedica a maior carga horária:

* 40. Esta avaliação é feita em:

- ◯ Todos os pacientes
- ◯ Apenas com suspeita clinica

* 41. Que instrumento usam (marque todas que se aplicam):

- ☐ Avaliação clínica geral
- ☐ Confusion Assessment Method for the ICU (CAM-ICU)
- ☐ Delirium rating scale (DRS)
- ☐ Intensive Care Delirium Screening Checklist (ICDSC)
- ☐ Mini Mental State Examination (MMSE)
- ☐ Outro (especifique)

* 42. Quantas vezes ao dia a presença de delirium é avaliada em sua UTI ?

- ◯ 0
- ◯ 1
- ◯ 2
- ◯ 3
- ◯ >3

* 43. Que drogas utiliza habitualmente para tratar o delirium (marque todos que se aplicam):

- ☐ Midazolam
- ☐ Outros benzodiazepínicos
- ☐ Haloperidol
- ☐ Morfina
- ☐ Fentanil
- ☐ Propofol
- ☐ Dexmedetomidina
- ☐ Anti-psicóticos atípicos (Olanzapina, Quetiapina, Clozapina, Risperidona)
- ☐ Nenhuma
- ☐ Outro (especifique)

* 44. Como você trata delirium hipoativo (marque todas que se aplicam):

- ☐ Terapia farmacológica
- ☐ Terapia não farmacológica
- ☐ Não trato

SAMDS
SEDATION, ANALGESIA, MOBILIZATION, DELIRIUM AND SLEEP DEPRIVATION IN ICU

Sedação, Analgesia e Delirium em UTI destinada aos pacientes com COVID-19
Estudo Multicêntrico e Internacional - SAMDS Study

* 45. Quais terapias não farmacológicas você utiliza (marque todas que se aplicam):

- [ ] Música
- [ ] Mobilização
- [ ] Estímulo cognitivo / Terapia ocupacional
- [ ] Engajamento familiar
- [ ] Outro (especifique)

SAMDS

SEDATION, ANALGESIA, MOBILIZATION, DELIRIUM AND SLEEP DEPRIVATION IN ICU

Sedação, Analgesia e Delirium em UTI destinada aos pacientes com COVID-19

Estudo Multicêntrico e Internacional - SAMDS Study

Obrigado!
