# Supplementary material for: Practices in sedation, analgesia, mobilization, delirium, and sleep deprivation in adult intensive care units (SAMDS-ICU): an international survey before and during the COVID-19 pandemic
Source: Ann Intensive Care. 2022 Feb 4;12:9. doi: 10.1186/s13613-022-00985-y (PMC8815719; doi:10.1186/s13613-022-00985-y)
Supplement: Supplementary file 10 — Additional file 10: Supplementary Material. Contains the supplementary results of the study. [file 13613_2022_985_MOESM10_ESM.docx]

F**igure S1:** Map showing the worldwide distribution of the respondents before (a) and during the COVID-19 pandemic (b)


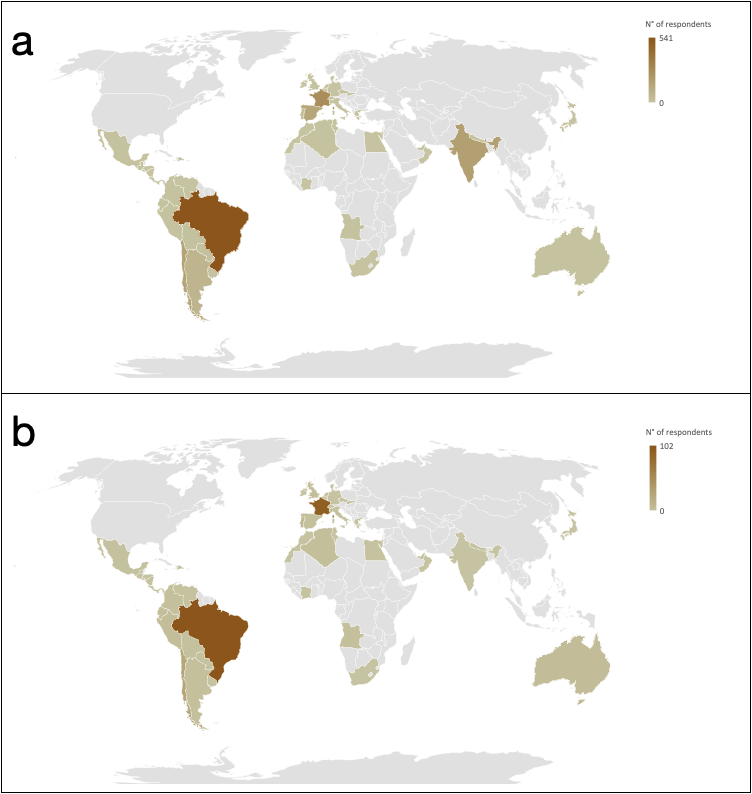


**Table S1** Analgesia practices by continent before the COVID-19 pandemic

| Variables | Asia  % (n) | Europe  % (n) | South America  % (n) |
| --- | --- | --- | --- |
| **Percentage of physicians that assess pain in patients able to communicate^a^** | | | |
|  | 94.9% (168) | 91% (397) | 82.3% (665) |
| **Tools to assess pain in patients able to communicate** | | | |
| Visual analogic scale^b^ | 73.8% (124) | 63.5% (252) | 58.2% (387) |
| BPS^a,c^ | 36.9% (62) | 45.6% (181) | 28% (186) |
| CPOT^a,d^ | 12.5% (21) | 3% (12) | 10.4% (69) |
| Unstructured evaluation^a^ | 16% (27) | 9% (3) | 22.5% (150) |
| Numerical rating scale oral^a^ | 44% (74) | 61.5% (244) | 49% (327) |
| **Percentage of physicians that assess pain in patients unable to communicate^a^** | | | |
|  | 74.1% (129) | 73.7% (316) | 63,0% (493) |
| **Tools to assess pain in patients unable to communicate** | | | |
| Visual analogic scale^a^ | 36.4% (47) | 18.3% (58) | 37.5% (185) |
| BPS^a,c^ | 28.7% (37) | 70.4% (223) | 40.5% (200) |
| CPOT^a, d^ | 42.6% (55) | 6.3% (20) | 22.5% (111) |
| Unstructured evaluation^b^ | 23.3% (30) | 16.1% (51) | 25.1% (124) |
| **Drugs usually used for analgesia** | | | |
| Midazolam^a^ | 21.3% (37) | 10.2% (44) | 19.8% (155) |
| Dipyrone^a^ | 0% (0) | 14.9% (64) | 75.7% (593) |
| Morphine^a^ | 50.6% (88) | 80.7% (347) | 73.8% (578) |
| Fentanyl^a^ | 93.1% (162) | 45.4% (195) | 92.6% (725) |
| Remifentanil^a^ | 0% (0) | 42.6% (183) | 9.1% (79) |
| Tramadol^a^ | 66.1% (115) | 56.5% (243) | 61.1% (478) |
| Gabapentin^b^ | 11.5% (20) | 25.8% (111) | 21.3% (167) |
| Propofol^b^ | 13.8% (24) | 11.4% (49) | 18.8% (147) |
| Dexmedetomidine^a^ | 51.7% (90) | 20.9% (90) | 41.3% (323) |
| Non-steroidal^a^ | 28.2% (49) | 53.3% (229) | 33.6% (263) |
| Paracetamol^a^ | 71.3% (124) | 88.1% (379) | 53.1% (419) |
| Nefopam^a^ | 1.7% (3) | 55.1% (237) | 0.13% (1) |
| Ketamine^a^ | 10.9% (19) | 54.9% (236) | 36.1% (283) |
| **Percentage of physicians that use non-pharmacological therapy for pain management** | | | |
|  | 42.1% (72) | 41% (173) | 40.8% (316) |
| **Types of non-pharmacological pain therapy used** | | | |
| Massage^b^ | 48.6% (35) | 52% (90) | 33.9% (107) |
| Hypnosis^a^ | 1.4% (1) | 35.3% (61) | 0.3% (1) |
| Cyber therapy | 1.4% (1) | 3.5% (6) | 0.6% (2) |
| Relaxation techniques^b^ | 36.1% (26) | 26% (45) | 20.9% (66) |
| Ice pack | 23.6% (17) | 32.4% (56) | 36.1% (114) |
| Music therapy^a^ | 76.4% (55) | 47.4% (82) | 32.6% (103) |

^a^p≤0.001; ^b^<0.05; ^c^Behavioral Pain Score (BPS); ^d^Critical-Care Pain Observation Tool (CPOT);

**Table S2** Univariable analysis non-pharmacological treatment analgesia before the COVID-19 pandemic

| Variables | Use non-pharmacological treatment | | p-value |
| --- | --- | --- | --- |
|  | Yes n (%) | No n (%) |  |
| Physiotherapist participate in multidisciplinary rounds |  |  | < 0.001^a^ |
| Yes | 449 (31.6%) | 556 (39.1%) |  |
| No | 135 (9.5%) | 282 (19.8%) |  |
| There was an analgesia protocol in intensive care unit |  |  | <0.001^a^ |
| Yes | 436 (30.7%) | 521 (36.7%) |  |
| No | 142 (10.0%) | 285 (20.1%) |  |
| I don’t know | 5 (0.4%) | 30 (2.1%) |  |
| Type of hospital |  |  | 0.005^a^ |
| Public hospital | 201 (14.2%) | 340 (23.9%) |  |
| Private hospital | 208 (14.7%) | 233 (16.4%) |  |
| University/Teaching hospital | 175 (12.3%) | 263 (18.5%) |  |
| Nurse participate in multidisciplinary rounds |  |  | 0.01^a^ |
| Yes | 531 (37.3%) | 728 (51.2%) |  |
| No | 53 (3.7%) | 110 (7.8%) |  |
| Nutritionist participate on multidisciplinary rounds |  |  | 0.02^a^ |
| Yes | 269 (18.9%) | 334 (23.5%) |  |
| No | 315 (22.2%) | 504 (35.4%) |  |
| Daily rounds with an intensive care specialist |  |  | 0.03^a^ |
| Yes | 550 (38.7 %) | 764 (53.8%) |  |
| No | 33 (2.3%) | 74 (5.2%) |  |
| Type of intensive care unit |  |  | 0.08^a^ |
| Mixed ICU | 397 (28.0%) | 557 (39.3%) |  |
| Medical | 73 (5.1%) | 139 (9.8%) |  |
| Surgical | 69 (4.8%) | 75 (5.3%) |  |
| Other | 45 (3.2%) | 54 (4.5%) |  |
| Nurse:patient rate (nighttime) |  |  | 0.08^a^ |
| 1:1 | 17 (1.2%) | 25 (1.8%) |  |
| 1:2 | 231 (16.4%) | 335 (23.8%) |  |
| 1:3 | 178 (12.6%) | 301 (21.4%) |  |
| 1:4 | 58 (4.1%) | 67 (4.8%) |  |
| 1:5 | 37 (2.6%) | 32 (2.3%) |  |
| >1:5 | 57 (4.0%) | 70 (5.0%) |  |
| Pharmacist participate in multidisciplinary rounds |  |  | 0.09^a^ |
| Yes | 205 (14.4%) | 239 (16.8%) |  |
| No | 379 (26.7%) | 599 (42.1%) |  |
| Intensive care specialist |  |  | 0.13^a^ |
| Yes | 460 (32.4%) | 630 (44.3%) |  |
| No | 124 (8.7%) | 208 (14.6%) |  |
| Estimated frequency of patients using mechanical ventilation |  |  | 0.29 |
| <20% | 76 (5.4%) | 97 (6.8%) |  |
| 20-40% | 220 (15.5%) | 288 (20.3%) |  |
| 41-70% | 207 (14.6%) | 316 (22.2%) |  |
| >70% | 80 (5.6%) | 137 (9.6%) |  |
| Nurse:patient rate (daytime) |  |  | 0.32 |
| 1:1 | 23 (1.6%) | 36 (2.6%) |  |
| 1:2 | 268 (19.0%) | 399 (28.2%) |  |
| 1:3 | 170 (12.0%) | 260 (18.4%) |  |
| 1:4 | 43 (3.0%) | 51 (3.6%) |  |
| 1:5 | 40 (2.8%) | 36 (2.6%) |  |
| >1:5 | 37 (2.6%) | 51 (3.6%) |  |
| Number of beds in intensive care unit |  |  | <0.39 |
| Up to 10 | 194 (13.7%) | 336 (21.4%) |  |
| 11 – 20 | 239 (16.8%) | 304 (23.7%) |  |
| >20 | 151 (10.6%) | 196 (13.8%) |  |
| Time working in an intensive care |  |  | 0.49 |
| Up to 10 years | 302 (21.2%) | 450 (31.7%) |  |
| Over 10 years | 282 (19.8%) | 388 (27.3%) |  |
| Experience as intensive care specialist |  |  | 0.49 |
| Up to 10 years | 277 (25.5%) | 365 (36.6%) |  |
| Over 10 years | 182 (16.7%) | 263 (24.2%) |  |
| Doctor participate in multidisciplinary rounds |  |  | 0.57 |
| Yes | 578 (40.7 %) | 832 (58.5%) |  |
| No | 6 (0.4%) | 6 (0.4%) |  |

^a^Variables included in logistic regression model

**Table S3** Univariable analysis monitoring pain in patients able to communicate before the COVID-19 pandemic

| Variables | Monitoring pain | | p-value |
| --- | --- | --- | --- |
|  | Yes n (%) | No n (%) |  |
| Type of hospital |  |  | <0.001^a^ |
| Public hospital | 462 (31.4%) | 101 (6.9%) |  |
| Private hospital | 412 (28.0%) | 49 (3.3%) |  |
| University / Teaching hospital | 401 (27.2%) | 48 (3.2%) |  |
| Number of beds in intensive care unit |  |  | <0.001^a^ |
| Up to 10 | 428(29.1%) | 92 (6.2%) |  |
| 11 - 20 | 516(35%) | 76 (5.2%) |  |
| >20 | 331(22.5%) | 30 (2.0%) |  |
| Daily rounds with an intensive care specialist |  |  | <0.001^a^ |
| Yes | 1198 (81.3%) | 164 (11.1%) |  |
| No | 78 (5.3%) | 34 (2.3%) |  |
| Nurse participates in multidisciplinary rounds |  |  | <0.001^a^ |
| Yes | 1152 (78.1%) | 156 (10.6%) |  |
| No | 125 (8.5%) | 42 (2.8%) |  |
| There was an analgesia protocol in intensive care unit |  |  | <0.001^a^ |
| Yes | 909 (61.8 %) | 74 (5.0%) |  |
| No | 334 (22.7%) | 114 (7.7%) |  |
| I don’t know | 31 (2.1 %) | 10 (0.7%) |  |
| Pharmacist participates in multidisciplinary rounds |  |  | 0.006^a^ |
| Yes | 418 (28.3%) | 45 (3.1 %) |  |
| No | 859 (58.2%) | 153 (10.4 %) |  |
| Physiotherapist participates in multidisciplinary rounds |  |  | 0.02^a^ |
| Yes | 918 (62.2 %) | 126 (8.5%) |  |
| No | 359 (24.4%) | 72 (4.9%) |  |
| Type of intensive care unit |  |  | 0.09^a^ |
| Mixed ICU | 838 (56.9%) | 143 (9.7%) |  |
| Medical | 196 (13.3%) | 32 (2.2%) |  |
| Surgical | 136 (9.2%) | 12 (0.8%) |  |
| Others | 104 (7.1%) | 11 (0.7%) |  |
| Nutritionist participates in multidisciplinary rounds |  |  | 0.10^a^ |
| Yes | 552 (37.4 %) | 73 (4.9%) |  |
| No | 725 (49.2%) | 125 (8.5%) |  |
| Time working in an intensive care unit |  |  | 0.30 |
| Up to 10 years | 675 (45.8%) | 113 (7.7%) |  |
| Over 10 years | 602 (40.8%) | 85 (5.7%) |  |
| Doctor participates in multidisciplinary rounds |  |  | 0.71 |
| Yes | 1265 (85.7%) | 197 (13.4%) |  |
| No | 12 (0.8%) | 1 (0.1%) |  |
| Estimated frequency of patients using mechanical ventilation |  |  | 0.86 |
| <20% | 161 (10.9%) | 22 (1.5%) |  |
| 20-40% | 461 (31.3%) | 72 (4.9%) |  |
| 41-70% | 459 (31.1%) | 76 (5.2%) |  |
| >70% | 195 (13.2%) | 28 (1.9%) |  |
| Experience as intensive care specialist |  |  | 0.90 |
| Up to 10 years | 577 (51.4%) | 85 (7.6%) |  |
| Over years | 399 (35.6%) | 61 (5.4%) |  |
| Nurse:patient rate (nighttime) |  |  | 0.92 |
| 1:1 | 39 (2.7 %) | 5 (0.3%) |  |
| 1:2 | 505 (34.6%) | 83 (5.7%) |  |
| 1:3 | 429 (29.4%) | 67 (4.6%) |  |
| 1:4 | 111 (7.6%) | 17 (1.1%) |  |
| 1:5 | 66 (4.5 %) | 7 (0.5%) |  |
| >1:5 | 115 (7.9%) | 17 (1.1%) |  |
| Nurse:patient rate (daytime) |  |  | 0.93 |
| 1:1 | 54 (3.7 %) | 6 (0.4%) |  |
| 1:2 | 599 (40.8 %) | 91 (6.2%) |  |
| 1:3 | 382 (26.0%) | 65 (4.4%) |  |
| 1:4 | 87 (5.9%) | 13 (0.9%) |  |
| 1:5 | 69 (4.7 %) | 10 (0.7%) |  |
| >1:5 | 80 (5.5 %) | 11 (0.8%) |  |

^a^Variables included in the logistic regression model

**Table S4** Variables independently associated with monitoring pain in patients able to communicate before the COVID-19 pandemic

| Variables | OR | CI (2.5 % - 97.5 %) | p-value |
| --- | --- | --- | --- |
| Daily rounds with an intensive care specialist | 1.91 | 1.18 -3.08 | 0.01 |
| Nurse participates in multidisciplinary rounds | 1.64 | 1.01-1.67 | 0.04 |
| There was an analgesia protocol in intensive care unit | 4.43 | 2.05-9.57 | 0.001 |

**Table S5** Univariable analysis monitoring pain in patients unable to communicate

| Variables | Monitoring pain | | p-value |
| --- | --- | --- | --- |
|  | Yes n (%) | No n (%) |  |
| Type of hospital |  |  | <0.001^a^ |
| Public hospital | 325 (22.6%) | 222 (15.4%) |  |
| Private hospital | 315 (22.0%) | 111 (9.4%) |  |
| University / Teaching hospital | 330 (22.9%) | 35 (7.7%) |  |
| Number of beds in intensive care unit |  |  | <0.001^a^ |
| Up to 10 | 301 (20.9%) | 206 (14.3%) |  |
| 11-20 | 406 (28.2%) | 174 (12.1%) |  |
| >20 | 262 (18.3%) | 89 (6.2%) |  |
| Pharmacist participates in multidisciplinary rounds |  |  | <0.001^a^ |
| Yes | 334 (23.2%) | 115 (8.0%) |  |
| No | 637 (44.2%) | 354 (24.6%) |  |
| There was an analgesia protocol in intensive care unit |  |  | <0.001^a^ |
| Yes | 730 (50.8%) | 238 (16.6%) |  |
| No | 221 (15.4%) | 212 (14.8%) |  |
| I don’t know | 18 (1.3%) | 18 (1.3%) |  |
| Daily rounds with an intensive care specialist |  |  | <0.001^a^ |
| Yes | 920 (63.9%) | 411 (28.6%) |  |
| No | 50 (3.5%) | 58 (4.0%) |  |
| Nutritionist participates in multidisciplinary rounds |  |  | 0.006^a^ |
| Yes | 435 (30.2%) | 174 (12.1%) |  |
| No | 536 (37.2%) | 295 (20.5%) |  |
| Nurse participates in multidisciplinary rounds |  |  | 0.009^a^ |
| Yes | 875 (60.8%) | 400 (27.8%) |  |
| No | 96 (6.6%) | 69 (4.8%) |  |
| Estimated frequency of patients using mechanical ventilation |  |  | 0.01^a^ |
| <20% | 101 (7.0%) | 75 (5.2%) |  |
| 20-40% | 362 (25.2%) | 153 (10.6%) |  |
| 41-70% | 362 (25.2%) | 166 (11.5%) |  |
| >70% | 145 (10.1%) | 75 (5.2%) |  |
| Physiotherapist participates in multidisciplinary rounds |  |  | 0.01^a^ |
| Yes | 708 (49.2%) | 312 (21.7%) |  |
| No | 263 (18.2%) | 157 (10.9%) |  |
| Intensive care specialist |  |  | 0.11^a^ |
| Yes | 756 (52.5%) | 347 (24.1%) |  |
| No | 215 (14.9%) | 122 (8.5%) |  |
| Experience as intensive care specialist |  |  | 0.11^a,b^ |
| Up to 10 years | 447 (52.5%) | 756 (24.1%) |  |
| Over10 years | 122 (14.9%) | 215 (8.5%) |  |
| Nurse:patient rate (daytime) |  |  | 0.32 |
| 1:1 | 44 (3.1%) | 16 (1.1%) |  |
| 1:2 | 469 (32.8%) | 208 (14.5%) |  |
| 1:3 | 281 (19.6%) | 155 (10.8%) |  |
| 1:4 | 60 (4.2%) | 35 (2.4%) |  |
| 1:5 | 50 (3.5%) | 26 (1.8%) |  |
| >1:5 | 64 (4.5%) | 24 (1.7%) |  |
| Time working in an intensive care |  |  | 0.30 |
| Up to 10 years | 507 (35.2%) | 259 (18.0%) |  |
| Over 10 years | 464 (32.2%) | 210 (14.6%) |  |
| Doctor participates in multidisciplinary rounds |  |  | 0.53 |
| Yes | 964 (66.9%) | 464 (32.2%) |  |
| No | 7 (0.5%) | 5 (0.4%) |  |
| Types of intensive care unit |  |  | 0.54 |
| Mixed ICU | 655 (45.6 %) | 309 (21.5%) |  |
| Medical | 137 (9.5 %) | 79 (5.5%) |  |
| Surgical | 99 (6.9 %) | 47 (3.3%) |  |
| Others | 78 (5.4 %) | 33 (2.3%) |  |
| Nurse:patient rate (nighttime) |  |  | 0.72 |
| 1:1 | 33 (2.3%) | 11 (0.8%) |  |
| 1:2 | 393 (27.6 %) | 182 (12.8%) |  |
| 1:3 | 317 (22.2 %) | 168 (11.8%) |  |
| 1:4 | 83 (5.8 %) | 42 (2.9%) |  |
| 1:5 | 48 (3.4 %) | 22 (1.5%) |  |
| >1:5 | 89 (6.2 %) | 38 (2.7%) |  |

^a^Variables included in the logistic regression model; ^b^Variable excluded in the logistic regression model due to multicollinearity;

**Table S6** Variables independently associated with monitoring pain in patients non-able to communicate

| Variables | OR | CI (2.5 % - 97.5 %) | p-value |
| --- | --- | --- | --- |
| Daily rounds with an intensive care specialist | 1.74 | 1.12-2.71 | 0.01 |
| There was an analgesia protocol in intensive care unit | 3.22 | 1.23-2.69 | <0.001 |
| Frequency of mechanical ventilation: > 70% | 1.76 | 1.09-2.82 | 0.01 |
| Frequency of mechanical ventilation: 40% - 70% | 1.77 | 1.18-2.66 | 0.005 |
| Frequency of mechanical ventilation: 20% - 40% | 1.72 | 1.17-2.52 | 0.005 |

**Table S7** Univariable analysis monitoring pain in patients unable to communicate using structured tools

| Variables | Use structured tools | | p-value |
| --- | --- | --- | --- |
|  | Yes n (%) | No n (%) |  |
| There was an analgesia protocol in intensive care unit |  |  | <0.001^a^ |
| Yes | 610 (62.7%) | 123 (12.6%) |  |
| No | 150 (15.4%) | 72 (7.4%) |  |
| I don’t know | 15 (1.6%) | 3 (0.3%) |  |
| Type of hospital |  |  | 0.005^a^ |
| Public hospital | 242 (24.9%) | 85 (8.7%) |  |
| Private hospital | 258 (26.5%) | 59 (6.1%) |  |
| University/Teaching hospital | 219 (28.3%) | 54 (5.5%) |  |
| Intensive care specialist |  |  | 0.02^a^ |
| Yes | 616 (63.2%) | 142 (14.6%) |  |
| No | 161 (16.5%) | 52 (5.7%) |  |
| Nutritionist participates in multidisciplinary round |  |  | 0.10^a^ |
| Yes | 358 (36.7 %) | 78 (8%) |  |
| No | 419 (43%) | 120 (12.3%) |  |
| Physiotherapist participates in multidisciplinary rounds |  |  | 0.12^a^ |
| Yes | 575 (59.0%) | 135 (13.8%) |  |
| No | 202 (20.7%) | 63 (6.5%) |  |
| Number of beds in intensive care unit |  |  | 0.16^a^ |
| Up to 10 | 231 (23.7%) | 71 (7.3%) |  |
| 11-20 | 325 (33.5%) | 83 (8.4%) |  |
| >20 | 262 (22.5%) | 45 (4.6%) |  |
| Daily rounds with an intensive care specialist |  |  | 0.16^a^ |
| Yes | 739 (75.9%) | 183 (18.8%) |  |
| No | 37 (3.8%) | 15 (1.5%) |  |
| Nurse participates in multidisciplinary rounds |  |  | 0.21 |
| Yes | 705 (72.3 %) | 173 (17.7%) |  |
| No | 72 (7.4 %) | 25 (2.6%) |  |
| Nurse:patient rate (daytime) |  |  | 0.34 |
| 1:1 | 36 (3.7%) | 8 (0.8%) |  |
| 1:2 | 375 (38.6%) | 95 (9.8%) |  |
| 1:3 | 232 (23.9%) | 51 (5.3%) |  |
| 1:4 | 47 (4.8%) | 14 (1.4%) |  |
| 1:5 | 34 (3.5%) | 16 (1.7%) |  |
| >1:5 | 50 (5.1%) | 14 (1.4 %) |  |
| Pharmacist participates in multidisciplinary rounds |  |  | 0.35 |
| Yes | 373 (28.0%) | 62 (6.4%) |  |
| No | 504 (51.7%) | 136 (13.9%) |  |
| Nurse:patient rate (nighttime) |  |  | 0.49 |
| 1:1 | 25 (2.6%) | 8 (0.8%) |  |
| 1:2 | 315 (32.6 %) | 79 (8.2%) |  |
| 1:3 | 262 (27.1 %) | 57(5.9%) |  |
| 1:4 | 65 (6.7 %) | 19(2.0%) |  |
| 1:5 | 37 (3.8 %) | 11(1.1%) |  |
| >1:5 | 65 (6.7 %) | 24(2.5%) |  |
| Type of intensive care unit |  |  | 0.52 |
| Mixed ICU | 520 (53.4%) | 138 (14.2%) |  |
| Medical | 111 (11.4%) | 27 (2.8 %) |  |
| Surgical | 84 (8.6%) | 15 (1.5%) |  |
| Other | 60 (6.2%) | 18 (1.9%) |  |
| Experience as intensive care specialist |  |  | 0.53 |
| Up to 10 years | 364 (48.2 %) | 79 (10.5%) |  |
| Over 10 years | 250 (33.1 %) | 62 (8.2%) |  |
| Doctor participates in multidisciplinary rounds |  |  | 0.63 |
| Yes | 772 (79.2 %) | 196 (20.1%) |  |
| No | 5 (0.5%) | 2 (0.2%) |  |
| Time working in an intensive care |  |  | 0.68 |
| Up to 10 years | 401 (41.1 %) | 106 (10.9%) |  |
| Over 10 years | 376 (38.6 %) | 92 (9.4%) |  |
| Estimated frequency of patients using mechanical ventilation |  |  | 0.88 |
| <20% | 83 (8.5 %) | 18 (1.8%) |  |
| 20-40% | 287 (29.5 %) | 78 (8.0%) |  |
| 41-70% | 289 (29.7 %) | 73 (7.5%) |  |
| >70% | 117 (12.0 %) | 29 (3.0%) |  |

^a^Variables included in logistic regression model

**Table S8** variables independently associated with monitoring pain in patients non able to communicate using structured tools

| Variables | OR | CI (2.5 % - 97.5 %) | p-value |
| --- | --- | --- | --- |
| Daily rounds with an intensive care specialist | 1.55 | 1.09 - 2.39 | 0.04 |
| There was an analgesia protocol in intensive care unit | 3.26 | 1.62 - 6.56 | <0.001 |

**Figure S2** Sedations scales used before the COVID-19 pandemic

**
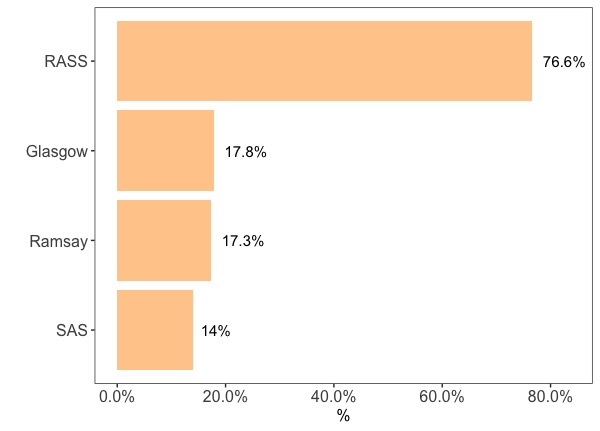
**

**RASS:** Richmond Agitation Sedation Scale; **SAS:** Sedation*-*Agitation Scale;

**Table S9** Univariable analysis of the sedation scale use for evaluate the sedation level

| Variables | Use sedation scale | | p-value |
| --- | --- | --- | --- |
|  | Yes n (%) | No n (%) |  |
| Estimated frequency of patients using mechanical ventilation |  |  | <0.001^a^ |
| <20% | 112 (8.1%) | 55 (3.9%) |  |
| 20-40% | 438 (31.5%) | 56 (4.0%) |  |
| 41-70% | 450 (32.4%) | 68 (4.9%) |  |
| 70% | 188 (13.5%) | 24 (1.7%) |  |
| Type of intensive care unit |  |  | <0.001^a^ |
| Mixed ICU | 820 (59.0%) | 112 (8.1%) |  |
| Medical | 161 (11.6%) | 47 (3.4%) |  |
| Surgical | 118 (8.5%) | 24 (1.7%) |  |
| Other | 87 (6.3%) | 20 (1.4%) |  |
| Number of beds in intensive care unit |  |  | <0.001^a^ |
| Up to 10 | 394 (28.3%) | 94 (6.8%) |  |
| 11-20 | 486 (34.9%) | 76 (5.5%) |  |
| >20 | 308 (22.1%) | 33 (2.4%) |  |
| There was an analgesia protocol in intensive care unit |  |  | <0.001^a^ |
| Yes | 845 (60.9%) | 88 (6.3%) |  |
| No | 317 (22.8%) | 107 (7.7%) |  |
| I don’t know | 25 (1.8%) | 7 (0.5%) |  |
| There was a sedation protocol in intensive care unit |  |  | <0.001^a^ |
| Yes | 886 (63.8%) | 76 (5.5%) |  |
| No | 284 (20.4%) | 116 (8.4%) |  |
| I don’t know | 18 (1.3%) | 9 (0.6%) |  |
| Frequency of discussion about sedation goals |  |  | <0.001^a^ |
| Daily | 950 (68.3%) | 110 (7.9%) |  |
| Sporadically | 215 (15.4%) | 83 (6.0%) |  |
| Never | 24 (1.7%) | 10 (0.7%) |  |
| Pharmacist participates in multidisciplinary rounds |  |  | 0.001^a^ |
| Yes | 392 (28.2 %) | 43 (3.1%) |  |
| No | 797 (57.2%) | 160 (11.5%) |  |
| Nurse:patient rate (daytime) |  |  | 0.002 |
| 1:1 | 46 (3.3 %) | 12 (0.9%) |  |
| 1:2 | 565 (40.8 %) | 92 (6.6%) |  |
| 1:3 | 362 (26.2 %) | 54 (3.9%) |  |
| 1:4 | 67 (4.8 %) | 26 (1.9%) |  |
| 1:5 | 65 (4.7 %) | 9 (0.7%) |  |
| >1:5 | 78 (5.6 %) | 8 (0.6%) |  |
| Nutritionist participates in multidisciplinary rounds |  |  | 0.003^a^ |
| Yes | 527 (37.9%) | 67 (4.8%) |  |
| No | 662 (47.5%) | 136 (9.8%) |  |
| Physiotherapist participates in multidisciplinary rounds |  |  | 0.003^a^ |
| Yes | 856 (61.5%) | 125 (9.0%) |  |
| No | 333 (23.9%) | 78 (5.6%) |  |
| Type of hospital |  |  | 0.009^a^ |
| Public hospital | 438 (31.5 %) | 90 (6.5%) |  |
| Private hospital | 361 (26.0 %) | 68 (4.9%) |  |
| University/Teaching hospital | 388 (27.9 %) | 45 (3.2%) |  |
| Experience as intensive care specialist |  |  | 0.03^a,^ |
| Up to 10 years | 549 (51.6 %) | 76 (7.1%) |  |
| Over 10 years | 365 (34.3 %) | 74 (7.0%) |  |
| Nurse participates in multidisciplinary rounds |  |  | 0.08^a^ |
| Yes | 1060 (76.1%) | 172 (12.4%) |  |
| No | 129 (9.3%) | 31 (2.2%) |  |
| Nurse:patient rate (nighttime) |  |  | 0.08^a^ |
| 1:1 | 33 (2.4%) | 7 (0.5%) |  |
| 1:2 | 478 (34.7%) | 78 (5.6%) |  |
| 1:3 | 402 (29.2%) | 66 (4.8%) |  |
| 1:4 | 95 (6.9%) | 27 (1.9%) |  |
| 1:5 | 56 (4.1%) | 11 (0.8%) |  |
| >1:5 | 114 (8.3%) | 11 (0.8%) |  |
| Time working in an intensive care |  |  | 0.16^a^ |
| Up to 10 years | 634 (45.5 %) | 97 (7.0%) |  |
| Over 10 years | 555 (39.9 %) | 106 (7.6%) |  |
| Daily rounds with an intensive care specialist |  |  | 0.17^a^ |
| Yes | 1106 (79.5%) | 183 (13.2%) |  |
| No | 82 (5.9%) | 20 (1.4%) |  |
| Intensive care specialist |  |  | 0.46 |
| Yes | 916 (65.8%) | 151 (10.9%) |  |
| No | 273 (19.6%) | 52 (3.7%) |  |
| Doctor participates on multidisciplinary rounds |  |  | 0.64 |
| Yes | 1182 (84.9%) | 202 (14.4%) |  |
| No | 7 (0.5%) | 2 (0.2%) |  |

^a^Variables included in logistic regression model

**Table S10** variables independently associated with sedation scale use for evaluate the sedation level

| Variables | OR | CI (2.5 % - 97.5 %) | p-value |
| --- | --- | --- | --- |
| Mixed intensive care unit | 1.79 | 1.15-2.79 | 0.01 |
| There was a sedation protocol | 4.17 | 1.43-12.17 | 0.001 |
| Up to 10 years of experience as intensive care specialist | 1.71 | 1.02-2.85 | 0.04 |
| Frequency of mechanical ventilation: > 70% | 4.87 | 2.43-9.76 | <0.001 |
| Frequency of mechanical ventilation: 40% - 70% | 3.45 | 1.99 -5.96 | <0.001 |
| Frequency of mechanical ventilation: 20% - 40% | 3.70 | 2.21-6.19 | <0.001 |

**Figure S3**: Sedative drugs avoided before the COVID-19 pandemic

**
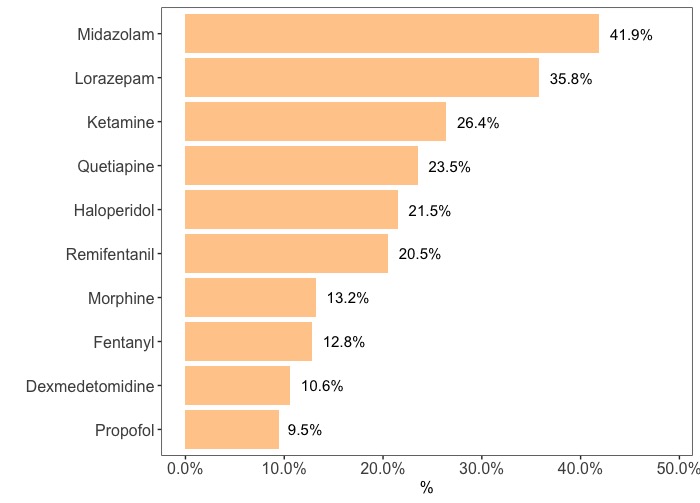
**

**Table S11** Sedation practices by continent before the COVID-19 pandemic

| Variables | Asia (n) | Europe (n) | South America (n) |
| --- | --- | --- | --- |
| **Frequency which doctors follow the sedation protocol** | | | |
| Always | 49% (72) | 66.7% (206) | 69.8% (337) |
| Sometimes | 51% (75) | 31.4% (97) | 29.0% (140) |
| Never | 0% (0) | 1.9% (6) | 1.2% (6) |
| **Physicians that regularly prescribe sedation to patients under MV^a^** | | | |
|  | 81.8% (139) | 92.8% (386) | 83% (627) |
| **Sedation Strategy^a^** | | | |
| Continuous sedation with daily interruption | 61.5% (104) | 25.2% (105) | 44% (332) |
| Continuous sedation with titration | 23.1% (39) | 73.8% (307) | 52.9% (399) |
| Intermittent bolus | 15.4% (26) | 1% (4) | 3.1% (23) |
| **Percentage of physicians that use sedation scale^a^** | | | |
|  | 72.9% (124) | 87% (362) | 87.8% (662) |
| **Sedation scale used** | | | |
| Ramsay^a^ | 29.8% (37) | 19.6% (69) | 14% (94) |
| SAS^a, b^ | 12.9% (16) | 5.25% (19) | 19% (126) |
| RASS^c,d^ | 73.4% (91) | 83.7% (303) | 73.1% (484) |
| Glasgow^d^ | 20.2% (25) | 21.3% (77) | 15.7% (104) |
| **Frequency of physicians that assess sedation level^a^** | | | |
| 1 | 7.1% (12) | 11.8% (48) | 13.5% (99) |
| 2 | 27.2% (46) | 21.1% (86) | 35.1% (258) |
| 3 | 39.6% (67) | 21.3% (87) | 21.4% (157) |
| >3 | 26.1% (44) | 45.8% (187) | 30% (221) |
| **Sedative drugs avoided or not used ^a^** | | | |
|  | 66.8% (112) | 61.1% (248) | 50% (370) |
| **Which sedative drug avoided or not used** | | | |
| Midazolam^a^ | 50.4% (57) | 39.2% (98) | 54.8% (148) |
| Lorazepam^a^ | 49.1% (56) | 34.8% (87) | 30.3% (112) |
| Haloperidol | 22.1% (25) | 25.6% (64) | 18.1% (67) |
| Morphine^d^ | 17.7% (20) | 16% (40) | 8.9% (33) |
| Fentanyl^a^ | 5.3% (6) | 28% (70) | 5.1% (19) |
| Propofol^d^ | 14.2% (16) | 5.2% (13) | 10.5% (39) |
| Remifentanil | 20.4% (23) | 17.2% (43) | 22.4% (83) |
| Dexmedetomidine | 8.9% (10) | 14.4% (36) | 8.7% (32) |
| Ketamine^d^ | 38.9% (44) | 26% (65) | 23.5% (87) |
| Quetiapine^a^ | 26.6% (30) | 36.8% (92) | 13.5% (50) |

^a^p≤0.001; ^b^ Sedation-Agitation Scale (SAS); ^c^ Richmond Agitation-Sedation Scale (RASS); ^d^p<0.05

**Figure S4**: **Strategies for improving ICU sedation practices**


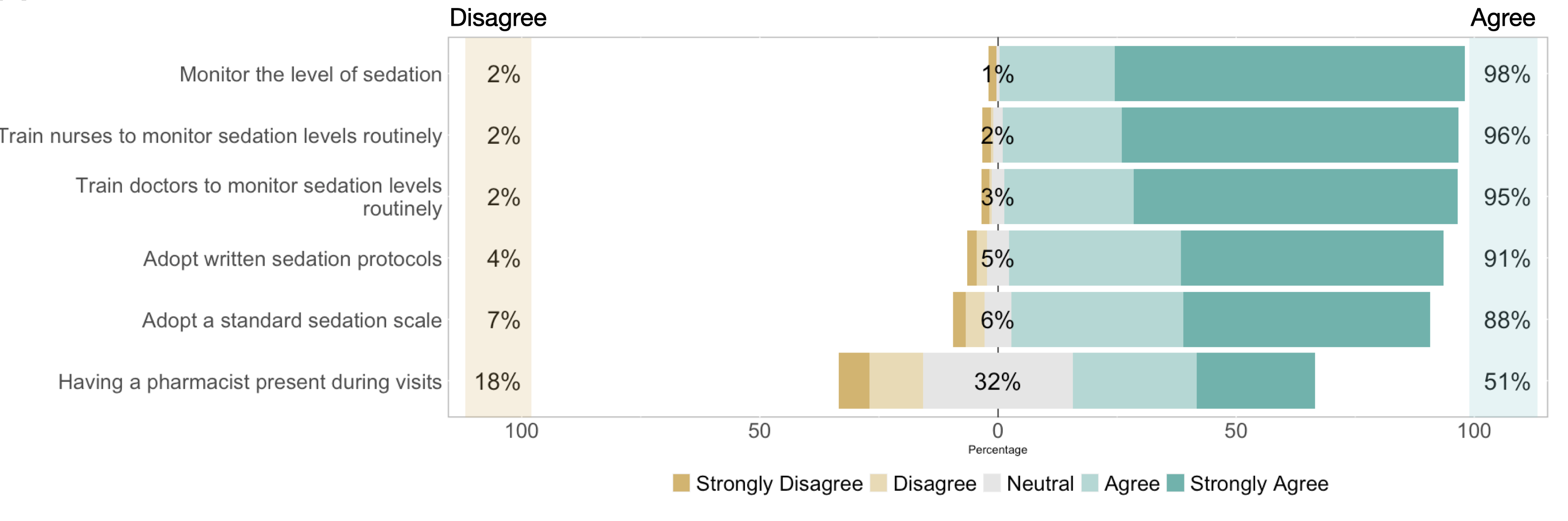


**Figure S5**: Opinion of physicians who have or not a pharmacist present during visits regarding the importance of this strategy in order to improve sedation practices before the COVID-19 pandemic


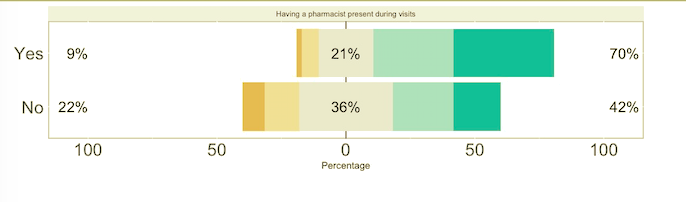


**
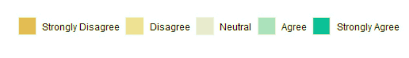
**

**Figure S6:** Use of physical restraint and of medication to induce sleep in patients on mechanical ventilation

**
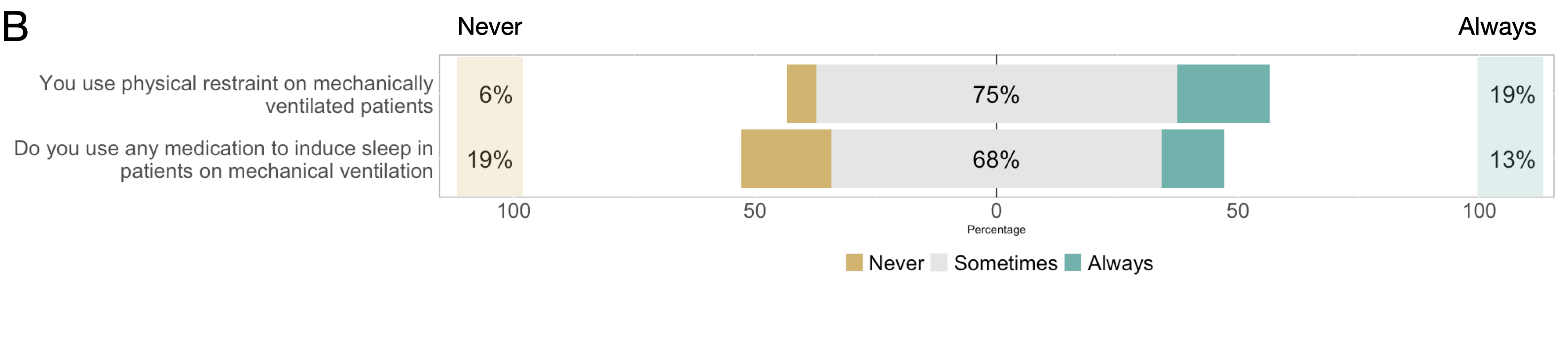
**

**Table S12** Univariable analysis use of mechanical restraint

| Variables (n) | Use Mechanical restraint | | p-value |
| --- | --- | --- | --- |
|  | Routine n (%) | Non-routine n (%) |  |
| Nurse:patient rate (daytime) |  |  | <0.001 |
| 1:1 | 1 (0.1 %) | 51 (3.9 %) |  |
| 1:2 | 93 (7.0 %) | 537 (40.6 %) |  |
| 1:3 | 107 (8.0 %) | 289 (21.8 %) |  |
| 1:4 | 17 (1.3 %) | 74 (5.6 %) |  |
| 1:5 | 15 (1.1 %) | 55 (4.2 %) |  |
| >1:5 | 20 (1.5 %) | 65 (4.9 %) |  |
| Nurse:patient rate (nighttime) |  |  | 0.001^a^ |
| 1:1 | 1 (0.1 %) | 33 (2.5 %) |  |
| 1:2 | 82 (6.2 %) | 452 (34.2 %) |  |
| 1:3 | 109 (8.3 %) | 339 (25.7 %) |  |
| 1:4 | 21 (1.6 %) | 96 (7.3 %) |  |
| 1:5 | 13 (1.0 %) | 50 (3.8 %) |  |
| >1:5 | 26 (1.9 %) | 97 (7.4 %) |  |
| Time working in an intensive care unit |  |  | 0.002^a^ |
| Up to 10 years | 154 (11.6 %) | 537 (40.3 %) |  |
| Over 10 years | 100 (7.5 %) | 540 (40.6 %) |  |
| Intensive care specialist |  |  | 0.006^a^ |
| Yes | 180 (13.5 %) | 851 (63.9%) |  |
| No | 74 (5.6 %) | 226 (17.0 %) |  |
| Frequency of discussion about sedation goals |  |  | 0.01^a^ |
| Daily | 188 (14.1 %) | 827 (62.2 %) |  |
| Sporadically | 53 (4.0 %) | 229 (17.2 %) |  |
| Never | 13 (1.0 %) | 20 (1.5 %) |  |
| Nurse participates in multidisciplinary rounds |  |  | 0.01 |
| Yes | 214 (16.1 %) | 967 (72.7 %) |  |
| No | 40 (3.0 %) | 110 (8.3 %) |  |
| Experience as intensive care specialist |  |  | 0.04^a,b^ |
| Up to 10 years | 117 (11.4 %) | 484 (47.1 %) |  |
| Over 10 years | 62 (6.0 %) | 365 (35.5 %) |  |
| Estimated frequency of patients using mechanical ventilation |  |  | 0.04^a^ |
| <20% | 19 (1.4 %) | 137 (10.3 %) |  |
| 20-40% | 86 (6.5 %) | 389 (29.2 %) |  |
| 41-70% | 101 (7.6 %) | 395 (27.7 %) |  |
| >70% | 48 (3.6 %) | 156 (11.7 %) |  |
| Daily rounds with an intensive care specialist |  |  | 0.05^a^ |
| Yes | 228 (17.1 %) | 1006(75.6%) |  |
| No | 26 (2.0 %) | 70 (5.3 %) |  |
| Nutritionist participates in multidisciplinary rounds |  |  | 0.10^a^ |
| Yes | 97 (7.3 %) | 447 (35.6 %) |  |
| No | 157 (11.8 %) | 603 (45.3 %) |  |
| There was a sedation protocol in intensive care unit |  |  | 0.13^a^ |
| Yes | 164 (12.4 %) | 761 (57.3 %) |  |
| No | 84 (6.3 %) | 294 (22.2 %) |  |
| I don’t know | 6 (0.4 %) | 18 (1.4 %) |  |
| There was an analgesia protocol in intensive care unit |  |  | 0.23 |
| Yes | 159 (12 %) | 735 (55.3 %) |  |
| No | 88 (6.6 %) | 318 (24.0 %) |  |
| I don’t know | 6 (0.4 %) | 22 (1.7 %) |  |
| Number of beds in intensive care unit |  |  | 0.26 |
| Up to 10 | 90 (6.8 %) | 375 (28.2 %) |  |
| 11-20 | 111 (8.3 %) | 427 (32.1 %) |  |
| >20 | 53 (4.0 %) | 274 (20.6%) |  |
| Type of hospital |  |  | 0.32 |
| Public hospital | 99 (7.4%) | 404 (30.4 %) |  |
| Private hospital | 84 (6.3 %) | 321 (24.2 %) |  |
| University/Teaching hospital | 71 (5.3 %) | 351 (26.4 %) |  |
| Doctor participates in multidisciplinary rounds |  |  | 0.37 |
| Yes | 251 (18.9 %) | 1071(80.5%) |  |
| No | 3 (0.2 %) | 6 (0.4%) |  |
| Pharmacist participates in multidisciplinary rounds |  |  | 0.38 |
| Yes | 86 (6.5 %) | 332 (24.9 %) |  |
| No | 168 (12.6 %) | 745 (56.0 %) |  |
| Type of intensive care unit |  |  | 0.83 |
| Mixed ICU | 169 (12.7 %) | 725 (54.6 %) |  |
| Medical | 38 (2.9 %) | 164 (12.3 %) |  |
| Surgical | 24 (1.8 %) | 107 (8.1 %) |  |
| Other | 23 (1.7 %) | 79 (5.9 %) |  |
| Physiotherapist participates in multidisciplinary rounds |  |  | 0.93 |
| Yes | 180 (13.5 %) | 758 (56.9 %) |  |
| No | 74 (5.6 %) | 319 (24.0 %) |  |

^a^Variables included in logistic regression model; ^b^ Variable excluded in logistic regression model due to multicollinearity

**Table S13** variables independently associated with mechanical restraint use

| Variables | OR | CI (2.5 % - 97.5 %) | p-value |
| --- | --- | --- | --- |
| Nurse:patient rate (daytime) of 1:1 | 33.12 | 2.51-435.72 | 0.007 |
| Nurse:patient rate (daytime) of 1:2 | 4.62 | 1.34-15.90 | 0.01 |

**Figure S7** Tools used for delirium assessment before the COVID-19 pandemic


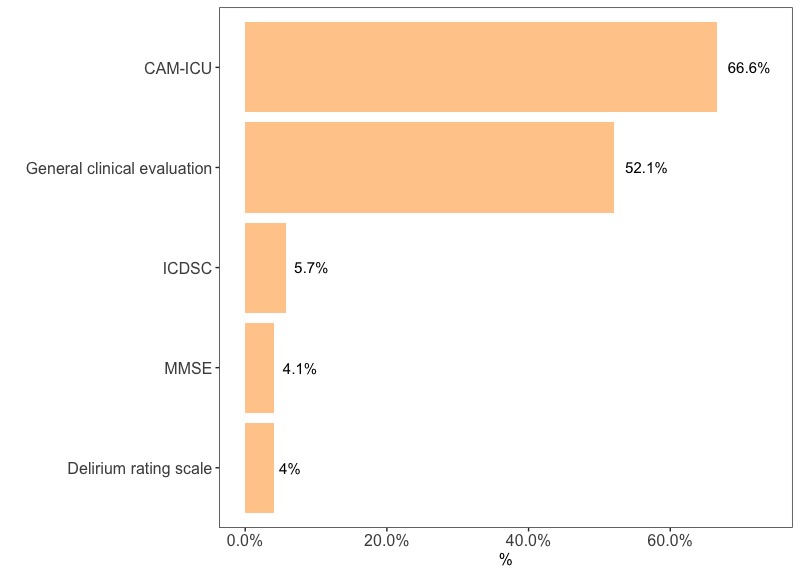


**CAM-ICU:** Confusion Assessment Method for the Intensive Care Unit; **ICDSC:** Intensive Care Delirium Screening Checklist; **MMSE:** Mini Mental State Examination;

**Figure S8:** Drugs used for delirium management before the COVID-19 pandemic

**
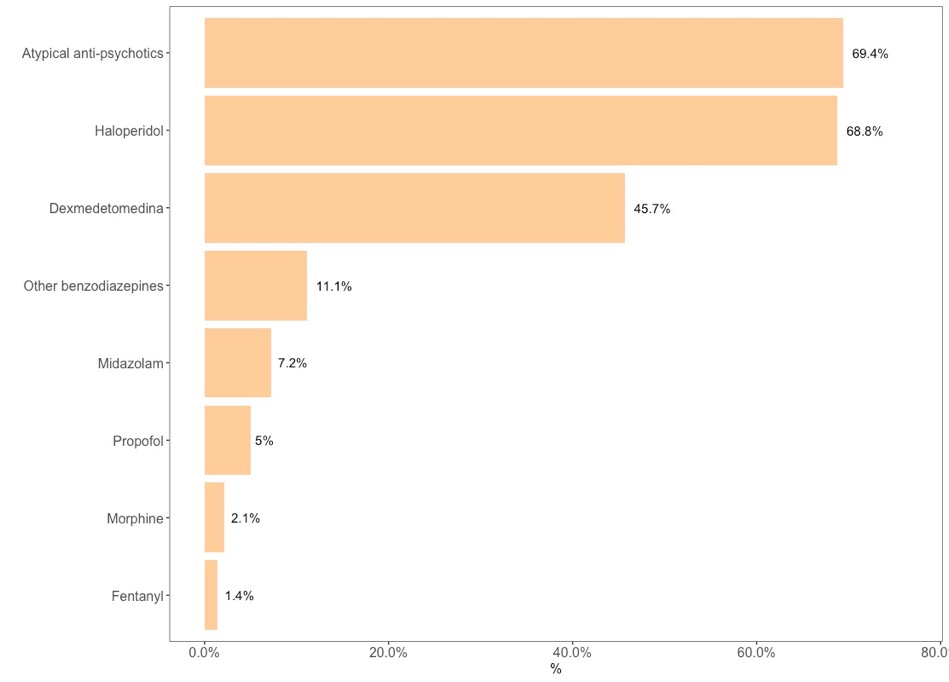
**

**Figure S9:** Strategies for delirium hypoactive treatment before the COVID-19 pandemic

**
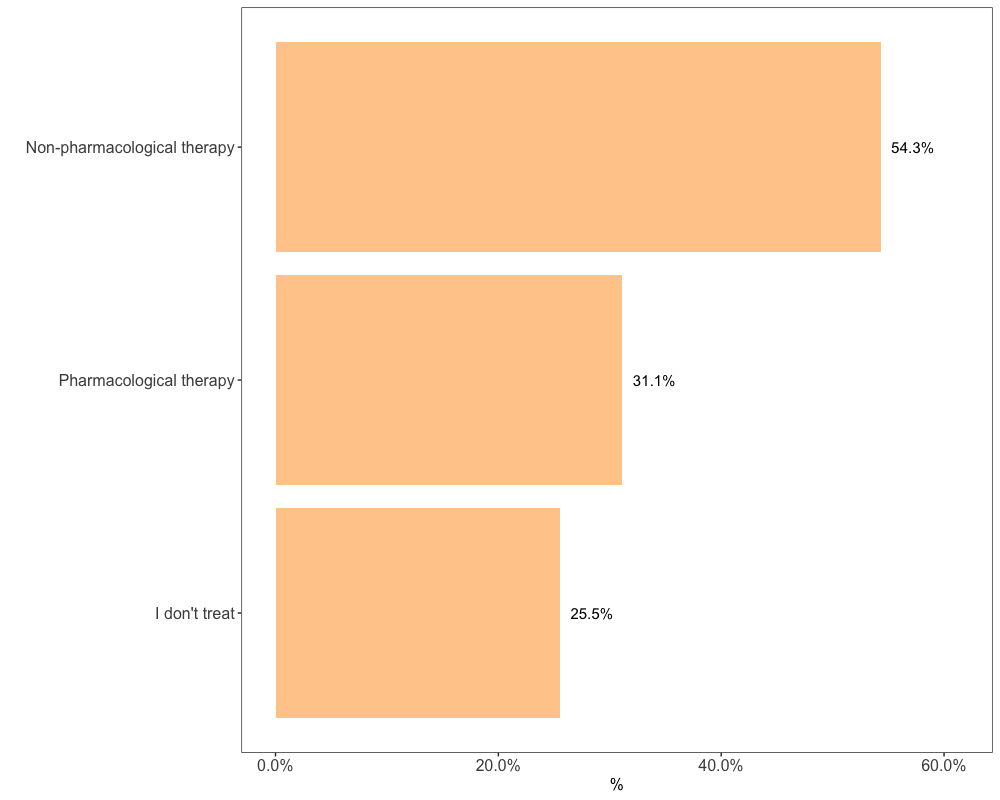
**

**Figure S10:** Non-pharmacological therapies used for delirium hypoactive management before the COVID-19 pandemic


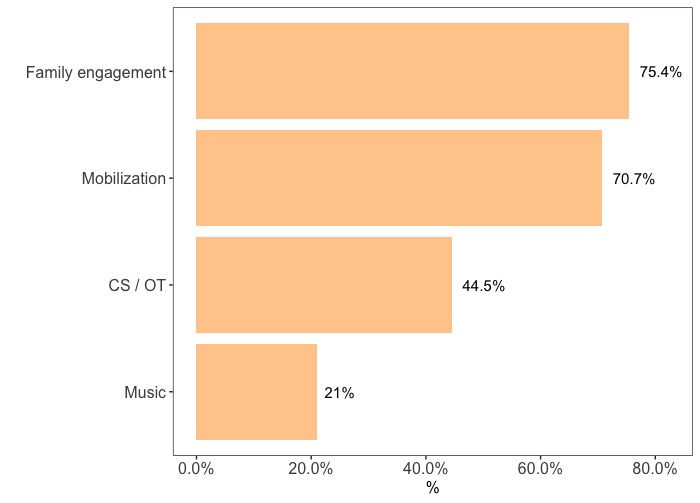


**CS/OT:** Cognitive Stimulation/ Occupational Therapy;

**Table S14** Univariable analysis delirium investigate

| Variables | Delirium investigation | | p-value |
| --- | --- | --- | --- |
|  | Yes n (%) | No n (%) |  |
| Physiotherapist participates in multidisciplinary rounds |  |  | <0.001^a^ |
| Yes | 808 (61.4%) | 120 (9.1%) |  |
| No | 304 (23.1%) | 84 (6.4%) |  |
| There was a sedation protocol in intensive care unit |  |  | <0.001^a^ |
| Yes | 806 (61.4%) | 111 (8.4%) |  |
| No | 285 (21.7%) | 86 (6.6%) |  |
| I don’t know | 18 (1.4%) | 6 (0.5%) |  |
| Frequency of discussion about sedation goals |  |  | <0.001^a^ |
| Daily | 891 (67.8 %) | 115 (8.7%) |  |
| Sporadically | 199 (15.1 %) | 77 (5.9%) |  |
| Never | 21 (1.6 %) | 12 (0.9%) |  |
| Know delirium frequency |  |  | <0.001^a^ |
| Yes | 511 (38.8 %) | 25 (1.9%) |  |
| No | 601 (45.7%) | 179 (13.6%) |  |
| Daily rounds with an intensive care specialist |  |  | 0.001^a^ |
| Yes | 1041 (79.2%) | 178 (13.5%) |  |
| No | 70 (5.3%) | 26 (2.0%) |  |
| Type of hospital |  |  | 0.01^a^ |
| Public hospital | 408 (31.0 %) | 89 (6.8%) |  |
| Private hospital | 333 (25.3 %) | 68 (5.2%) |  |
| University hospital / Teaching hospital | 370 (28.1 %) | 47 (3.6%) |  |
| Pharmacist participates in multidisciplinary rounds |  |  | 0.06^a^ |
| Yes | 366 (27.8%) | 47 (3.6%) |  |
| No | 746 (56.7%) | 157 (11.9%) |  |
| Type of intensive care unit |  |  | 0.02^a^ |
| Mixed ICU | 764 (58.1%) | 118 (9.0%) |  |
| Medical | 160 (12.2%) | 41 (3.1%) |  |
| Surgical | 105 (8.0%) | 24 (1.8%) |  |
| Other | 82 (6.3%) | 20 (1.5%) |  |
| Nurse participates in multidisciplinary rounds |  |  | 0.02^a^ |
| Yes | 997 (75.8%) | 171 (13.0%) |  |
| No | 115 (8.7%) | 33 (2.5%) |  |
| Nutritionist participates in multidisciplinary rounds |  |  | 0.02^a^ |
| Yes | 489 (37.2%) | 72 (5.5%) |  |
| No | 623 (47.3%) | 132 (10.0%) |  |
| Doctor participates in multidisciplinary rounds |  |  | 0.03^a^ |
| Yes | 1107 (84.2%) | 200 (15.2%) |  |
| No | 5 (0.4%) | 4 (0.2%) |  |
| Estimated frequency of patients using mechanical ventilation |  |  | 0.04^a^ |
| <20% | 120 (9.1%) | 33 (2.5%) |  |
| 20-40% | 401 (30.5%) | 68 (5.2%) |  |
| 41-70% | 411 (31.3%) | 81 (6.1%) |  |
| >70% | 179 (13. 6%) | 22 (1.7%) |  |
| Number of beds in intensive care unit |  |  | 0.15^a^ |
| Up to 10 | 377 (28.7%) | 83 (6.3%) |  |
| 11-20 | 454 (34.5%) | 78 (5.9%) |  |
| >20 | 280 (21.3%) | 43 (3.3%) |  |
| Nurse:patient rate (daytime) |  |  | 0.16^a^ |
| 1:1 | 44 (3.4 %) | 7 (0.5%) |  |
| 1:2 | 525 (40.1%) | 97 (7.4%) |  |
| 1:3 | 333 (25.4 %) | 62 (4.7%) |  |
| 1:4 | 68 (5.2 %) | 21 (1.6%) |  |
| 1:5 | 62 (4.7 %) | 6 (0.5%) |  |
| >1:5 | 75 (5.7 %) | 10 (0.8%) |  |
| Time working in an intensive care unit |  |  | 0.25 |
| Up to 10 years | 565 (42.9%) | 113 (8.6%) |  |
| Over 10 years | 547 (41.6%) | 91 (6.9%) |  |
| Experience as intensive care specialist |  |  | 0.48 |
| Up to 10 years | 492 (48.4%) | 98 (9.7%) |  |
| Over 10 years | 363 (35.7%) | 63 (6.2%) |  |
| Intensive care specialist |  |  | 0.51 |
| Yes | 857 (65.1%) | 162 (12.3%) |  |
| No | 255 (19.4%) | 42 (3.2%) |  |
| Nursing:patient rate (nighttime) |  |  | 0.59 |
| 1:1 | 30 (2.2%) | 4 (0.3%) |  |
| 1:2 | 444 (34%) | 82 (6.3%) |  |
| 1:3 | 371 (28.4%) | 77 (5.9%) |  |
| 1:4 | 96 (7.4%) | 19 (1.5%) |  |
| 1:5 | 55 (4.2%) | 7 (0.5%) |  |
| >1:5 | 107 (8.2%) | 14 (1.1%) |  |

^a^Variables included of the logistic regression model;

**Table S15** variable independently associated with delirium investigate

| Variables | OR | CI (2.5 % - 97.5 %) | p-value |
| --- | --- | --- | --- |
| Physiotherapist participate on multidisciplinary rounds | 1.77 | 1.14-2.75 | 0.01 |
| To know delirium frequency in their unit | 5.50 | 3.47-8.71 | <0.001 |
| University Hospital/Teaching hospital | 1.93 | 1.21-3.08 | 0.005 |

**Table S16** Univariable analysis delirium investigate using structured tools

| Variables | Use structured tools | | p-value |
| --- | --- | --- | --- |
|  | Yes n (%) | No n( %) |  |
| Know delirium frequency |  |  | <0.001^a^ |
| Yes | 429 (38.6%) | 82 (7.4%) |  |
| No | 397 (35.7%) | 204 (18.3%) |  |
| Pharmacist participates in multidisciplinary rounds |  |  | <0.001^a^ |
| Yes | 296 (26.6%) | 70 (6.3%) |  |
| No | 530 (47.7%) | 216 (19.4%) |  |
| Number of beds in intensive care unit |  |  | <0.001^a^ |
| Up to 10 | 257 (23.1%) | 120 (10.8%) |  |
| 11-20 | 339 (30.5%) | 115 (10.4%) |  |
| >20 | 229 (20.6%) | 51 (4.6%) |  |
|  |  |  |  |
| There was a sedation protocol in intensive care unit |  |  | 0.002^a^ |
| Yes | 619 (55.8%) | 186 (16.8%) |  |
| No | 193 (17.4%) | 93 (8.4%) |  |
| I don’t know | 11 (1.0%) | 7 (0.6%) |  |
| Nutritionist participates in multidisciplinary rounds |  |  | 0.02^a^ |
| Yes | 381 (34.3%) | 109 (9.8%) |  |
| No | 445 (40.0%) | 177 (15.9%) |  |
| Estimated frequency of patients using mechanical ventilation |  |  | 0.003^a^ |
| <20% | 75 (6.8 %) | 46 (4.1%) |  |
| 20-40% | 315 (28.4%) | 86 (7.7%) |  |
| 41-70% | 303 (27.3%) | 107 (9.6%) |  |
| >70% | 132 (11.9%) | 47 (4.2%) |  |
| Frequency of discussion about sedation goals |  |  | 0.05^a^ |
| Daily | 661 (59.5%) | 230 (20.7%) |  |
| Sporadically | 153 (13.8%) | 46 (4.1%) |  |
| Never | 11 (1.0%) | 10 (0.9%) |  |
| Daily rounds with an intensive care specialist |  |  | 0.06^a^ |
| Yes | 780 (70.2%) | 261 (23.5%) |  |
| No | 45 (4.1%) | 25 (2.2%) |  |
| Experience as intensive care specialist care |  |  | 0.06 |
| Up to 10 years | 377 (44.1%) | 114 (13.3%) |  |
| Over 10 years | 258 (30.2%) | 106 (12.4%) |  |
| Type of hospital |  |  | 0.09^a^ |
| Public hospital | 287 (25.8%) | 120 (10.8%) |  |
| Private hospital | 254 (22.9%) | 80 (7.2%) |  |
| University hospital / Teaching hospital | 284 (25.6%) | 86 (7.7%) |  |
| Nurse:patient rate (nighttime) |  |  | 0.09^a^ |
| 1:1 | 25 (2.3%) | 5 (0.4%) |  |
| 1:2 | 330 (29.9%) | 113 (10.3%) |  |
| 1:3 | 258 (23.4%) | 113 (10.3%) |  |
| 1:4 | 76 (6.9%) | 20 (1.8%) |  |
| 1:5 | 43 (3.9%) | 12 (1.1%) |  |
| >1:5 | 86 (7.8%) | 21 (1.9%) |  |
| Doctor participates in multidisciplinary rounds |  |  | 0.11^a^ |
| Yes | 824 (74.1%) | 238 (25.4%) |  |
| No | 2 (0.2%) | 3 (0.3%) |  |
| Nurse:patient rate (daytime) |  |  | 0.14^a^ |
| 1:1 | 35 (3.2 %) | 9 (0.8%) |  |
| 1:2 | 388 (35.0%) | 137 (12.4%) |  |
| 1:3 | 235 (21.2%) | 98 (8.9%) |  |
| 1:4 | 51 (4.6%) | 17 (1.5%) |  |
| 1:5 | 48 (4.3%) | 14 (1.3%) |  |
| >1:5 | 64 (5.8%) | 11 (1.0%%) |  |
| Physiotherapist participates in multidisciplinary rounds |  |  | 0.38 |
| Yes | 594 (53.4%) | 214 (19.2%) |  |
| No | 232 (20.9%) | 72 (6.5%) |  |
| Type of intensive care unit |  |  | 0.43 |
| Mixed ICU | 575 (51.8%) | 189 (17.0%) |  |
| Medical | 120 (10.8%) | 40 (3.6%) |  |
| Surgical | 74 (6.7%) | 31 (2.8%) |  |
| Other | 56 (5.0%) | 26 (2.3%) |  |
| Time working in an intensive care |  |  | 0.83 |
| Up to 10 years | 421 (37.8%) | 143 (12.9%) |  |
| Over 10 years | 405 (36.4%) | 143 (12.9%) |  |
| Nurse participates in multidisciplinary rounds |  |  | 0.98 |
| Yes | 740 (66.5%) | 257 (23.1%) |  |
| No | 86 (7.7%) | 29 (2.6%) |  |
| Intensive care specialist |  |  | 0.98 |
| Yes | 636 (57.2 %) | 221(18.9%) |  |
| No | 190 (17.1%) | 65 (5.8%) |  |

^a^Variables included of the logistic regression model;

**Table S17** variable independently associated with delirium investigate using structured tools

| Variables | OR | CI (2.5 % - 97.5 %) | p-value |
| --- | --- | --- | --- |
| Frequency of mechanical ventilation: > 70% | 2.23 | 1.11 - 4.45 | 0.02 |
| Have information about delirium frequency in their unit | 5.41 | 3.41 - 8.5 | <0.001 |
| University Hospital / Teaching hospital | 1.77 | 1.11 – 2.82 | 0.02 |

**Figure S11:** Early mobilization techniques used before the COVID-19 pandemic

**
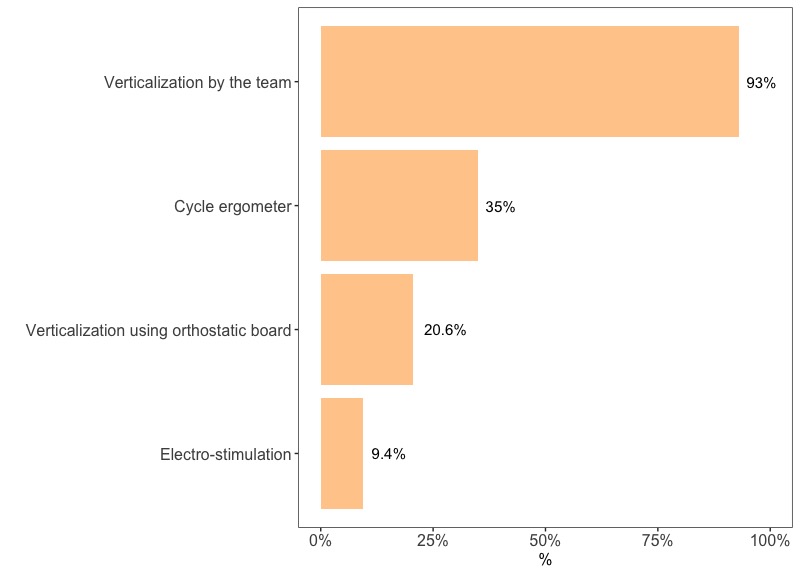
**

**Figure S12** Drugs used to induce sleep in patients on mechanical ventilation before the COVID-19 pandemic


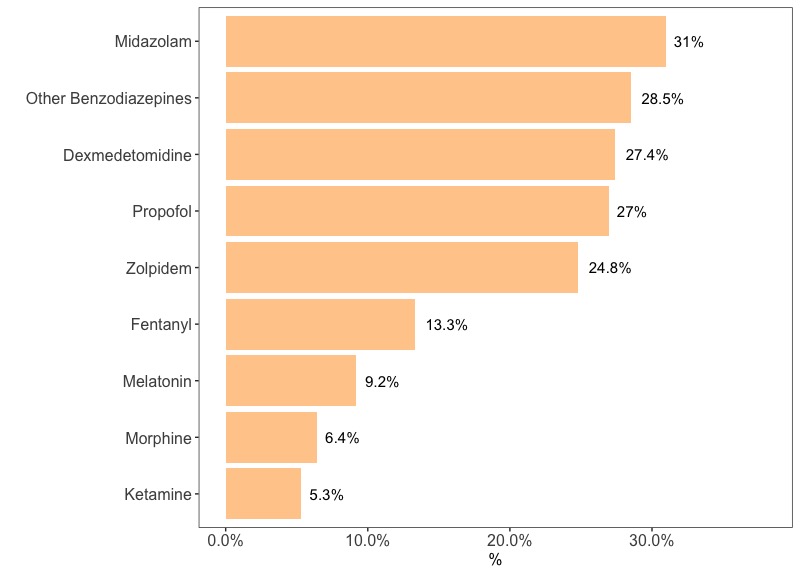


**Figure S13:** Non-pharmacological therapies used to induce sleep in patients on mechanical ventilation before the COVID-19 pandemic

**
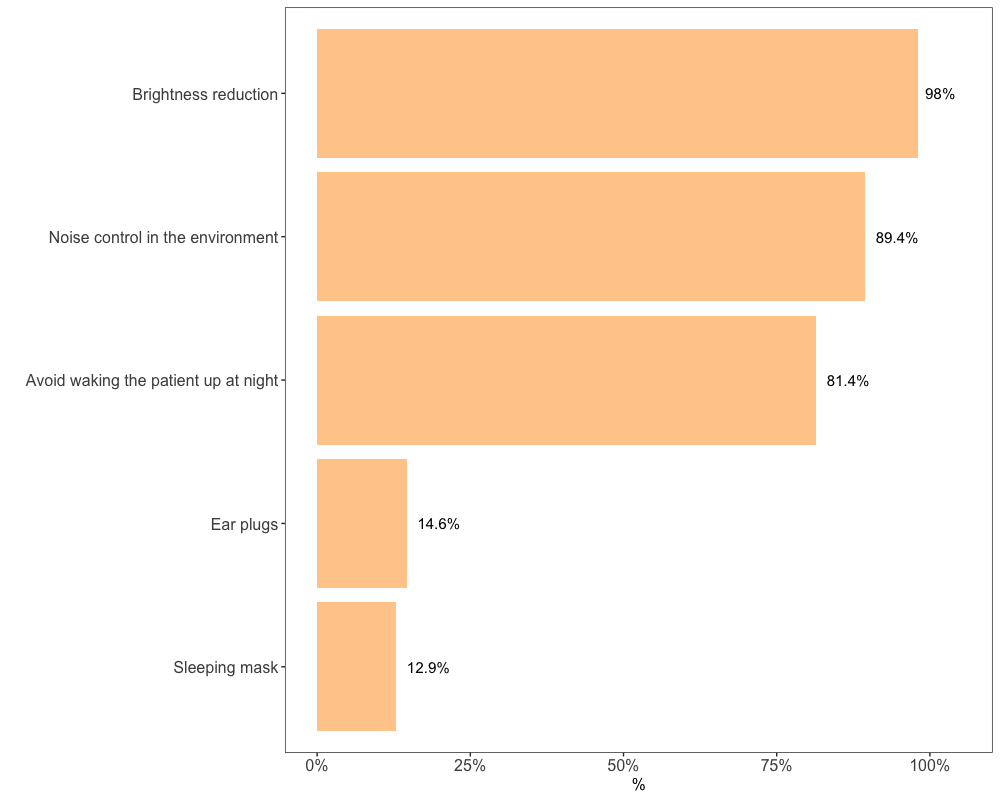
**

**Table S18** Sedation practices before and during the COVID-19 pandemic

| Variables | Before the COVID-19 pandemic | During the COVID-19 pandemic |
| --- | --- | --- |
| **Frequency which doctors follow the sedation protocol^a^** | | |
| Always | 635 (65.5%) | 141 (73.8%) |
| Sometimes | 326 (33.6%) | 50 (26.2%) |
| Never | 9 (0.9%) | 0 (0.0%) |
| **Physicians that regularly prescribe sedation to patients under MV^b,c^** | | |
|  | 1199 (86.1%) | 252 (94.0)% |
| **Sedation Strategy^b^** | | |
| Continuous sedation with titration | 763 (54.9%) | 184 (68.7%) |
| Continuous sedation with daily interruption | 571 (41.1%) | 80 (29.9%) |
| Intermittent bolus | 56 (4.0%) | 4 (1.5%) |
| **Percentage of physicians that use sedation scale** | | |
|  | 1189 (85.4%) | 232 (85.4%) |
| **Sedation scale used** |  |  |
| RASS | 911 (76.6%) | 177 (76.3%) |
| Glasgow^a^ | 212 (17.8%) | 22 (9.5%) |
| Ramsay | 206 (17.3%) | 45 (19.1%) |
| SAS | 166 (14.0%) | 31 (13.3%) |
| **Frequency that physicians assess sedation level per day** | | |
| 1 | 166 (12.2%) | 32 (12.0%) |
| 2 | 404 (29.6%) | 64 (24.0%) |
| 3 | 316 (23.2%) | 56 (21.0%) |
| >3 | 477 (35.0%) | 115 (43.0%) |
| **Frequency that sedations goals are discussed during rounds^b^** | | |
| Daily | 1060 (76.2%) | 227 (77.5%) |
| Sporadically | 298 (21.4%) | 34 (20%) |
| Never | 34 (2.4%) | 7 (2.5%) |
| **Drugs usually used for sedation** | | |
| Midazolam^a^ | 1159 (84.7%) | 244 (91.4%) |
| Lorazepam | 166 (12.1%) | 23 (8.6%) |
| Haloperidol | 337 (24.7%) | 57 (21.3%) |
| Morphine | 286 (20.9%) | 50 (18.7%) |
| Fentanyl | 632 (46.2%) | 107 (40.1%) |
| Propofol^b^ | 1080 (79.0%) | 236 (88.4%) |
| Remifentanil | 162 (11.9%) | 40 (15.0%) |
| Dexmedetomidine | 890 (65.1%) | 173 (64.8%) |
| Ketamine^b^ | 384 (28.1%) | 103 (38.6%) |
| Quetiapine^a^ | 282 (20.6%) | 73 (27.3%) |
| **Sedative drugs used for sedation in patients with septic shock** | | |
| Midazolam | 856 (62.7%) | 184 (68.9%) |
| Lorazepam | 30 (2.2%) | 5 (1.9%) |
| Haloperidol | 42 (3.1%) | 6 (2.2%) |
| Morphine | 159 (11.6%) | 22 (8.2%) |
| Fentanyl | 757 (55.4%) | 135 (50.6%) |
| Propofol | 560 (41.0%) | 118 (44.2%) |
| Remifentanil | 163 (11.9%) | 39 (14.6%) |
| Dexmedetomidine | 331 (24.2%) | 53 (19.9%) |
| Ketamine^a^ | 324 (23.7%) | 92 (34.5%) |
| Quetiapine | 38 (2.8%) | 10 (3.7%) |
| **Sedative drugs used for sedation in patients with ARDS^f^** | | |
| Midazolam | 977 (71.5%) | 204 (76.4%) |
| Lorazepam | 36 (2.6%) | 6 (2.2%) |
| Haloperidol | 32 (2.3%) | 11 (4.1%) |
| Morphine | 201 (14.7%) | 41 (15.4%) |
| Fentanyl | 837 (61.2%) | 153 (57.3%) |
| Propofol^b^ | 835 (61.1%) | 197 (73.8%) |
| Remifentanil | 182 (13.3%) | 48 (18.0%) |
| Dexmedetomidine | 320 (23.4%) | 73 (27.3%) |
| Ketamine^b^ | 306 (22.4%) | 90 (33.7%) |
| Quetiapine^b^ | 39 (2.9%) | 21 (7.9%) |
| **Sedative drugs used for sedation in patients with NIMV^g^** | | |
| Midazolam | 163 (11.9%) | 32 (12.0%) |
| Lorazepam | 99 (7.2%) | 26 (9.7%) |
| Haloperidol | 448 (32.8%) | 93 (34.8%) |
| Morphine | 262 (19.2%) | 56 (21.0%) |
| Fentanyl^a^ | 196 (14.3%) | 25 (9.4%) |
| Propofol | 194 (14.2%) | 45 (16.9%) |
| Remifentanil | 70 (5.1%) | 19 (7.1%) |
| Dexmedetomidine | 882 (64.5%) | 171 (64.0%) |
| Ketamine | 158 (11.6%) | 41 (15.4%) |
| Quetiapine | 308 (22.5%) | 74 (27.7%) |
| **Which sedative drug avoided or not used** | | |
| Midazolam^a^ | 321 (41.9%) | 45 (32.1%) |
| Lorazepam | 275 (35.8%) | 57 (40.7%) |
| Haloperidol | 165 (21.5%) | 35 (25.0%) |
| Morphine | 101 (13.2%) | 19 (13.6%) |
| Fentanyl | 98 (12.8%) | 21 (15.0%) |
| Propofol | 73 (9.5%) | 11 (7.9%) |
| Remifentanil | 157 (20.5%) | 33 (23.6%) |
| Dexmedetomidine | 81 (10.6%) | 10 (7.1%) |
| Ketamine^a^ | 203 (26.4%) | 23 (16.4%) |
| Quetiapine | 180 (23.5%) | 32 (22.9%) |
| **Use physical restraint on mechanically ventilated patients** | | |
| Always | 254 (19.1%) | 67 (20.2%) |
| Sometimes | 995 (74.8%) | 178 (73.6%) |
| Never | 82 (6.2%) | 17 (6.2%) |

^a^p<0.05; ^b^p≤0.001; ^c^Mechanical Ventilation (MV); ^d^Richmond Agitation Sedation Scale (RASS); ^e^Sedation*-*Agitation Scale (SAS); ^f^acute respiratory distress syndrome (ARDS); ^g^non-invasive mechanical ventilation (NIMV)

**Table S19:** Analgesia practices before and during the COVID-19 pandemic

| Variables | Before the COVID-19 pandemic | During the COVID-19 pandemic |
| --- | --- | --- |
| **Percentage of physicians that assess pain in patients that are able to communicate** | | |
|  | 1277 (86.6%) | 246 (84.2%) |
| **Tools to assess pain in patients that are able to communicate** | | |
| Visual analogic scale | 786 (61.5%) | 147 (59.8%) |
| BPS^ab^ | 442 (34.6%) | 116 (47.2%) |
| CPOT^c^ | 109 (8.5%) | 14 (5.7%) |
| Unstructured evaluation | 224 (17.5%) | 39 (15.9%) |
| Numerical oral scale | 674 (52.7%) | 143 (58.1%) |
| **Percentage of physicians that assess pain in patients that are unable to communicate** | | |
|  | 971 (67.4%) | 202 (71.4%) |
| **Tools to assess pain in patients unable to communicate** | | |
| Visual analogic scale^b^ | 296 (30.4%) | 45 (22.3%) |
| BPS^d^ | 469 (48.2%) | 135 (66.8%) |
| CPOT | 202 (20.8%) | 32 (15.8%) |
| Unstructured evaluation | 224 (22.2%) | 37 (18.3%) |
| **Drugs usually used for analgesia** | | |
| Midazolam | 224 (17.0%) | 47 (16.7%) |
| Dipyrone^b^ | 663 (40.0%) | 111 (39.5%) |
| Morphine | 1037 (72.0%) | 217 (77.2%) |
| Fentanyl^b^ | 1129 (78.3%) | 194 (69%) |
| Remifentanil | 269 (18.7%) | 67 (23.8%) |
| Tramadol^b^ | 850 (59.0%) | 141 (50.2%) |
| Gabapentin | 308 (21.4%) | 63 (22.4%) |
| Propofol | 229 (15.9%) | 33 (11.7%) |
| Dexmedetomidine | 512 (35.5%) | 88 (31.3%) |
| Non-steroidal^b^ | 566 (39.3%) | 90 (32.0%) |
| Paracetamol | 959 (66.6%) | 192 (68.3%) |
| Nefopam^d^ | 246 (17.1%) | 86 (30.6%) |
| Ketamine^d^ | 554 (38.4%) | 152 (54.1%) |
| **Percentage of physicians that use non-pharmacological therapy for pain management** | | |
|  | 584 (41.1%) | 119 (43.1%) |
| **Types of non-pharmacological pain therapy used** | | |
| Massage | 240 (41.1%) | 55 (46.2%) |
| Hypnosis^d^ | 64 (11.0%) | 30 (25.2%) |
| Cybertherapy | 9 (1.5%) | 2 (1.7%) |
| Relaxation techniques | 148 (25.3%) | 33 (27.7%) |
| Ice pac | 194 (33.2%) | 42 (34.7%) |
| Music therapy | 254 (43.5%) | 51 (42.5%) |

^a^Behavioral Pain Scale (BPS); ^b^ p<0.05; ^c^Critical Care Pain Observation Tool (CPOT); ^d^p<0.001

**Table S20:** Delirium assessment and management before and during the COVID-19 pandemic

| Variables | Before the COVID-19 pandemic | During the COVID-19 pandemic |
| --- | --- | --- |
| **The presence of delirium is investigated at least once a day** | | |
|  | 1060 (95.4%) | 205 (95.4%) |
| **The physicians assess delirium in...** | | |
| All patients | 427 (38.5%) | 84 (38.9%) |
| Only patients with Clinical suspicion | 682 (61.5%) | 132 (61.1%) |
| **Which instrument is used to assess the presence of delirium** | | |
| General clinical evaluation | 577 (52.1%) | 125 (57.9%) |
| CAM-ICU^a^ | 741 (66.6%) | 136 (63.0%) |
| Delirium rating scale | 45 (4.0%) | 6 (2.8%) |
| ICDSC^b^ | 63 (5.7%) | 7 (3.2%) |
| MMSE^c^ | 52 (4.7%) | 10 (4.6%) |
| **Which drugs are usually used to treat delirium** | | |
| Midazolam | 95 (7.2%) | 20 (7.7%) |
| Other benzodiazepines^d^ | 146 (11.1%) | 45 (17.3%) |
| Haloperidol | 905 (68.8%) | 173 (66.5%) |
| Morphine | 28 (2.1%) | 11 (4.2%) |
| Fentanyl | 19 (1.4%) | 5 (1.9%) |
| Propofol | 66 (5.0%) | 10 (3.8%) |
| Dexmedetomidine | 601 (45.7%) | 121 (46.5%) |
| AA^e^ | 913 (69.4%) | 181 (69.6%) |
| I don't use drugs for delirium treatment | 74 (5.6%) | 15 (5.8%) |
| **Delirium hypoactive treatment** | | |
| Non-pharmacological therapy | 707 (54.3%) | 152 (58.5%) |
| Pharmacological therapy | 405 (31.1%) | 78 (30.1%) |
| No treatment | 332 (25.5%) | 73 (28.1%) |
| **Which non-pharmacological therapies are used** | | |
| Music | 148 (21.0%) | 63 (24.2%) |
| Mobilization | 492 (70.7%) | 192 (73.8%) |
| Cognitive stimulation / Occupational therapy | 312 (44.5%) | 128 (49.2%) |
| Family engagement | 532 (75.4%) | 198 (76.2%) |

^a^Confusion Assessment Method for the Intensive Care Unit (CAM-ICU); ^b^Intensive care Delirium Screening Checklist (ICDSC); ^c^Mini-mental State Exam (MMSE); ^d^p<0.05; ^e^Atypical Antipsychotics (AA);

**Table S21** Univariable analysis monitoring pain for patients able to communicate during the COVID-19 pandemic

| Variables | Monitoring pain | | p-value |
| --- | --- | --- | --- |
|  | Yes n (%) | No n (%) |  |
| Intensive care specialist |  |  | <0.001^a^ |
| Yes | 195 (66.8%) | 21 (7.1%) |  |
| No | 51 (17.5%) | 25 (8.6%) |  |
| Daily rounds with an intensive care specialist |  |  | 0.006^a^ |
| Yes | 230 (79.0%) | 37 (12.7%) |  |
| No | 15 (5.2%) | 9 (3.1%) |  |
| Type of hospital |  |  | 0.006^a^ |
| Public hospital | 88 (30.3%) | 27 (9.3%) |  |
| Private hospital | 67 (23.1%) | 10 (3.5%) |  |
| University hospital / Teaching hospital | 90 (31.0%) | 8 (2.8%) |  |
| Pharmacist participates in multidisciplinary rounds |  |  | 0.01^a^ |
| Yes | 86 (29.4%) | 7 (2.4%) |  |
| No | 160 (54.8%) | 38 (13.4%) |  |
| Doctor participates in multidisciplinary rounds |  |  | 0.02^a^ |
| Yes | 244 (83.6%) | 43 (14.7%) |  |
| No | 2 (0.7%) | 3 (1.0%) |  |
| Estimated frequency of patients using mechanical ventilation |  |  | 0.02^a^ |
| <20% | 17 (5.8%) | 7 (2.4%) |  |
| 20-40% | 52 (17.9%) | 7 (2.4%) |  |
| 41-70% | 121 (41.6%) | 15 (5.2%) |  |
| >70% | 55 (18.9%) | 17 (5.8%) |  |
| Nutritionist participates in multidisciplinary rounds |  |  | 0.05^a^ |
| Yes | 98 (33.6 %) | 11 (3.8%) |  |
| No | 148 (50.7%) | 35 (11.9%) |  |
| Nurse:patient rate (daytime) |  |  | 0.05^a^ |
| 1:1 | 14 (4.9%) | 3 (1.0%) |  |
| 1:2 | 74 (25.7%) | 8 (2,8%) |  |
| 1:3 | 80 (27.8%) | 14 (4.9%) |  |
| 1:4 | 33 (11.5%) | 4 (1.4%) |  |
| 1:5 | 18 (6.3%) | 8 (2.8%) |  |
| >1:5 | 26 (8.9%) | 6 (2.0%) |  |
| There was an analgesia protocol in intensive care unit |  |  | 0.09^a^ |
| Yes | 169 (57.9%) | 24 (8.2%) |  |
| No | 66 (22.6%) | 20 (6.8%) |  |
| I don’t know | 11 (3.8%) | 2 (0.7%) |  |
| Physiotherapist participates in multidisciplinary rounds |  |  | 0.07^a^ |
| Yes | 193 (66.1%) | 30 (10.3%) |  |
| No | 53 (18.1%) | 16 (5.5%) |  |
| Nurse:patient rate (nighttime) |  |  | 0.2 |
| 1:1 | 14 (4.9%) | 1 (0.3%) |  |
| 1:2 | 74 (25.7%) | 10 (3.5%) |  |
| 1:3 | 80 (27.8%) | 11 (3.8%) |  |
| 1:4 | 33 (11.5%) | 8 (2.8%) |  |
| 1:5 | 18 (6.3%) | 7 (2.4%) |  |
| >1:5 | 26 (8.9%) | 6 (2.1%) |  |
| Time working in an intensive care |  |  | 0.54 |
| Up to 10 years | 140 (48.0%) | 29 (5.8%) |  |
| Over 10 years | 106 (36.3%) | 17 (9.9%) |  |
| Nurse participates in multidisciplinary rounds |  |  | 0.6 |
| Yes | 221 (75.7%) | 40 (13.7%) |  |
| No | 25 (8.6%) | 6 (2.0%) |  |
| Experience as intensive care specialis |  |  | 0.9 |
| Up to 10 years | 117 (54.2 %) | 12 (5.5%) |  |
| Over 10 years | 78 (36.1 %) | 9 (4.2%) |  |

^a^Variables included of the logistic regression model;

**Table S22** variable independently associated with monitoring pain for patients able to communicate during the COVID-19 pandemic

| Variables | OR | CI (2.5% - 97.5%) | p-value |
| --- | --- | --- | --- |
| Intensive care specialist | 3.53 | 1.51-8.22 | 0.003 |

**Table S23** Univariable analysis monitoring pain for patients unable to communicate during the COVID-19 pandemic

| Variables | Monitoring pain | | p-value |
| --- | --- | --- | --- |
|  | Yes n (%) | No n (%) |  |
| Intensive care specialist |  |  | <0.001^a^ |
| Yes | 164 (58%) | 48 (17.0%) |  |
| No | 38 (13.4%) | 33 (11.6%) |  |
| Estimated frequency of patients using mechanical ventilation |  |  | <0.001^a^ |
| <20% | 10 (3.6%) | 13 (4.6%) |  |
| 20-40% | 39 (13.8%) | 17 (6.0%) |  |
| 41-70% | 198 (38.3%) | 24 (8.5%) |  |
| >70% | 45 (16.0%) | 26 (9.2%) |  |
| There was an analgesia protocol in intensive care unit |  |  | <0.001^a^ |
| Yes | 146 (51.6%) | 41 (14.5%) |  |
| No | 47 (16.6%) | 37 (13.1%) |  |
| I don’t know | 9 (3.2%) | 3 (1.0%) |  |
| Pharmacist participates in multidisciplinary rounds |  |  | 0.002^a^ |
| Yes | 76 (26.9 %) | 15 (5.3%) |  |
| No | 126 (44.5%) | 66 (23.3%) |  |
| Type of hospital |  |  | 0.006^a^ |
| Public hospital | 70 (24.9%) | 41 (%) |  |
| Private hospital | 51 (23.1 %) | 21 (%) |  |
| University hospital / Teaching hospital | 81 (28.8 %) | 17 (%) |  |
| Daily rounds with an intensive care specialist |  |  | 0.01^a^ |
| Yes | 230 (79.3%) | 69 (24.5%) |  |
| No | 10 (3.5%) | 11 (3.9%) |  |
| Nurse:patient rate (daytime) |  |  | 0.04^a^ |
| 1:1 | 13 (4.7%) | 4 (1.5%) |  |
| 1:2 | 84 (30.2%) | 18 (6.5%) |  |
| 1:3 | 52 (18.7%) | 25 (9.0%) |  |
| 1:4 | 22 (7.9%) | 9 (3.2%) |  |
| 1:5 | 16 (5.8%) | 9 (3.2%) |  |
| >1:5 | 14 (5.0%) | 12 (4.3%) |  |
| Physiotherapist participates in multidisciplinary rounds |  |  | 0.08^a^ |
| Yes | 161 (56.9%) | 56 (19.8%) |  |
| No | 41 (14.5%) | 25 (8.8%) |  |
| Nurse:patient rate (nighttime) |  |  | 0.11^a^ |
| 1:1 | 11(3.9%) | 3 (1.1%) |  |
| 1:2 | 62 (22.2%) | 18 (6.5%) |  |
| 1:3 | 70 (25.1%) | 20 (7.2%) |  |
| 1:4 | 26 (9.3%) | 14 (5.0%) |  |
| 1:5 | 16 (5.7%) | 8 (2.9%) |  |
| >1:5 | 17 (6.1%) | 14 (5.0%) |  |
| Nurse participates in multidisciplinary rounds |  |  | 0.12^a^ |
| Yes | 185 (65.4 %) | 69 (24.4%) |  |
| No | 17 (6.0%) | 12 (4.2%) |  |
| Doctor participates in multidisciplinary rounds |  |  | 0.14^a^ |
| Yes | 200 (70.7%) | 78 (27.6%) |  |
| No | 2 (0.7%) | 3 (1.0%) |  |
| Nutritionist participates in multidisciplinary rounds |  |  | 0.26 |
| Yes | 81 (28.6 %) | 26 (9.2%) |  |
| No | 121 (42.8%) | 55 (19.4%) |  |
| Experience as intensive care specialist |  |  | 0.54 |
| Up to 10 years | 99 (46.7%) | 26 (12.2%) |  |
| Over 10 years | 65 (30.7%) | 22 (10.4%) |  |
| Time working in an intensive |  |  | 0.85 |
| Up to 10 years | 113 (40.0 %) | 47 (16.6%) |  |
| Over 10 years | 89 (31.4 %) | 34 (12.0%) |  |

^a^Variables included of the logistic regression model;

**Table S24** Variables independently associated with monitoring pain for patients unable to communicate during the COVID-19 pandemic

| Variables | OR | CI (2.5%-97.5%) | p-value |
| --- | --- | --- | --- |
| Intensive care specialist | 2.63 | 1.24-5.57 | 0.01 |
| Estimated frequency of patients using mechanical ventilation – 40-70% | 4.94 | 1.60-15.20 | 0.005 |
| Pharmacist participate on multidisciplinary rounds | 2.31 | 1.08-4.96 | 0.03 |

**Table S25** Univariable analysis monitoring pain for patients unable to communicate using structured tools during the COVID-19 pandemic

| Variables (n) | Use structured tools | | p-value |
| --- | --- | --- | --- |
|  | Yes n (%) | No n (%) |  |
| Intensive care specialist | | | <0.001^a^ |
| Yes | 164 (58.0 %) | 48 (17%) |  |
| No | 38 (13.4 %) | 33 (11.6%) |  |
| Physiotherapist participates on multidisciplinary rounds | | | 0.009^a^ |
| Yes | 145 (71.8 %) | 16 (8.0 %) |  |
| No | 3 (14.8 %) | 11 (5.4 %) |  |
| Daily rounds with an intensive care specialist |  |  | 0.02^a^ |
| Yes | 169 (83.7%) | 23 (11.3%) |  |
| No | 6 (3.0 %) | 4 (2.0 %) |  |
| Nursing:patient rate (nighttime) | | | 0.06^a^ |
| 1:1 | 8 (3.9 %) | 3 (1.4 %) |  |
| 1:2 | 57 (28.2 %) | 5 (2.5 %) |  |
| 1:3 | 64 (31.7 %) | 6 (2.9 %) |  |
| 1:4 | 22 (10.9 %) | 4 (2.0 %) |  |
| 1:5 | 12 (6.0 %) | 4 (2.0 %) |  |
| >1:5 | 12 (6.0 %) | 5 (2.5 %) |  |
| There was an analgesia protocol in intensive care unit |  |  | 0.12^a^ |
| Yes | 129 (63.9 %) | 17 (8.4 %) |  |
| No | 37 (18.3 %) | 10 (4.9 %) |  |
| I don’t know | 9 (4.5 %) | 0 (0.0 %) |  |
| Nursing:patient rate (daytime) |  |  | 0.12^a^ |
| 1:1 | 10 (4.9 %) | 3 (1.5 %) |  |
| 1:2 | 78 (38.8 %) | 6 (3.0 %) |  |
| 1:3 | 45 (22.4 %) | 7 (3.5 %) |  |
| 1:4 | 19 (9.5 %) | 3 (1.5 %) |  |
| 1:5 | 12 (6.0 %) | 4 (2.0 %) |  |
| >1:5 | 10 (4.9 %) | 4 (2.0 %) |  |
| Estimated frequency of patients using mechanical ventilation | | | 0.25 |
| <20% (10) | 7 (3.5 %) | 3 (1.5 %) |  |
| 20-40% (39) | 35 (17.3 %) | 4 (2. 0%) |  |
| 41-70% () | 96 (47.5 %) | 12 (5.9 %) |  |
| >70% (45) | 37 (18.3 %) | 8 (4. 0%) |  |
| Pharmacist participates on multidisciplinary rounds | | | 0.25 |
| Yes | 69 (34.2 %) | 7 (3.5 %) |  |
| No | 106 (52.5 %) | 20 (9.9%) |  |
| Doctor participates on multidisciplinary rounds | | | 0.25 |
| Yes | 174 (86.1%) | 26 (12.9 %) |  |
| No | 1 (0.5 %) | 1 (0.5 %) |  |
| Nurse participates on multidisciplinary rounds |  |  | 0.25 |
| Yes | 162 (80.2 %) | 23 (11.4 %) |  |
| No | 13 (6.4 %) | 4 (2 %) |  |
| Type of hospital |  |  | 0.27 |
| Public hospital | 57 (28.2 %) | 13 (6.4 %) |  |
| Private hospital | 45 (22.3 %) | 6 (3.0 %) |  |
| University hospital / Teaching hospital | 53 (36.1 %) | 8 (4.0 %) |  |
| Time working in intensive | | | 0.27 |
| Up to 10 years | 101 (50.0 %) | 12 (6.0 %) |  |
| More than 10 years | 74 (36.6%) | 15 (7.4 %) |  |
| Nutritionist participates on multidisciplinary rounds | | | 0.32 |
| Yes | 73 (36.1 %) | 8 (4.0 %) |  |
| No | 102 (50.5 %) | 19 (9.4 %) |  |
| Time being intensive care specialist care | | | 0.48 |
| Up to 10 years | 90 (54.9 %) | 9 (5.5 %) |  |
| More than 10 years | 56 (34.1 %) | 9 (5.5 %) |  |

**Table S26** variable independently associated with monitoring pain for patients unable to communicate using structured tools during the COVID-19 pandemic

| Variables | OR | CI (2.5 % - 97.5 %) | p-value |
| --- | --- | --- | --- |
| Intensive care specialist | 2.54 | 1.28 - 5.04 | 0.007 |

**Table S27** Univariable analysis non-pharmacological treatment for pain during the COVID-19 pandemic

| Variables (n) | Use non-pharmacological treatment | | p-value |
| --- | --- | --- | --- |
|  | Yes | No n (%) |  |
| Nursing:patient rate (daytime) | | | <0.001^a^ |
| 1:1 | 3 (1.1 %) | 14 (5.2 %) |  |
| 1:2 | 51 (18.8 %) | 49 (18.1 %) |  |
| 1:3 | 41 (15.1 %) | 32 (11.8 %) |  |
| 1:4 | 9 (3.3 %) | 22 (8.1 %) |  |
| 1:5 | 6 (2.2 %) | 19 (7.0 %) |  |
| >1:5 | 8 (3.0 %) | 17 (6.3 %) |  |
| Intensive care specialist | | | 0.001^a^ |
| Yes | 101 (36.6 %) | 106 (38.4 %) |  |
| No | 18 (6.5 %) | 51 (18.5 %) |  |
| Nursing:patient rate (nighttime) | | | 0.004^a^ |
| 1:1 | 2 (0.7 %) | 12 (4.4 %) |  |
| 1:2 | 42 (15.4 %) | 37 (13.6 %) |  |
| 1:3 | 45 (16.5 %) | 41 (15.1 %) |  |
| 1:4 | 14 (5.2 %) | 25 (9.2 %) |  |
| 1:5 | 6 (2.3 %) | 18 (6.6 %) |  |
| >1:5 | 9 (3.3 %) | 21 (7.7 %) |  |
| Physiotherapist participates on multidisciplinary rounds | | | 0.01^a^ |
| Yes | 101 (36.6 %) | 112 (40.6 %) |  |
| No | 18 (6.5 %) | 45 (16. 3%) |  |
| There was an analgesia protocol in intensive care unit | | | 0.03^a^ |
| Yes | 88 (31.9 %) | 96 (34.8 %) |  |
| No | 29 (10.5 %) | 51 (18.5 %) |  |
| I don’t know | 2 (0.7 %) | 10 (3.6 %) |  |
| Estimated frequency of patients using mechanical ventilation | | | 0.22 |
| <20% | 9 (3.2 %) | 14 (5 %) |  |
| 20-40% | 29 (10.6%) | 23 (8.4 %) |  |
| 41-70% | 52 (18.9 %) | 78 (28.4 %) |  |
| >70% | 28 (10.2 %) | 42 (15.3 %) |  |
| Time being intensive care specialist care | | | 0.26 |
| Up to 10 years | 64 (31 %) | 58 (28.0 %) |  |
| More than 10 years | 37 (17.8 %) | 48 (23.2 %) |  |
| Daily rounds with an intensive care specialist |  |  | 0.32 |
| Yes | 112 (52.0 %) | 143 (40.7 %) |  |
| No | 6 (5.1 %) | 14 (2.2 %) |  |
| Pharmacist participates on multidisciplinary rounds | | | 0.58 |
| Yes | 41 (14.9 %) | 48 (17.4 %) |  |
| No | 78 (28.3 %) | 109 (39.4 %) |  |
| Type of hospital |  |  | 0.63 |
| Public hospital | 45 (16.4 %) | 63 (23.0 %) |  |
| Private hospital | 28 (10.2 %) | 42 (15.4 %) |  |
| University hospital / Teaching hospital | 45 (16.4 %) | 51 (18.6 %) |  |
| Nurse participates on multidisciplinary rounds |  |  | 0.69 |
| Yes | 108 (39.1 %) | 140 (50.7 %) |  |
| No | 11 (4.0 %) | 17 (6.2 %) |  |
| Nutritionist participates on multidisciplinary rounds | | | 0.75 |
| Yes | 47 (17.0 %) | 58 (21.0 %) |  |
| No | 72 (26.1 %) | 99 (35.9 %) |  |
| Doctor participates on multidisciplinary rounds | | | 1 |
| Yes | 117 (42.4 %) | 154 (55.8 %) |  |
| No | 2 (0.7 %) | 3 (1.1 %) |  |
| Time working in intensive | | | 1 |
| Up to 10 years | 67 (24.3 %) | 88 (31.9 %) |  |
| More than 10 years | 52 (18.8 %) | 69 (25.0 %) |  |

^a^Variables included of the logistic regression model;

**Table S28** Variables independently associated with non-pharmacological treatment for pain during the COVID-19 pandemic

| Variables | OR | CI (2.5 % - 97.5 %) | p-value |
| --- | --- | --- | --- |
| Intensive care specialist | 2.57 | 1.30 - 5.06 | 0.001 |
| Physiotherapist participate on multidisciplinary rounds | 2.42 | 1.24 - 4.72 | 0.009 |

**Table S29** Univariable analysis of the sedation scale use to evaluate sedation levels during the COVID-19 pandemic

| Variables (n) | Use sedation scale | | p-value |
| --- | --- | --- | --- |
|  | Yes n (%) | No n (%) |  |
| There was a sedation protocol in intensive care unit |  |  | <0.001^a^ |
| Yes | 178 (66.4 %) | 11 (4.1 %) |  |
| No | 49 (18.3 %) | 19 (7.1 %) |  |
| I don’t know | 5 (1.9 %) | 6 (2.2 %) |  |
| There was an analgesia protocol in intensive care unit |  |  | <0.001^a^ |
| Yes | 169 (63.1 %) | 10 (3.7 %) |  |
| No | 56 (20.9 %) | 21 (7.8 %) |  |
| I don’t know | 7 (2.6 %) | 5 (1.9 %) |  |
| Intensive care specialist |  |  | 0.001^a^ |
| Yes | 101 (%) | 106 (%) |  |
| No | 18 (%) | 51(%) |  |
| Estimated frequency of patients using mechanical ventilation |  |  | 0.01^a^ |
| <20% | 15 (5.6 %) | 8 (3.0 %) |  |
| 20-40% | 44 (16.5 %) | 5 (1.9 %) |  |
| 41-70% | 111 (41.6 %) | 15 (5.6 %) |  |
| >70% | 61 (22.8 %) | 8 (3.0 %) |  |
| Frequency of discussion about sedation goals |  |  | 0.09^a^ |
| Daily | 203 (75.7 %) | 24 (9.0 %) |  |
| Sometimes | 24 (9.0 %) | 10 (3.7 %) |  |
| Never | 5 (1.9 %) | 2 (0.7 %) |  |
| Pharmacist participates in multidisciplinary rounds |  |  | 0.10^a^ |
| Yes | 80 (29.9 %) | 7 (2.6 %) |  |
| No | 152 (56.7 %) | 29 (10.8 %) |  |
| Type of hospital |  |  | 0.12^a^ |
| Public hospital | 88 (33.1 %) | 16 (6.0 %) |  |
| Private hospital | 57 (21.4 %) | 12 (4.5 %) |  |
| University / Teaching hospital | 86 (32.4 %) | 7 (2.6 %) |  |
| Physiotherapist participates in multidisciplinary rounds |  |  | 0.17^a^ |
| Yes | 182 (67.9 %) | 24 (9.0 %) |  |
| No | 50 (18.6 %) | 12 (4.5 %) |  |
| Nurse:patient rate (daytime) | | | 0.21 |
| 1:1 | 14 (5.3 %) | 2 (0.8 %) |  |
| 1:2 | 88 (33.5 %) | 10 (3.8 %) |  |
| 1:3 | 63 (24.0 %) | 7 (2.6 %) |  |
| 1:4 | 27 (10.3 %) | 4 (1.5 %) |  |
| 1:5 | 17 (6.5 %) | 7 (2.6 %) |  |
| >1:5 | 20 (7.6 %) | 4 (1.5 %) |  |
| Time working in intensive care un |  |  | 0.45 |
| Up to 10 years | 129 (48.1 %) | 23 (8.6 %) |  |
| Over 10 years | 103 (38.4 %) | 13 (4.9 %) |  |
| Daily rounds with an intensive care specialist |  |  | 0.49 |
| Yes | 215 (80.5 %) | 32 (12 %) |  |
| No | 16 (6.0 %) | 4 (1.5 %) |  |
| Nurse:patient rate (nighttime) | | | 0.58 |
| 1:1 | 12 (4.5%) | 2 (0.8 %) |  |
| 1:2 | 70 (26.5 %) | 8 (3.0 %) |  |
| 1:3 | 76 (28.8 %) | 8 (3.0 %) |  |
| 1:4 | 30 (11.4 %) | 6 (2.3 %) |  |
| 1:5 | 19 (7.2 %) | 4 (1.5 %) |  |
| >1:5 | 23 (8.7 %) | 6 (2.3 %) |  |
| Doctor participates in multidisciplinary rounds |  |  | 0.6 |
| Yes | 277 (84.7 %) | 36 (13.4 %) |  |
| No | 5 (1.9 %) | 0 (0.0 %) |  |
| Nutritionist participates in multidisciplinary rounds |  |  | 0.75 |
| Yes | 91 (34.0 %) | 10 (3.7 %) |  |
| No | 141 (52.6 %) | 26 (9.7 %) |  |
| Experience as an intensive care specialist | | | 1 |
| Up to 10 years | 108 (53.7 %) | 7 (5 %) |  |
| Over 10 years | 76 (37.8 %) | 10 (3.5 %) |  |
| Nurse participates in multidisciplinary rounds |  |  | 1 |
| Yes | 209 (78.0 %) | 32 (11.9 %) |  |
| No | 23 (8.6 %) | 4 (1.5 %) |  |

^a^Variables included in the logistic regression model;

**Table S30** Variables independently associated with sedation scale use to evaluate sedation levels during the COVID-19 pandemic

| Variables | OR | CI (2.5 % - 97.5 %) | p-value |
| --- | --- | --- | --- |
| Intensive care specialist | 3.09 | 1.24 - 7.70 | 0.01 |
| Frequency of mechanical ventilation: 40% - 70% | 4.76 | 1.41 – 16.11 | 0.01 |
| Frequency of mechanical ventilation: > 70% | 6.2 | 1.48 - 27.57 | 0.01 |

**Table S31** Univariable analysis of the non-routine use of mechanical restraints for patients under MV during the COVID-19 pandemic

| Variables (n) | Order mechanical restraint | | p-value |
| --- | --- | --- | --- |
|  | Routine n (%) | Non-routine n (%) |  |
| Intensive care specialist |  |  | <0.001^a^ |
| Yes | 39 (14.9 %) | 157 (60.0 %) |  |
| No | 28 (10.7 %) | 38 (14.5 %) |  |
| Time working in intensive |  |  | <0.001^a^ |
| Up to 10 years | 51 (19.5 %) | 100 (38.1 %) |  |
| More than 10 years | 16 (6.1 %) | 95 (36.3 %) |  |
| Time being intensive care specialist care |  |  | 0.005^b^ |
| Up to 10 years | 31 (15.8 %) | 84 (42.9 %) |  |
| More than 10 years | 8 (4.1 %) | 73 (37.2 %) |  |
| Pharmacist participates on multidisciplinary rounds |  |  | 0.03^a^ |
| Yes | 14 (5.3 %) | 70 (26.8 %) |  |
| No | 53 (20.2 %) | 125 (47.7 %) |  |
| Nursing:patient rate (daytime) |  |  | 0.11^a^ |
| 1:1 | 1 (0.4 %) | 15 (5.8 %) |  |
| 1:2 | 22 (8.6 %) | 74 (28.8 %) |  |
| 1:3 | 22 (8.6 %) | 45 (17.5 %) |  |
| 1:4 | 5 (2.0 %) | 25 (9.7 %) |  |
| 1:5 | 9 (3.5 %) | 15 (5.8 %) |  |
| >1:5 | 1 (3.1 %) | 16 (6.2 %) |  |
| Nurse participates on multidisciplinary rounds |  |  | 0.17^a^ |
| Yes | 57 (21.8 %) | 179 (68.3 %) |  |
| No | 10 (3.8 %) | 16 (6.1%) |  |
| Estimated frequency of patients using mechanical ventilation |  |  | 0.19^a^ |
| <20% | 2 (0.8 %) | 20 (7.7 %) |  |
| 20-40% | 10 (3.8 %) | 36 (13.8 %) |  |
| 41-70% | 34 (13.0 %) | 91 (34.9 %) |  |
| >70% | 21 (8.0 %) | 47 (18 %) |  |
| Nursing:patient rate (nighttime) |  |  | 0.21 |
| 1:1 | 0 (0.0 %) | 14 (5.4 %) |  |
| 1:2 | 18 (7.0 %) | 58 (22.5 %) |  |
| 1:3 | 24 (9.3 %) | 57 (22.1 %) |  |
| 1:4 | 7 (2.7 %) | 28 (10.9 %) |  |
| 1:5 | 8 (3.1 %) | 15 (5.8 %) |  |
| >1:5 | 10 (3.9 %) | 19 (7.3 %) |  |
| There was a sedation protocol in intensive care unit |  |  | 0.35 |
| Yes | 41 (15.6 %) | 142 (54.2 %) |  |
| No | 23 (8.8 %) | 45 (17.2 %) |  |
| I don’t know | 3 (1.1 %) | 11 (3.1 %) |  |
| Frequency of discussion about sedation goals |  |  | 0.38 |
| Daily | 53 (20.2 %) | 168 (64.1 %) |  |
| Sometimes | 11 (4. 2%) | 23 (8.8 %) |  |
| Never | 2 (1.2 %) | 4 (1. 5%) |  |
| There was an analgesia protocol in intensive care unit |  |  | 0.44 |
| Yes | 40 (15.3 %) | 153 (50.8 %) |  |
| No | 24 (9.2 %) | 53 (20.2 %) |  |
| I don’t know | 3 (1.1 %) | 9 (3.4 %) |  |
| Physiotherapist participates on multidisciplinary rounds |  |  | 0.58 |
| Yes | 49 (6.9 %) | 151 (57.6 %) |  |
| No | 18 (18.7 %) | 44 (16.8 %) |  |
| Type of hospital |  |  | 0.82 |
| Public hospital | 28 (10.8 %) | 74 (28.5 %) |  |
| Private hospital | 17 (6.5 %) | 48 (18.4 %) |  |
| University hospital / Teaching hospital | 22 (8.5 %) | 71 (27.3 %) |  |
| Daily rounds with an intensive care specialist |  |  | 0.49 |
| Yes | 215 (80.5 %) | 32 (12 %) |  |
| No | 16 (6.0 %) | 4 (1.5 %) |  |
| Doctor participates on multidisciplinary rounds |  |  | 0.61 |
| Yes | 65 (24.8 %) | 192 (73.3 %) |  |
| No | 2 (0.7 %) | 3 (1.2 %) |  |
| Nutritionist participates on multidisciplinary rounds |  |  | 0.75 |
| Yes | 18 (6.9 %) | 79 (30.1 %) |  |
| No | 49 (18.7%) | 116 (44.3 %) |  |

^a^Variables included of the logistic regression model; ^b^Variable excluded of the logistic regression model due to multicollinearity.

**Table S32** variable independently associated with non-routine use of mechanical restraints for patients under MV during the COVID-19 pandemic

| Variables | OR | CI (2.5 % - 97.5 %) | p-value |
| --- | --- | --- | --- |
| Intensive care specialist | 2.34 | 1.14 - 4.83 | 0.02 |

**Table S33** Univariable analysis delirium investigation during the COVID-19 pandemic

| Variables | Delirium investigation | | p-value |
| --- | --- | --- | --- |
|  | Yes n (%) | No n (%) |  |
| Know delirium frequency |  |  | <0.001^a^ |
| Yes | 82 (31.3%) | 1 (0.4%) |  |
| No | 136 (51.9%) | 43 (16.4%) |  |
| There was a sedation protocol in intensive care unit |  |  | 0.03^a^ |
| Yes | 159 (60.7%) | 24 (9.2%) |  |
| No | 52 (19.8%) | 16 (6.1%) |  |
| I don’t know | 7 (2.7%) | 4 (1.5%) |  |
| Intensive care specialist |  |  | 0.03 |
| Yes | 169 (64.5%) | 27 (10.3%) |  |
| No | 49 (18.7%) | 17 (6.5%) |  |
| Nurse:patient rate (daytime) |  |  | 0.03^a^ |
| 1:1 | 11 (4.3%) | 5 (1.9%) |  |
| 1:2 | 83 (32.3%) | 13 (5.1%) |  |
| 1:3 | 61 (23.7%) | 6 (2.3%) |  |
| 1:4 | 23 (9.0%) | 7 (2.7%) |  |
| 1:5 | 20 (7.8%) | 4 (1.6%) |  |
| >1:5 | 16 (6.2%) | 8 (3.1%) |  |
| Frequency of discussion about sedation goals |  |  | 0.10^a^ |
| Daily | 187 (71.4%) | 34 (13.0%) |  |
| Sporadically | 27 (10.3%) | 7 (2.7%) |  |
| Never | 4 (1.5%) | 3 (1.1%) |  |
| Daily rounds with an intensive care specialist |  |  | 0.35 |
| Yes | 202 (77.4%) | 39 (15.0%) |  |
| No | 15 (5.7%) | 5 (1.9%) |  |
| Nurse participates in multidisciplinary rounds |  |  | 0.09^a^ |
| Yes | 193 (73.7%) | 43 (16.4%) |  |
| No | 25 (9.5%) | 1 (0.4%) |  |
| Nursing:patient rate (nighttime) |  |  | 0.11^a^ |
| 1:1 | 10 (3.9%) | 4 (1.5%) |  |
| 1:2 | 65 (25.2%) | 11 (4.2%) |  |
| 1:3 | 73 (28.3%) | 8 (3.1%) |  |
| 1:4 | 28 (10.9%) | 7 (2.7%) |  |
| 1:5 | 19 (7.4%) | 4 (1.5%) |  |
| >1:5 | 20 (7.8%) | 9 (3.5%) |  |
| Type of hospital |  |  | 0.43 |
| Public hospital | 81 (31.2%) | 21 (8.1%) |  |
| Private hospital | 55 (21.1%) | 10 (3.8%) |  |
| University hospital / Teaching hospital | 80 (30.8%) | 13(5.0%) |  |
| Physiotherapist participates in multidisciplinary rounds |  |  | 0.47 |
| Yes | 164 (62.6%) | 36 (13.7%) |  |
| No | 64 (20.6%) | 8 (3.1%) |  |
| Pharmacist participates in multidisciplinary rounds |  |  | 0.56 |
| Yes | 72 (27.5%) | 12 (4.6%) |  |
| No | 146 (55.7%) | 32 (12.2%) |  |
| Nutritionist participates in multidisciplinary rounds |  |  | 0.78 |
| Yes | 82 (31.3%) | 15 (5.7%) |  |
| No | 136 (51.9%) | 29 (11.1%) |  |
| Experience as intensive care specialist |  |  | 0.78 |
| Up to 10 years | 98 (50.0%) | 17 (8.7%) |  |
| Over 10 years | 71 (36.2%) | 10 (5.1%) |  |
| Estimated frequency of patients using mechanical ventilation |  |  | 0.84 |
| <20% | 17 (6.5%) | 5 (1.9%) |  |
| 20-40% | 39 (15.0%) | 7 (2.7%) |  |
| 41-70% | 103 (39.5%) | 22 (8.4%) |  |
| >70% | 17 (22.2%) | 10 (3.8%) |  |
| Doctor participates in multidisciplinary rounds |  |  | 1 |
| Yes | 214 (81.7%) | 43 (16.4%) |  |
| No | 4 (1.5%) | 1 (0.4%) |  |
| Time working in an intensive care unit |  |  | 1 |
| Up to 10 years | 126 (48.1%) | 25 (9.5%) |  |
| Over 10 years | 92 (35.1%) | 19 (7.3%) |  |

^a^Variables included of the logistic regression model;

**Table S34** variable independently associated with delirium investigation during the COVID-19 pandemic

| Variables | OR | CI (2.5 % - 97.5 %) | p-value |
| --- | --- | --- | --- |
| Know delirium frequency | 25.64 | 3.35-196.05 | 0.001 |

**Table S35** Univariable analysis delirium investigation using structured tools during the COVID-19 pandemic

| Variables | Use structured tools | | p-value |
| --- | --- | --- | --- |
|  | Yes n (%) | No n (%) |  |
| Know delirium frequency |  |  | 0.001^a^ |
| Yes | 67 (31.0%) | 14 (6.5%) |  |
| No | 82 (38.0%) | 53 (24.5%) |  |
| Frequency of discussion about sedation goals |  |  | 0.02^a^ |
| Daily | 134 (62.1%) | 51 (23.6%) |  |
| Sporadically | 13 (6.0%) | 14 (6.5%) |  |
| Never | 2 (0.9%) | 2 (0.9%) |  |
| Intensive care specialist |  |  | 0.04 |
| Yes | 122 (56.5%) | 46 (21.3%) |  |
| No | 27 (12.5%) | 21 (9.7%) |  |
| Estimated frequency of patients using mechanical ventilation |  |  | 0.05^a^ |
| <20% | 7 (3.3%) | 10 (4.6%) |  |
| 20-40% | 27 (12.6%) | 12 (5.6%) |  |
| 41-70% | 76 (35.3%) | 26 (12.1%) |  |
| >70% | 38 (17.7%) | 19(8.8%) |  |
| Daily rounds with an intensive care specialist |  |  | 0.07^a^ |
| Yes | 141 (65.6%) | 59 (27.4%) |  |
| No | 7 (3.3%) | 8 (3.7%) |  |
| Nurse:patient rate (daytime) |  |  | 0.06^a^ |
| 1:1 | 9 (4.2%) | 2 (1.0%) |  |
| 1:2 | 54 (25.5%) | 27 (12.7%) |  |
| 1:3 | 36 (17.0%) | 25 (11.8%) |  |
| 1:4 | 21 (9.9%) | 2 (1.0%) |  |
| 1:5 | 13 (6.1%) | 7 (3.3%) |  |
| >1:5 | 13 (6.1%) | 3 (1.4%) |  |
| Nurse:patient rate (nighttime) |  |  | 0.07^a^ |
| 1:1 | 7 (3.3%) | 3 (1.4%) |  |
| 1:2 | 47 (22.1%) | 16 (7.5%) |  |
| 1:3 | 40 (18.8%) | 33 (15.5%) |  |
| 1:4 | 23 (10.8%) | 5 (2.3%) |  |
| 1:5 | 14 (6.6%) | 5 (2.3%) |  |
| >1:5 | 15 (7.1%) | 5 (2.3%) |  |
| Nurse participates in multidisciplinary rounds |  |  | 0.13 |
| Yes | 128 (59.3%) | 63 (21.2%) |  |
| No | 21 (9.7%) | 4 (1.8%) |  |
| Physiotherapist participates in multidisciplinary rounds |  |  | 0.14^a^ |
| Yes | 107 (49.5%) | 55 (25.5%) |  |
| No | 42 (19.5%) | 12 (5.5%) |  |
| There was a sedation protocol in intensive care unit |  |  | 0.24 |
| Yes | 113 (52.3%) | 44 (20.4%) |  |
| No | 31 (14.4%) | 21 (9.7%) |  |
| I don’t know | 5 (2.3%) | 2 (0.9%) |  |
| Pharmacist participates in multidisciplinary rounds |  |  | 0.37 |
| Yes | 53 (24.5%) | 19 (8.8%) |  |
| No | 96 (44.5%) | 48 (22.2%) |  |
| Time working in an intensive care unit |  |  | 0.54 |
| Up to 10 years | 83 (38.4%) | 41 (19.0%) |  |
| Over 10 years | 66 (30.6%) | 26 (12.0%) |  |
| Type of hospital |  |  | 0.71 |
| Public hospital | 53 (24.8%) | 28 (13.1%) |  |
| Private hospital | 37 (17.3%) | 16 (7.5%) |  |
| University hospital / Teaching hospital | 57 (26.6%) | 23 (10.7%) |  |
| Experience as intensive care specialist |  |  | 0.74 |
| Up to 10 years | 69 (41.1%) | 28 (16.7%) |  |
| Over 10 years | 53 (31.5%) | 18 (10.7%) |  |
| Nutritionist participates in multidisciplinary rounds |  |  | 0.84 |
| Yes | 57 (26.4%) | 24 (11.1%) |  |
| No | 92 (42.6%) | 43 (19.9%) |  |
| Doctor participates in multidisciplinary rounds |  |  | 1 |
| Yes | 146 (67.6%) | 66 (30.5%) |  |
| No | 3 (1.4%) | 1 (0.5%) |  |

^a^Variables included of the logistic regression model;

**Table S36** variable independently associated with d non-pharmacological treatment use for hypoactive delirium management during the COVID-19 pandemic

| Variables | OR | CI (2.5 % - 97.5 %) | p-value |
| --- | --- | --- | --- |
| Know delirium frequency | 26.46 | 3.45-202.71 | 0.001 |
